# Supplementary material for: Novel adverse outcome pathways revealed by chemical genetics in a developing marine fish
Source: eLife. 2017 Jan 24;6:e20707. doi: 10.7554/eLife.20707 (PMC5302885; doi:10.7554/eLife.20707)
Supplement: Supplementary file 1. — (A) Read count data for a selection of genes expressed in distinct tissues. (B) Ten most regulated Ingenuity Pathway Analysis (Categories: Development, Lipid metabolism, Molecular transport) in high dose at stages E1-E6. #, number of molecules (C) Ingenuity Pathway Analysis. Top five pathways in low, pulse and high dose at all time points during and after embryonic exposure in the categories: Top canonical pathways, Molecular and cellular functions, Physiological system development and function and Cardiotoxicity. #, number of molecules (D) Ten most up- and down-regulated genes. Genes that are represented among the ten most at more than one stage are collapsed into one row. SP, swissprot; E1-E6, embryonic exposure; L1-L5, larval exposure; PMID, PubMed identification. (E) Regulation of differentially expressed genes involved in cardiogenesis SP, swissprot; GB, genebank; FC, fold change; E1-E6, embryonic exposure; C, control; H, high dose. (F) Manually curated list of genes involved in cardiac development and function and craniofacial development and bone and cartilage maintenance. SP, swissprot; GB, genebank; Ref, references; PMID: National Center for Biotechnology Information (NCBI) PubMed identification. (G) List of excitation contraction coupling genes examined. SP, swissprot; GB, genebank. (H) Regulation of differentially expressed genes involved in excitation contraction coupling in exposed haddock. SP, swissprot; GB, genebank; FC, fold change; E1-E6, embryonic exposure; C, control; H, high dose; L1-L5, larval exposure. (I) Regulation of differentially expressed genes involved in craniofacial development. SP, swissprot; GB, genebank; FC, fold change; E1-E6, embryonic exposure; C, control; H, high dose. (J) Regulation of differentially expressed myosin heavy chain genes. SP, swissprot; GB, genebank; FC, fold change; E1-E6, embryonic exposure C, control; H, high dose. (K) Regulation of differentially expressed key genes involved in osmoregulation. SP, swissprot; G [file elife-20707-supp1.doc]

**Supplementary file 1**

Supplementary file 1A

| **GB ID** | **Read count** | **Expressed** |
| --- | --- | --- |
| *atp2a1* | 9114 | skeletal muscle |
| *atp2a2* | 312 | heart |
| *myh1* | 2543 | skeletal muscle |
| *mylpf* | 2458 | skeletal muscle |
| *myh6/amhc* | 176 | heart |
| *pax6* | 1773 | neural tube |
| *nkx2.2* | 333 | neural tube |
| *nkx2.5* | 85 | heart |
| *bmp10* | 11 | heart |
| *bmp4* | 263 | heart, tailbut, eye, epidermis |
| *fhl2* | 65 | heart |
| *cacna1c* | 60 | heart |
| *kcnh2* | 11 | heart |
| *nppa* | 43 | heart |
| *nppb* | 35 | heart |

Supplementary file 1B

| **Stage** | **Main category** | **Sub category** | **Diseases or Functions Annotation** | **p-Value** | **Molecules** | **#** |
| --- | --- | --- | --- | --- | --- | --- |
| E1 | Development | Organ Morphology, Organismal Development, Organismal Injury and Abnormalities, Skeletal and Muscular Disorders, Skeletal and Muscular System Development and Function | abnormal morphology of quadriceps femoris | 0,000534 | COL12A1 | 1 |
| E1 | Development | Connective Tissue Development and Function, Connective Tissue Disorders, Nervous System Development and Function, Organ Morphology, Organismal Development, Organismal Injury and Abnormalities, Skeletal and Muscular Disorders, Skeletal and Muscular System Development and Function, Tissue Development | abnormal morphology of vertebral spinous process | 0,00267 | COL12A1 | 1 |
| E1 | Development | Connective Tissue Development and Function, Nervous System Development and Function, Organ Morphology, Organismal Development, Skeletal and Muscular System Development and Function, Tissue Development | morphology of vertebrae | 0,00341 | COL12A1,CYP1A1 | 2 |
| E1 | Development | Connective Tissue Development and Function, Organ Morphology, Organismal Development, Skeletal and Muscular System Development and Function, Tissue Development, Tissue Morphology | diameter of femur | 0,0048 | COL12A1 | 1 |
| E1 | Development | Connective Tissue Development and Function, Nervous System Development and Function, Organ Morphology, Organismal Development, Skeletal and Muscular System Development and Function, Tissue Development | size of vertebrae | 0,00746 | CYP1A1 | 1 |
| E1 | Development | Lymphoid Tissue Structure and Development, Organ Morphology, Organismal Development, Tissue Morphology | mass of thymus gland | 0,018 | CYP1A1 | 1 |
| E1 | Development | Connective Tissue Development and Function, Connective Tissue Disorders, Organ Morphology, Organismal Development, Organismal Injury and Abnormalities, Skeletal and Muscular Disorders, Skeletal and Muscular System Development and Function, Tissue Development | abnormal morphology of short femur | 0,028 | COL12A1 | 1 |
| E1 | Development | Digestive System Development and Function, Gastrointestinal Disease, Hepatic System Development and Function, Hepatic System Disease, Organ Morphology, Organismal Development, Organismal Injury and Abnormalities | hepatomegaly | 0,047 | CYP1A1 | 1 |
| E1 | Lipid metabolism | Lipid Metabolism, Small Molecule Biochemistry, Vitamin and Mineral Metabolism | production of 2-hydroxyestradiol | 0,000534 | CYP1A1 | 1 |
| E1 | Lipid metabolism | Lipid Metabolism, Small Molecule Biochemistry | demethylation of 2-hydroxyestradiol | 0,00107 | CYP1A1 | 1 |
| E1 | Lipid metabolism | Lipid Metabolism, Small Molecule Biochemistry | metabolism of 20-hydroxyeicosatetraenoic acid | 0,00214 | CYP1A1 | 1 |
| E1 | Lipid metabolism | Endocrine System Development and Function, Lipid Metabolism, Small Molecule Biochemistry, Vitamin and Mineral Metabolism | metabolism of 17-alpha-ethinylestradiol | 0,00267 | CYP1A1 | 1 |
| E1 | Lipid metabolism | Energy Production, Lipid Metabolism, Small Molecule Biochemistry | oxidation of 2-hydroxyestradiol | 0,0032 | CYP1A1 | 1 |
| E1 | Lipid metabolism | Endocrine System Development and Function, Lipid Metabolism, Small Molecule Biochemistry | reduction of dihydrotestosterone | 0,0032 | HSD3B2 | 1 |
| E1 | Lipid metabolism | Endocrine System Development and Function, Lipid Metabolism, Small Molecule Biochemistry, Vitamin and Mineral Metabolism | steroidogenesis of hormone | 0,00349 | CYP1A1,HSD3B2 | 2 |
| E1 | Lipid metabolism | Drug Metabolism, Endocrine System Development and Function, Lipid Metabolism, Small Molecule Biochemistry, Vitamin and Mineral Metabolism | metabolism of beta-estradiol | 0,00373 | CYP1A1 | 1 |
| E1 | Lipid metabolism | Lipid Metabolism, Small Molecule Biochemistry, Vitamin and Mineral Metabolism | conversion of vitamin A | 0,00427 | CYP1A1 | 1 |
| E1 | Lipid metabolism | Endocrine System Development and Function, Lipid Metabolism, Small Molecule Biochemistry | conversion of pregnenolone | 0,00533 | HSD3B2 | 1 |
| E2 | Development | Organismal Development | morphology of head | 6,8E-12 | ADAMTS2,ADORA2A,AHR,AKT3,APOB,ATXN1,BFSP1,CACNB3,CFTR,COL5A2,COL8A2,CPLX3,CYP1B1,CYP26A1,DHRS3,DNMT3A,DSCAM,ERBB4,FAM20C,FOLR1,FOXF2,FOXI1,FOXQ1,GCGR,GCM2,GDF6,GRIN1,GRIN2B,GSC,JAG2,MITF,NFIX,NOG,NTRK1,NTRK3,PARK2,PHC2,POSTN,PPP3CA,PRKG1,PTPRQ,PVALB,RARG,RDH10,RICTOR,SLC12A2,SLC32A1,SLC4A5,SLC6A4,SMPD3,SOSTDC1,TNR,TUB,WNT9B | 54 |
| E2 | Development | Organismal Development, Organismal Injury and Abnormalities | abnormal morphology of head | 3,23E-09 | ADAMTS2,ADORA2A,AHR,AKT3,APOB,BFSP1,CACNB3,CFTR,COL5A2,COL8A2,CPLX3,CYP1B1,CYP26A1,DHRS3,DSCAM,ERBB4,FAM20C,FOLR1,FOXF2,FOXI1,FOXQ1,GDF6,GRIN1,GRIN2B,GSC,JAG2,MITF,NFIX,NOG,NTRK3,PARK2,PHC2,POSTN,PTPRQ,PVALB,RARG,RDH10,SLC12A2,SLC32A1,SLC6A4,SMPD3,SOSTDC1,TNR,TUB,WNT9B | 45 |
| E2 | Development | Embryonic Development, Organismal Development | development of head | 5,64E-09 | ADORA2A,ANGPTL3,APOB,ATXN1,BFSP1,BMP2,BNC2,COL5A2,COL8A2,CPLX3,CYP1B1,CYP26A1,DHRS3,DNMT3A,DSCAM,ERBB4,FAM20C,FGF7,FOXD1,FOXF2,FOXI1,FOXQ1,GCGR,GDF6,GRIN1,GSC,JAG2,JAM3,MDGA1,MITF,NFIX,NKX2-3,NOG,NR1D1,NTRK1,PHC2,POSTN,PPARA,PRKG1,PTPRQ,RARG,RDH10,SLC23A1,SLC6A4,SOSTDC1,TRPM1,TUB,WNT9B | 48 |
| E2 | Development | Embryonic Development, Organismal Development | development of body axis | 7,08E-09 | ADORA2A,ANGPTL3,APOB,ATXN1,BFSP1,BMP2,BNC2,COL5A2,COL8A2,CPLX3,CYP1B1,CYP26A1,DHRS3,DNMT3A,DSCAM,ERBB4,FAM20C,FGF7,FOXD1,FOXF2,FOXI1,FOXQ1,GCGR,GDF6,GRIN1,GSC,HOXA4,HOXB5,JAG2,JAM3,MDGA1,MITF,NFIX,NKX2-3,NOG,NR1D1,NTRK1,PHC2,POSTN,PPARA,PRKG1,PTPRQ,RARG,RDH10,SLC23A1,SLC6A4,SOSTDC1,TRPM1,TUB,WNT9B | 50 |
| E2 | Development | Cardiovascular System Development and Function, Organ Morphology, Organismal Development | morphology of heart | 4,38E-08 | ACE,AHR,AKT3,APOB,ARG2,BMP10,CACNB3,CYP26A1,DHRS3,ERBB4,GJC1,HBEGF,LASP1,NLRP3,NOG,NPR1,NTRK3,POSTN,PPARA,PPARGC1B,PPP3CA,PRKG1,PTGER4,RARG,RGS19,RGS4,RGS6,SLC6A4,SLCO2A1 | 29 |
| E2 | Development | Organismal Development | morphology of body cavity | 4,83E-08 | ACE,AHR,AKT3,ANGPTL3,APOB,ARG2,BMP10,BMP2,CACNB3,CCBE1,CD151,CFTR,CYP1A1,CYP26A1,DHRS3,ERBB4,FGF7,FOLR1,FOXF2,GCGR,GJC1,GRIN1,HBEGF,JAG2,LASP1,LFNG,NFIX,NKX2-3,NLRP3,NOG,NPR1,NR3C2,NTRK3,P2RX3,PIK3CD,POSTN,PPARA,PPARGC1B,PPP3CA,PRKG1,PTGER4,RARG,RASGRF1,RDH10,RGS19,RGS4,RGS6,SGMS1,SLC12A2,SLC2A2,SLC6A4,SLC9A3,SLCO2A1,SMPD3,SSTR5,VIPR1,WNT9B,ZP2,ZP3 | 59 |
| E2 | Development | Organismal Development | size of body | 0,000000414 | ADRB3,AHR,AKT3,ANGPTL3,CFTR,COL5A2,DHRS3,DNMT3A,DPP4,FAM20C,GDF6,GPD2,GSC,HBEGF,HTR4,KCNC3,KCNMA1,LASP1,LFNG,LMTK3,NCOA3,NDRG1,NFIX,NTRK1,NTRK3,PARK2,POSTN,PPARGC1B,PPP3CA,PTGES,RARG,RASGRF1,SGMS1,SLC12A2,SLC2A2,SLC6A1,SMPD3,VIPR1,ZNF385A | 39 |
| E2 | Development | Cardiovascular System Development and Function, Organismal Development | angiogenesis | 0,000000803 | ADAMTS2,ADORA2A,AHR,AKT3,ANGPTL3,APOB,ATXN1,BMP10,BMP2,CCBE1,CD151,COL8A2,CYP1B1,DCBLD2,FGF7,GJC1,HBEGF,HOXB5,HTR4,JAM3,KCNMA1,NCOA3,NDRG1,NKX2-3,NOG,NPR1,PPARA,PPARGC1B,PRKG1,PTGER4,PTGES,PTK2B,RARG,RGS4,RGS5,RICTOR,SERPIND1,SLC9A3R2,SMPD3,TMEM100,TNMD,TRPC5 | 42 |
| E2 | Development | Embryonic Development, Organ Development, Organismal Development, Tissue Development | development of sensory organ | 0,00000264 | ADORA2A,ANGPTL3,APOB,BFSP1,BMP2,BNC2,COL5A2,COL8A2,CPLX3,CYP1B1,DNMT3A,DSCAM,FGF7,FOXD1,FOXI1,GCGR,GDF6,GRIN2B,GSC,JAM3,MITF,NOG,NTRK1,PTPRQ,RARG,RDH10,TRPM1,TUB | 28 |
| E2 | Development | Cardiovascular System Development and Function, Organ Morphology, Organismal Development | morphology of heart ventricle | 0,00000298 | APOB,ARG2,BMP10,DHRS3,ERBB4,HBEGF,NLRP3,NOG,NPR1,NTRK3,PPARGC1B,PTGER4,RARG,RGS19,SLC6A4,SLCO2A1 | 16 |
| E2 | Lipid metabolism | Lipid Metabolism, Molecular Transport, Small Molecule Biochemistry | concentration of lipid | 1,57E-09 | AADAC,ACE,ACSS1,ADORA2A,ADRB3,AHR,AKT3,ANGPTL3,APOB,AVPR1A,CETP,CFTR,CYP1A1,CYP1B1,CYP26A1,CYP3A7,DHRS3,FGF7,GCGR,GPD2,KCNMA1,MRAS,MRC1,NPR1,NTRK1,PCYT2,PLA1A,PPARA,PPARGC1B,PPP3CA,PRKG1,PTGDR2,PTGER4,PTGES,PVALB,RGS4,SGMS1,SLC12A2,SLC2A2,SLC4A5,SLC6A11,SLC6A4,SLC9A3,SLCO2A1,SMPD3,TACR1 | 46 |
| E2 | Lipid metabolism | Lipid Metabolism, Small Molecule Biochemistry, Vitamin and Mineral Metabolism | metabolism of retinoid | 0,000000249 | AHR,CYP1A1,CYP1B1,CYP26A1,CYP3A7,DHRS3,PLB1,RARG,RDH10 | 9 |
| E2 | Lipid metabolism | Lipid Metabolism, Small Molecule Biochemistry, Vitamin and Mineral Metabolism | metabolism of retinol | 0,00000349 | CYP1A1,CYP1B1,CYP26A1,DHRS3,PLB1,RDH10 | 6 |
| E2 | Lipid metabolism | Lipid Metabolism, Small Molecule Biochemistry | fatty acid metabolism | 0,00000354 | ABCG4,ACSS1,ADORA2A,ANGPTL3,APOB,AVPR1A,BMP2,CETP,CFTR,CYP1A1,CYP1B1,CYP46A1,ERBB4,MAPK10,MITF,PARK2,PLA1A,PMP2,PPARA,PTGES,PTK2B,PVALB,SAR1A,SGMS1,SLC6A1,SLC6A11,SLC9A3R2,SLCO1C1,SLCO2A1,SMPD3,TACR1 | 31 |
| E2 | Lipid metabolism | Lipid Metabolism, Molecular Transport, Small Molecule Biochemistry | concentration of fatty acid | 0,00000472 | ACSS1,ADORA2A,ADRB3,ANGPTL3,APOB,CFTR,CYP1A1,CYP1B1,FGF7,GPD2,PPARA,PPARGC1B,PTGDR2,PTGER4,PTGES,RGS4,SLC2A2,SLC6A11,SLCO2A1 | 19 |
| E2 | Lipid metabolism | Lipid Metabolism, Small Molecule Biochemistry, Vitamin and Mineral Metabolism | metabolism of terpenoid | 0,0000113 | AHR,ANGPTL3,APOB,BMP2,CETP,CFTR,CYP1A1,CYP1B1,CYP26A1,CYP3A7,CYP46A1,DHRS3,FGF7,KCNMA1,PLB1,RARG,RDH10,SQLE | 18 |
| E2 | Lipid metabolism | Lipid Metabolism, Molecular Transport, Small Molecule Biochemistry | transport of lipid | 0,000014 | ABCG4,ADORA2A,APOB,CETP,CFTR,CYP46A1,PARK2,PLA1A,PMP2,PPARA,SAR1A,SLC6A1,SLC6A11,SLC9A3R2,SLCO1C1,SLCO2A1 | 16 |
| E2 | Lipid metabolism | Drug Metabolism, Lipid Metabolism, Small Molecule Biochemistry, Vitamin and Mineral Metabolism | metabolism of tretinoin | 0,0000141 | AHR,CYP1A1,CYP26A1,CYP3A7,RARG,RDH10 | 6 |
| E2 | Lipid metabolism | Lipid Metabolism, Small Molecule Biochemistry | conversion of lipid | 0,0000144 | ANGPTL3,APOB,CETP,CFTR,CYP1A1,CYP1B1,CYP3A7,CYP46A1,GSTP1,PARK2,PPARA,PTGER4,PTGES,RDH10 | 14 |
| E2 | Lipid metabolism | Lipid Metabolism, Molecular Transport, Small Molecule Biochemistry | quantity of steroid hormone | 0,0000636 | ACE,AHR,AVPR1A,CETP,CYP3A7,KCNMA1,PVALB,SLC4A5,SLC6A4,SLC9A3,TACR1 | 11 |
| E2 | Molecular transport | Molecular Transport | transport of molecule | 2,46E-20 | ABCG4,ACE,ADORA2A,AHCYL1,AKT3,ANO8,APOB,AQP4,ATP1B1,ATP2B3,AVPR1A,BMP2,BTN2A2,CACNB3,CETP,CFTR,CPLX3,CYP46A1,DPP6,ERBB4,FGF7,FGG,FOLR1,GCGR,GRIK1,GRIN1,GRIN2B,HCN4,HK1,KCNC3,KCNIP3,KCNJ1,KCNJ15,KCNJ6,KCNK18,KCNMA1,LASP1,MGEA5,NMB,NR3C2,NTRK1,P2RX3,PARK2,PIEZO2,PLA1A,PLCB3,PLIN3,PMP2,PPARA,PPARGC1B,PPP3CA,PRKAG3,PRKCZ,PRKG1,PTGER4,PTK2B,RASGRF1,RHAG,RICTOR,SAR1A,SCN4B,SGMS1,SLC12A2,SLC15A1,SLC22A13,SLC23A1,SLC24A2,SLC25A20,SLC25A5,SLC26A6,SLC28A2,SLC2A2,SLC2A3,SLC32A1,SLC39A4,SLC41A1,SLC4A5,SLC6A1,SLC6A11,SLC6A4,SLC9A3,SLC9A3R2,SLCO1C1,SLCO2A1,SMPD3,STXBP5L,TACR1,TNR,TRPC5,TRPM1,TRPV6,ZP2,ZP3 | 93 |
| E2 | Molecular transport | Molecular Transport | transport of ion | 6,82E-12 | AHCYL1,AKT3,ANO8,ATP1B1,CACNB3,CFTR,DPP6,GRIN1,GRIN2B,HCN4,KCNIP3,KCNJ1,KCNJ15,KCNJ6,KCNK18,KCNMA1,LASP1,NR3C2,P2RX3,PIEZO2,PLCB3,PPP3CA,RHAG,SCN4B,SLC12A2,SLC23A1,SLC24A2,SLC25A20,SLC26A6,SLC39A4,SLC41A1,SLC4A5,SLC9A3,TRPC5,TRPV6 | 35 |
| E2 | Molecular transport | Molecular Transport | transport of cation | 2,29E-11 | AHCYL1,AKT3,ATP1B1,CACNB3,DPP6,GRIN1,GRIN2B,HCN4,KCNIP3,KCNJ1,KCNJ15,KCNJ6,KCNK18,KCNMA1,NR3C2,P2RX3,PIEZO2,PLCB3,PPP3CA,RHAG,SCN4B,SLC12A2,SLC23A1,SLC24A2,SLC25A20,SLC39A4,SLC41A1,SLC9A3,TRPC5,TRPV6 | 30 |
| E2 | Molecular transport | Molecular Transport | transport of metal ion | 5,68E-10 | AHCYL1,AKT3,ATP1B1,CACNB3,DPP6,GRIN1,GRIN2B,KCNIP3,KCNJ1,KCNJ15,KCNJ6,KCNK18,KCNMA1,NR3C2,PLCB3,PPP3CA,SCN4B,SLC12A2,SLC23A1,SLC24A2,SLC39A4,SLC41A1,SLC9A3,TRPC5,TRPV6 | 25 |
| E2 | Molecular transport | Molecular Transport | transport of inorganic cation | 8,81E-10 | AHCYL1,AKT3,ATP1B1,CACNB3,DPP6,GRIN1,GRIN2B,KCNIP3,KCNJ1,KCNJ15,KCNJ6,KCNK18,KCNMA1,NR3C2,PLCB3,PPP3CA,RHAG,SCN4B,SLC12A2,SLC23A1,SLC24A2,SLC39A4,SLC41A1,SLC9A3,TRPC5,TRPV6 | 26 |
| E2 | Molecular transport | Lipid Metabolism, Molecular Transport, Small Molecule Biochemistry | concentration of lipid | 1,57E-09 | AADAC,ACE,ACSS1,ADORA2A,ADRB3,AHR,AKT3,ANGPTL3,APOB,AVPR1A,CETP,CFTR,CYP1A1,CYP1B1,CYP26A1,CYP3A7,DHRS3,FGF7,GCGR,GPD2,KCNMA1,MRAS,MRC1,NPR1,NTRK1,PCYT2,PLA1A,PPARA,PPARGC1B,PPP3CA,PRKG1,PTGDR2,PTGER4,PTGES,PVALB,RGS4,SGMS1,SLC12A2,SLC2A2,SLC4A5,SLC6A11,SLC6A4,SLC9A3,SLCO2A1,SMPD3,TACR1 | 46 |
| E2 | Molecular transport | Molecular Transport | transport of metal | 9,1E-09 | AHCYL1,AKT3,ATP1B1,BMP2,CACNB3,DPP6,GRIN1,GRIN2B,KCNIP3,KCNJ1,KCNJ15,KCNJ6,KCNK18,KCNMA1,NR3C2,PLCB3,PPP3CA,RHAG,SCN4B,SLC12A2,SLC23A1,SLC24A2,SLC39A4,SLC41A1,SLC9A3,TRPC5,TRPV6 | 27 |
| E2 | Molecular transport | Molecular Transport | secretion of molecule | 0,000000032 | ACE,ADORA2A,AKT3,APOB,AVPR1A,BMP2,BTN2A2,CFTR,CPLX3,FGF7,FGG,GCGR,GRIK1,GRIN2B,KCNC3,KCNJ1,NMB,NTRK1,PARK2,PPARA,PRKCZ,PRKG1,PTGER4,PTK2B,RASGRF1,RICTOR,SGMS1,SLC12A2,SLC26A6,SLC6A1,SMPD3,STXBP5L,TACR1 | 33 |
| E2 | Molecular transport | Molecular Transport | transport of monovalent inorganic cation | 0,000000585 | AHCYL1,AKT3,ATP1B1,DPP6,KCNIP3,KCNJ1,KCNJ15,KCNJ6,KCNK18,KCNMA1,NR3C2,RHAG,SCN4B,SLC12A2,SLC23A1,SLC9A3 | 16 |
| E2 | Molecular transport | Lipid Metabolism, Molecular Transport, Small Molecule Biochemistry | concentration of fatty acid | 0,00000472 | ACSS1,ADORA2A,ADRB3,ANGPTL3,APOB,CFTR,CYP1A1,CYP1B1,FGF7,GPD2,PPARA,PPARGC1B,PTGDR2,PTGER4,PTGES,RGS4,SLC2A2,SLC6A11,SLCO2A1 | 19 |
| E3 | Development | Organismal Development | morphology of body cavity | 2,27E-15 | ABCB11,ABCB4,ABCC2,ABCG2,ACE,ADRB2,ARG2,BMPER,CACNA1H,CCBE1,CCKBR,CD38,CDH11,CFTR,CHST3,CISH,CLU,COL10A1,CPEB1,CSF1,CSF1R,CTGF,CTHRC1,CXCR4,CYP1A1,CYP51A1,DHCR7,DHRS3,DLX3,EDN1,EPOR,F9,FADS2,FAH,FDFT1,FGF7,FGL1,FMN1,FMOD,FOS,FZD9,GLIS2,GSTZ1,HSD17B7,HSP90B1,IGFBP3,IGKC,INPP5D,KISS1R,LBX1,LUM,MAP3K14,MFSD2A,MLXIPL,MOV10L1,MYH11,NEUROD1,NFATC1,NFATC4,NFIX,NGFR,NKX2-5,NODAL,NUPR1,OSR1,PDC,PGLYRP2,PNPLA2,POSTN,PRDM1,PRDX1,PTX3,RAG1,RARG,RBP4,RUNX3,SC5D,SELE,SLC12A2,SLC14A2,SLC20A1,SLC22A4,SLC2A9,SLC9A3,SMTN,SPDEF,SSTR5,STEAP4,TCF21,TGFB3,TPH1,TRAF3IP2,UNCX,VIPR1,ZP2 | 95 |
| E3 | Development | Organismal Development | abnormal morphology of body cavity | 1,62E-14 | ABCB11,ABCB4,ABCG2,ACE,BMPER,CACNA1H,CCBE1,CCKBR,CD38,CFTR,CHST3,CISH,CLU,COL10A1,CPEB1,CSF1,CSF1R,CTGF,CTHRC1,CXCR4,CYP1A1,CYP51A1,DHCR7,DHRS3,DLX3,EDN1,EPOR,F9,FADS2,FAH,FDFT1,FGF7,FMN1,FMOD,FOS,FZD9,GLIS2,GSTZ1,HSD17B7,HSP90B1,IGFBP3,IGKC,INPP5D,KISS1R,LUM,MAP3K14,MFSD2A,MYH11,NEUROD1,NFATC1,NFATC4,NFIX,NGFR,NKX2-5,NODAL,NUPR1,OSR1,PGLYRP2,PNPLA2,POSTN,PRDM1,PRDX1,PTX3,RAG1,RARG,RBP4,SC5D,SELE,SLC12A2,SLC20A1,SLC22A4,SLC2A9,SLC9A3,SMTN,SPDEF,SSTR5,TCF21,TGFB3,TRAF3IP2,UNCX,VIPR1,ZP2 | 82 |
| E3 | Development | Organismal Development, Organismal Injury and Abnormalities | abnormal morphology of abdomen | 1,59E-13 | ABCB11,ABCB4,ABCG2,ACE,AVPR1A,BMPER,CCBE1,CCKBR,CD38,CFTR,CHST3,CISH,CLU,COL10A1,CPEB1,CSF1,CSF1R,CTHRC1,CYP11B1,CYP1A1,DHCR7,EPOR,F9,FADS2,FAH,FDFT1,FGF7,FMN1,FOS,FZD9,GLIS2,GSTZ1,IGFBP3,IGKC,INPP5D,KISS1R,MAP3K14,MFSD2A,MYH11,NEUROD1,NFATC4,NFIX,NGFR,NODAL,NUPR1,PGLYRP2,PRDM1,PRDX1,PTX3,RAG1,RARG,SC5D,SELE,SLC12A2,SLC20A1,SLC22A4,SLC2A9,SLC9A3,SMTN,SPDEF,SSTR5,TCF21,TGFB3,TRAF3IP2,VIPR1,ZP2 | 66 |
| E3 | Development | Embryonic Development, Organismal Development | development of body axis | 8,39E-13 | ADRB2,AIPL1,ASCL1,BMPER,CACNA1F,CDX1,CHST3,CLCN2,COL10A1,COL5A2,COL8A2,CRISPLD2,CRTAC1,CSF1,CSF1R,CTHRC1,CXCL8,CXCR4,CYP1B1,DHCR7,DHRS3,DLL1,DLX3,DNMT3A,DSP,EDN1,EPOR,FAIM2,FGF7,FMOD,FOS,FOXC1,FOXI1,FOXJ1,FOXQ1,FRZB,FZD9,GDF3,GSC,HES3,HOXB6,HOXC5,HSD17B7,HSP90B1,INSIG2,LMO7,LUM,MCOLN3,MITF,NEUROD1,NFIX,NGFR,NKX2-5,NODAL,PDE6C,POSTN,PRDM1,PRPH2,RAG1,RARG,RBP4,RDH12,RDH8,RHO,RPE65,SLC17A7,SOSTDC1,SOX8,STRA6,TCF21,TGFB3,TIPARP,USH1G | 73 |
| E3 | Development | Embryonic Development, Organismal Development | development of head | 1,96E-12 | ADRB2,AIPL1,ASCL1,BMPER,CACNA1F,CHST3,CLCN2,COL10A1,COL5A2,COL8A2,CRISPLD2,CRTAC1,CSF1,CSF1R,CTHRC1,CXCL8,CXCR4,CYP1B1,DHCR7,DHRS3,DLL1,DLX3,DNMT3A,DSP,EDN1,EPOR,FAIM2,FGF7,FMOD,FOS,FOXC1,FOXI1,FOXJ1,FOXQ1,FRZB,FZD9,GDF3,GSC,HES3,HSD17B7,INSIG2,LMO7,LUM,MCOLN3,MITF,NEUROD1,NFIX,NGFR,NKX2-5,NODAL,PDE6C,POSTN,PRDM1,PRPH2,RAG1,RARG,RBP4,RDH12,RDH8,RHO,RPE65,SLC17A7,SOSTDC1,SOX8,STRA6,TCF21,TGFB3,TIPARP,USH1G | 69 |
| E3 | Development | Embryonic Development, Organismal Development | development of body trunk | 6,78E-12 | AACS,ACE,ADRB2,ANGPT1,ASCL1,BMPER,CCBE1,CDH11,CDX1,CELA1,CFTR,CISH,CLU,CPEB1,CSF1,CSF1R,CTGF,CXCR4,CYP11B1,CYP51A1,DHCR7,DHRS3,DLL1,DSP,EDN1,EPOR,F5,FADS2,FGF7,FMN1,FOS,FOXC1,FOXJ1,GDF3,GLIS2,GSC,GSTZ1,HOXC5,INPP5D,KCNJ1,KISS1R,LBX1,MAP3K14,MOV10L1,MYH11,MYOCD,NEK1,NEURL1,NEUROD1,NFATC1,NFATC4,NFIX,NGFR,NKX2-5,NODAL,OSR1,PNPLA2,PRDM1,RAG1,RARG,RBM20,RBP4,RICTOR,SELE,SLC14A2,SLC20A1,SLC22A5,SMTN,SOCS5,SOX8,SREBF1,STRA6,TCF21,TGFB3,TIPARP,TRAF3IP2,UNCX,ZP2 | 78 |
| E3 | Development | Organismal Development | morphology of head | 1,64E-11 | ACHE,ADRB2,AIPL1,ASCL1,BMPER,CACNA1F,CBLN1,CDX1,CFTR,CLCN2,COL5A2,COL8A2,COL9A1,CRTAC1,CSF1,CSF1R,CTGF,CXCR4,CYP1B1,CYP51A1,DDIT3,DHCR7,DHRS3,DNMT3A,EDN1,FAIM2,FMOD,FOS,FOXC1,FOXI1,FOXJ1,FOXQ1,FZD9,GAL3ST1,GCM2,GSC,INSIG2,KCNJ2,LBX1,LMO7,LRRTM1,LUM,MITF,NEUROD1,NFATC4,NFIX,NGFR,POSTN,PPP1R9B,PRPH2,PVALB,RAG1,RARG,RBP4,RDH12,RDH8,RHO,RICTOR,RPE65,SC5D,SLC12A2,SOSTDC1,SPAG17,STRA6,TGFB3,TRAF3IP2,USH1G | 67 |
| E3 | Development | Organismal Development, Organismal Injury and Abnormalities | abnormal morphology of head | 1,37E-10 | ACHE,ADRB2,AIPL1,ASCL1,BMPER,CACNA1F,CBLN1,CDX1,CFTR,CLCN2,COL5A2,COL8A2,COL9A1,CRTAC1,CSF1,CSF1R,CTGF,CXCR4,CYP1B1,CYP51A1,DHCR7,DHRS3,EDN1,FMOD,FOS,FOXC1,FOXI1,FOXJ1,FOXQ1,FZD9,GAL3ST1,GSC,INSIG2,KCNJ2,LMO7,LRRTM1,LUM,MITF,NEUROD1,NFATC4,NFIX,NGFR,POSTN,PPP1R9B,PVALB,RAG1,RARG,RBP4,RDH12,RDH8,RHO,RPE65,SC5D,SLC12A2,SOSTDC1,SPAG17,STRA6,TGFB3,TRAF3IP2,USH1G | 60 |
| E3 | Development | Embryonic Development, Organ Development, Organismal Development, Tissue Development | development of sensory organ | 1,94E-10 | ADRB2,AIPL1,ASCL1,BMPER,CACNA1F,CLCN2,COL5A2,COL8A2,CTHRC1,CXCL8,CYP1B1,DLL1,DNMT3A,EDN1,FGF7,FMOD,FOS,FOXC1,FOXI1,FRZB,GDF3,GSC,INSIG2,LMO7,LUM,MCOLN3,MITF,NEUROD1,NGFR,NKX2-5,PDE6C,PRDM1,PRPH2,RAG1,RARG,RBP4,RDH12,RDH8,RHO,RPE65,SOX8,STRA6,TGFB3,USH1G | 44 |
| E3 | Development | Organismal Development | size of body | 2,92E-09 | ABCB11,ACHE,ADRB2,BMPER,CACNA1H,CBX7,CFTR,COL10A1,COL5A2,CSF1,CSF1R,CXCR4,CYP11B1,DDB2,DHRS3,DNMT3A,DPP4,FADS2,FGL1,FMOD,FOS,FOXJ1,FZD9,GFRA2,GPSM1,GSC,GSTZ1,IGFALS,IGFBP3,INPP5D,KISS1R,LMO7,LUM,MFSD2A,MYH11,MYH4,NEUROD1,NFIX,NGFR,NODAL,POSTN,PPP1R9B,RARG,RNF213,RUNX3,SLC10A2,SLC12A2,SLC14A2,SLC17A7,SLC2A9,SLC5A3,STC2,TPH1,VIPR1,VIPR2 | 55 |
| E3 | Lipid metabolism | Lipid Metabolism, Small Molecule Biochemistry, Vitamin and Mineral Metabolism | metabolism of terpenoid | 2,82E-22 | AACS,ABCG5,APOA4,BCO2,CACNA1H,CETP,CFTR,CXCL8,CYP11B1,CYP1A1,CYP1B1,CYP3A7,CYP51A1,DHCR24,DHCR7,DHRS3,EBP,EDN1,FDFT1,FDPS,FGF7,FGL1,GREM2,HMGCR,HSD17B1,HSD17B7,IDI1,INSIG2,LSS,MSMO1,MVK,NSDHL,PLB1,RARG,RBP4,RDH12,RDH8,RETSAT,RLBP1,RPE65,SLC10A2,SQLE,SREBF1,SULT1A1,SULT2B1,TIPARP | 46 |
| E3 | Lipid metabolism | Lipid Metabolism, Small Molecule Biochemistry, Vitamin and Mineral Metabolism | steroid metabolism | 1,27E-16 | AACS,ABCG5,APOA4,CACNA1H,CETP,CFTR,CXCL8,CYP11B1,CYP1A1,CYP1B1,CYP3A7,CYP51A1,DHCR24,DHCR7,EBP,EDN1,FDFT1,FDPS,FGF7,FGL1,GREM2,HMGCR,HSD17B1,HSD17B7,IDI1,INSIG2,LSS,MSMO1,MVK,NSDHL,SLC10A2,SQLE,SREBF1,SULT1A1,SULT2B1,TIPARP | 36 |
| E3 | Lipid metabolism | Lipid Metabolism, Molecular Transport, Small Molecule Biochemistry | concentration of lipid | 1,14E-13 | ABCB11,ABCB4,ABCC2,ABCG2,ABCG5,ACE,ACSS1,ADRB2,ALPI,APOA4,AQP8,AQP9,AVPR1A,CD38,CERS2,CETP,CFTR,CLU,CRY2,CSF1,CXCL8,CYP11B1,CYP1A1,CYP1B1,CYP3A7,DHCR24,DHCR7,DHRS3,DIO1,EDN1,FADS2,FDFT1,FGF7,FGL1,FOS,GAL3ST1,GFRA2,HMGCR,HRH3,HSD17B1,IL1R2,INPP5D,KCNA6,KISS1R,LIPG,MFSD2A,MLXIPL,MMP8,MRC1,NGFR,NME4,P2RY2,PNPLA2,PNPLA5,PVALB,RBP4,RDH12,RDH8,RPE65,SC5D,SLC10A2,SLC12A2,SLC9A3,SREBF1,STEAP4,STRA6,SULT2B1 | 67 |
| E3 | Lipid metabolism | Lipid Metabolism, Small Molecule Biochemistry, Vitamin and Mineral Metabolism | metabolism of cholesterol | 4,17E-13 | ABCG5,APOA4,CETP,CFTR,CYP51A1,DHCR24,DHCR7,EBP,FDFT1,FDPS,FGL1,HMGCR,HSD17B7,IDI1,INSIG2,LSS,MVK,NSDHL,SQLE,SREBF1 | 20 |
| E3 | Lipid metabolism | Lipid Metabolism, Small Molecule Biochemistry, Vitamin and Mineral Metabolism | synthesis of cholesterol | 8,53E-13 | ABCG5,CETP,CFTR,CYP51A1,DHCR24,DHCR7,EBP,FDFT1,FDPS,HMGCR,HSD17B7,IDI1,INSIG2,LSS,MVK,SREBF1 | 16 |
| E3 | Lipid metabolism | Lipid Metabolism, Small Molecule Biochemistry, Vitamin and Mineral Metabolism | synthesis of steroid | 2,13E-12 | ABCG5,ANGPT1,CACNA1H,CETP,CFTR,CSF1,CXCL8,CYP11B1,CYP1A1,CYP1B1,CYP51A1,DHCR24,DHCR7,EBP,EDN1,FDFT1,FDPS,FGF7,GREM2,HMGCR,HSD17B1,HSD17B3,HSD17B7,IDI1,INSIG2,LSS,MVK,PDE5A,QRFPR,RDH8,SLC9A3R2,SREBF1 | 32 |
| E3 | Lipid metabolism | Lipid Metabolism, Small Molecule Biochemistry, Vitamin and Mineral Metabolism | synthesis of terpenoid | 6,06E-12 | ABCG5,ANGPT1,CACNA1H,CETP,CFTR,CSF1,CXCL8,CYP11B1,CYP1A1,CYP1B1,CYP51A1,DHCR24,DHCR7,EBP,EDN1,FDFT1,FDPS,FGF7,GREM2,HMGCR,HSD17B1,HSD17B3,HSD17B7,IDI1,INSIG2,LSS,MVD,MVK,PDE5A,QRFPR,RDH8,SLC9A3R2,SREBF1 | 33 |
| E3 | Lipid metabolism | Lipid Metabolism, Small Molecule Biochemistry | synthesis of lipid | 9,29E-11 | ABCG5,ACSS1,ACSS2,ADRB2,ANGPT1,APOA4,AVPR1A,CACNA1H,CD82,CERS2,CETP,CFTR,CLU,CSF1,CXCL8,CYP11B1,CYP1A1,CYP1B1,CYP51A1,DHCR24,DHCR7,DPP4,EBP,EDN1,FADS2,FAM213B,FDFT1,FDPS,FGF7,FOS,GAL3ST1,GREM2,HMGCR,HSD17B1,HSD17B3,HSD17B7,IDI1,IL1R2,INPP5D,INSIG2,LSS,MAP3K14,MITF,MLXIPL,MVD,MVK,NGFR,PDE5A,PNPLA2,PTX3,PVALB,QRFPR,RDH8,RLBP1,SLC9A3R2,SREBF1 | 56 |
| E3 | Lipid metabolism | Lipid Metabolism, Small Molecule Biochemistry, Vitamin and Mineral Metabolism | metabolism of retinol | 2,31E-10 | CYP1A1,CYP1B1,DHRS3,PLB1,RBP4,RDH12,RDH8,RETSAT,RLBP1,RPE65 | 10 |
| E3 | Lipid metabolism | Lipid Metabolism, Small Molecule Biochemistry | metabolism of membrane lipid derivative | 2,16E-09 | ABCG5,ADRB2,APOA4,CD82,CERS2,CETP,CFTR,CXCL8,CYP51A1,DHCR24,DHCR7,EBP,EDN1,FDFT1,FDPS,FGF7,FGL1,FOS,GAL3ST1,HMGCR,HSD17B7,IDI1,INPP5D,INSIG2,LSS,MVK,NGFR,NSDHL,PLB1,PLCD4,PTX3,SQLE,SREBF1,ST6GALNAC3 | 34 |
| E3 | Molecular transport | Lipid Metabolism, Molecular Transport, Small Molecule Biochemistry | concentration of lipid | 1,14E-13 | ABCB11,ABCB4,ABCC2,ABCG2,ABCG5,ACE,ACSS1,ADRB2,ALPI,APOA4,AQP8,AQP9,AVPR1A,CD38,CERS2,CETP,CFTR,CLU,CRY2,CSF1,CXCL8,CYP11B1,CYP1A1,CYP1B1,CYP3A7,DHCR24,DHCR7,DHRS3,DIO1,EDN1,FADS2,FDFT1,FGF7,FGL1,FOS,GAL3ST1,GFRA2,HMGCR,HRH3,HSD17B1,IL1R2,INPP5D,KCNA6,KISS1R,LIPG,MFSD2A,MLXIPL,MMP8,MRC1,NGFR,NME4,P2RY2,PNPLA2,PNPLA5,PVALB,RBP4,RDH12,RDH8,RPE65,SC5D,SLC10A2,SLC12A2,SLC9A3,SREBF1,STEAP4,STRA6,SULT2B1 | 67 |
| E3 | Molecular transport | Molecular Transport | transport of molecule | 7,23E-11 | ABCB11,ABCB4,ABCC2,ABCG2,ABCG5,ACE,ACHE,ADRA2C,ADRB2,AHCYL1,ANGPT1,APOA4,AQP4,AQP8,AQP9,ARF5,AVPR1A,BEST2,BMPER,CACNA1H,CAMK2A,CBLN1,CCKBR,CETP,CFTR,CHCHD4,CLCN2,CLIC5,CLU,CPEB1,CRY2,CXCL8,DOC2B,DRD4,EDN1,ENPP1,FGF7,FTMT,GABRA6,GALR1,GLUL,HRH3,IRF3,KCNA6,KCNIP2,KCNJ1,KCNJ15,KCNJ2,KCNQ5,KCNT1,KISS1R,LIPG,LSS,MFSD2A,NEUROD1,NFATC1,NGFR,NKX2-5,NMB,P2RY2,PIEZO2,PMP2,PNPLA2,RACGAP1,RAMP1,RBP4,RHAG,RICTOR,SCN1B,SLC10A2,SLC12A2,SLC13A2,SLC14A2,SLC16A3,SLC17A7,SLC20A1,SLC22A4,SLC22A5,SLC22A7,SLC25A42,SLC28A1,SLC2A9,SLC47A1,SLC5A3,SLC5A5,SLC6A13,SLC6A19,SLC9A3,SLC9A3R2,SREBF1,STEAP4,STRA6,TACR2,TGFB3,ZP2 | 95 |
| E3 | Molecular transport | Molecular Transport | transport of ion | 9,04E-09 | ABCC2,ADRB2,AHCYL1,AQP9,BEST2,CACNA1H,CAMK2A,CFTR,CLCN2,CLIC5,EDN1,KCNA6,KCNIP2,KCNJ1,KCNJ15,KCNJ2,KCNQ5,KCNT1,NFATC1,NKX2-5,PIEZO2,RACGAP1,RAMP1,RHAG,SCN1B,SLC10A2,SLC12A2,SLC17A7,SLC20A1,SLC22A4,SLC22A5,SLC22A7,SLC47A1,SLC5A5,SLC9A3,STEAP4,TACR2 | 37 |
| E3 | Molecular transport | Lipid Metabolism, Molecular Transport, Small Molecule Biochemistry | quantity of steroid | 4,22E-08 | ABCB11,ABCB4,ABCC2,ABCG5,ACE,ADRB2,APOA4,AQP8,AVPR1A,CETP,CLU,CRY2,CSF1,CYP11B1,CYP3A7,DHCR24,DHCR7,DIO1,EDN1,FDFT1,FGL1,HMGCR,HSD17B1,KISS1R,LIPG,MLXIPL,MRC1,P2RY2,PNPLA2,PVALB,SC5D,SLC10A2,SLC12A2,SLC9A3,SREBF1,STEAP4,SULT2B1 | 37 |
| E3 | Molecular transport | Lipid Metabolism, Molecular Transport, Small Molecule Biochemistry | transport of lipid | 0,00000246 | ABCB11,ABCB4,ABCC2,ABCG2,ABCG5,APOA4,AQP8,AQP9,CETP,CFTR,CLU,LIPG,LSS,MFSD2A,PMP2,RBP4,SLC10A2,SLC6A13,SLC9A3R2,SREBF1,STRA6 | 21 |
| E3 | Molecular transport | Lipid Metabolism, Molecular Transport, Small Molecule Biochemistry | secretion of lipid | 0,00000261 | ABCB11,ABCB4,ABCC2,ABCG5,ACE,APOA4,AVPR1A,CXCL8,DRD4,EDN1,FGF7,GALR1,HRH3,KISS1R,NMB,P2RY2,PNPLA2,RICTOR,SREBF1 | 19 |
| E3 | Molecular transport | Molecular Transport | transport of water | 0,00000312 | AQP4,AQP8,AQP9,CFTR,SLC12A2,SLC14A2 | 6 |
| E3 | Molecular transport | Molecular Transport | transport of anion | 0,00000395 | ABCC2,AHCYL1,AQP9,BEST2,CFTR,CLCN2,CLIC5,EDN1,RACGAP1,SLC12A2,SLC17A7,SLC20A1,SLC22A7,SLC5A5 | 14 |
| E3 | Molecular transport | Molecular Transport | export of molecule | 0,00000403 | ABCB11,ABCB4,ABCC2,ABCG2,ABCG5,AHCYL1,APOA4,CAMK2A,CETP,CFTR,CHCHD4,CLIC5,CLU,EDN1,LIPG,LSS,RHAG,SLC10A2,SLC12A2,SLC22A7,SLC47A1,SLC6A13,SLC9A3,SREBF1 | 24 |
| E3 | Molecular transport | Molecular Transport | transport of inorganic anion | 0,00000775 | ABCC2,AQP9,BEST2,CFTR,CLCN2,CLIC5,EDN1,RACGAP1,SLC12A2,SLC17A7,SLC20A1,SLC5A5 | 12 |
| E4 | Development | Embryonic Development, Organ Development, Organismal Development, Tissue Development | development of sensory organ | 8,56E-16 | AIPL1,ALDH1A3,ATOH7,BMPER,CACNA1F,CDKN2A,CHRNA10,CNGA3,Cngb1,CNGB3,COL5A2,COL8A2,CTHRC1,CXCL8,CYP1B1,EDN1,FAT3,FGF10,FGF7,FGFR3,FMOD,FOS,FOXC1,FOXI1,FOXL2,FRZB,GGT1,GNAT2,GNGT1,GUCA1A,GUCY2D,HMX3,INSIG2,LMX1B,MCOLN3,NKX2-5,NRTN,PAX5,PDE6C,PDE6G,PER2,PRPH2,RBP3,RBP4,RDH12,RDH8,RGS11,RHO,RP1L1,RPE65,SAG,STRA6,TSHR,VCAM1,WT1 | 55 |
| E4 | Development | Embryonic Development, Organismal Development | development of head | 1,59E-14 | AIPL1,ALDH1A3,ASPN,ATOH7,BMPER,CACNA1F,CDKN2A,CHRNA10,CLDN5,CNGA3,Cngb1,CNGB3,COL10A1,COL5A2,COL8A2,CRTAC1,CTHRC1,CXCL12,CXCL8,CXCR4,CYP1B1,DHCR7,EDN1,ESR2,FAIM2,FAT3,FGF10,FGF7,FGFR3,FMOD,FOS,FOXC1,FOXI1,FOXL2,FOXQ1,FRZB,GGT1,GNAT2,GNGT1,GRM5,GUCA1A,GUCY2D,HMX3,HSD17B7,INSIG2,LHX8,LMX1B,LOXL3,MCOLN3,NKX2-5,NKX3-1,NRTN,PAX5,PDE6C,PDE6G,PER2,POSTN,PRPH2,PTF1A,RBP3,RBP4,RDH12,RDH8,RGS11,RHO,RP1L1,RPE65,SAG,SEMA4C,SLC23A1,SOSTDC1,STRA6,TSHR,VCAM1,WNT8A,WT1 | 76 |
| E4 | Development | Embryonic Development, Organismal Development | development of body axis | 2,57E-14 | AIPL1,ALDH1A3,ASPN,ATOH7,BMPER,CACNA1F,CDKN2A,CHRNA10,CLDN5,CNGA3,Cngb1,CNGB3,COL10A1,COL5A2,COL8A2,CRTAC1,CTHRC1,CXCL12,CXCL8,CXCR4,CYP1B1,DHCR7,EDN1,ESR2,FAIM2,FAT3,FGF10,FGF7,FGFR3,FMOD,FOS,FOXC1,FOXI1,FOXL2,FOXQ1,FRZB,GGT1,GNAT2,GNGT1,GRM5,GUCA1A,GUCY2D,HMX3,HOXB6,HOXC5,HSD17B7,HSP90B1,INSIG2,LHX8,LMX1B,LOXL3,MCOLN3,NKX2-5,NKX3-1,NRTN,PAX5,PDE6C,PDE6G,PER2,POSTN,PRPH2,PTF1A,RBP3,RBP4,RDH12,RDH8,RGS11,RHO,RP1L1,RPE65,SAG,SEMA4C,SLC23A1,SOSTDC1,STRA6,TSHR,VCAM1,WNT8A,WT1 | 79 |
| E4 | Development | Embryonic Development, Ophthalmic Disease, Organ Development, Organ Morphology, Organismal Development, Organismal Injury and Abnormalities, Tissue Development, Visual System Development and Function | abnormal morphology of eye | 2,97E-13 | AIPL1,ALDH1A3,CACNA1F,CDKN2A,CNGA3,Cngb1,CNGB3,COL5A2,COL8A2,CYP1B1,FAT3,FMOD,FOS,FOXC1,FOXL2,GGT1,GUCA1A,GUCY2D,LMX1B,NRTN,PDE6G,PER2,RBP3,RBP4,RDH12,RDH8,RGS11,RHO,RP1L1,RPE65,SAG,STRA6 | 32 |
| E4 | Development | Cell Morphology, Embryonic Development, Organ Development, Organ Morphology, Organismal Development, Tissue Development, Tissue Morphology, Visual System Development and Function | morphology of photoreceptors | 6,15E-13 | AIPL1,CACNA1F,CDKN2A,CNGA3,Cngb1,CNGB3,GUCA1A,GUCY2D,PDE6G,PRPH2,RBP3,RDH12,RDH8,RHO,RP1L1,RPE65,SAG,STRA6 | 18 |
| E4 | Development | Cell Morphology, Embryonic Development, Ophthalmic Disease, Organ Development, Organ Morphology, Organismal Development, Organismal Injury and Abnormalities, Tissue Development, Tissue Morphology, Visual System Development and Function | abnormal morphology of photoreceptors | 1,02E-12 | AIPL1,CACNA1F,CDKN2A,CNGA3,Cngb1,CNGB3,GUCA1A,GUCY2D,PDE6G,RBP3,RDH12,RDH8,RHO,RP1L1,RPE65,SAG,STRA6 | 17 |
| E4 | Development | Embryonic Development, Organ Development, Organismal Development, Tissue Development, Visual System Development and Function | formation of eye | 1,11E-12 | AIPL1,ALDH1A3,ATOH7,CACNA1F,CDKN2A,CNGA3,Cngb1,CNGB3,COL5A2,COL8A2,CXCL8,CYP1B1,FAT3,FGF10,FGF7,FMOD,FOS,FOXC1,FOXL2,GGT1,GNAT2,GNGT1,GUCA1A,GUCY2D,LMX1B,NRTN,PAX5,PDE6C,PDE6G,PER2,PRPH2,RBP3,RBP4,RDH12,RDH8,RGS11,RHO,RP1L1,RPE65,SAG,STRA6,VCAM1,WT1 | 43 |
| E4 | Development | Embryonic Development, Organ Development, Organ Morphology, Organismal Development, Tissue Development, Visual System Development and Function | morphology of eye | 1,21E-12 | AIPL1,ALDH1A3,CACNA1F,CDKN2A,CNGA3,Cngb1,CNGB3,COL5A2,COL8A2,CYP1B1,FAT3,FMOD,FOS,FOXC1,FOXL2,GGT1,GUCA1A,GUCY2D,LMX1B,NRTN,PDE6G,PER2,PRPH2,RBP3,RBP4,RDH12,RDH8,RGS11,RHO,RP1L1,RPE65,SAG,STRA6,WT1 | 34 |
| E4 | Development | Cell Morphology, Embryonic Development, Organ Development, Organ Morphology, Organismal Development, Tissue Development, Tissue Morphology, Visual System Development and Function | morphology of retinal cells | 3,2E-12 | AIPL1,CACNA1F,CDKN2A,CNGA3,Cngb1,CNGB3,FAT3,GUCA1A,GUCY2D,PDE6G,PRPH2,RBP3,RDH12,RDH8,RHO,RP1L1,RPE65,SAG,STRA6 | 19 |
| E4 | Development | Cell Morphology, Embryonic Development, Ophthalmic Disease, Organ Development, Organ Morphology, Organismal Development, Organismal Injury and Abnormalities, Tissue Development, Tissue Morphology, Visual System Development and Function | abnormal morphology of photoreceptor outer segments | 1,59E-11 | AIPL1,CNGA3,Cngb1,CNGB3,GUCA1A,GUCY2D,PDE6G,RBP3,RDH12,RHO,RP1L1,RPE65,SAG,STRA6 | 14 |
| E4 | Lipid metabolism | Lipid Metabolism, Small Molecule Biochemistry, Vitamin and Mineral Metabolism | metabolism of terpenoid | 1,22E-19 | AACS,ABCG8,ALDH1A3,APOA4,ATF3,BCO2,CETP,CFTR,CXCL8,CYP11A1,CYP11B1,CYP1A1,CYP1B1,CYP3A7,CYP51A1,DHCR24,DHCR7,EBP,EDN1,FABP6,FDFT1,FDPS,FECH,FGF7,G6PC,HMGCR,HSD17B7,IDI1,INS,INSIG2,LSS,MSMO1,MVK,NR4A1,NSDHL,PLB1,RBP4,RDH12,RDH8,RETSAT,RLBP1,RPE65,SQLE,SREBF1 | 44 |
| E4 | Lipid metabolism | Lipid Metabolism, Molecular Transport, Small Molecule Biochemistry | concentration of lipid | 1,73E-15 | ABCB11,ABCB4,ABCC2,ABCG2,ABCG8,ABHD5,ACSS1,ANGPT2,APOA4,APOD,AQP12A/AQP12B,AQP8,ATF3,CASP1,CD38,CD59,CETP,CFTR,CRY2,CXCL12,CXCL8,CYP11A1,CYP11B1,CYP1A1,CYP1B1,CYP3A7,DBH,DHCR24,DHCR7,EDN1,ESR2,FABP6,FADS2,FDFT1,FGF7,FOS,G6PC,GAL3ST1,GNB3,GPRC6A,HMGCR,HRH1,INS,ITGB3,LIPG,LPL,MFSD2A,MLXIPL,MMP8,MRC1,NME4,NPR1,NPY2R,NR4A1,P2RY2,PLA2G1B,PLVAP,PRF1,PVALB,RAB7A,RBP4,RDH12,RDH8,RPE65,SC5D,SGMS2,SLC34A2,SLC9A3,SREBF1,STRA6,TGM2,TSHR,VCAM1 | 73 |
| E4 | Lipid metabolism | Lipid Metabolism, Small Molecule Biochemistry, Vitamin and Mineral Metabolism | steroid metabolism | 4,4E-15 | AACS,ABCG8,APOA4,ATF3,CETP,CFTR,CXCL8,CYP11A1,CYP11B1,CYP1A1,CYP1B1,CYP3A7,CYP51A1,DHCR24,DHCR7,EBP,EDN1,FABP6,FDFT1,FDPS,FECH,FGF7,G6PC,HMGCR,HSD17B7,IDI1,INS,INSIG2,LSS,MSMO1,MVK,NR4A1,NSDHL,SQLE,SREBF1 | 35 |
| E4 | Lipid metabolism | Lipid Metabolism, Small Molecule Biochemistry, Vitamin and Mineral Metabolism | synthesis of terpenoid | 9,27E-15 | ABCG8,ALDH1A3,ANGPT1,ANGPT2,ATF3,CETP,CFTR,CXCL8,CYP11A1,CYP11B1,CYP1A1,CYP1B1,CYP51A1,DHCR24,DHCR7,EBP,EDN1,ESR2,FDFT1,FDPS,FGF7,FOXL2,G6PC,HMGCR,HRH1,HSD17B7,IDI1,INS,INSIG2,LSS,MALRD1,MVD,MVK,NPR1,NR4A1,RDH8,SCD5,SREBF1 | 38 |
| E4 | Lipid metabolism | Lipid Metabolism, Small Molecule Biochemistry | synthesis of lipid | 1,08E-14 | ABCG8,ABHD5,ACSL5,ACSS1,ACSS2,ALDH1A3,ANGPT1,ANGPT2,APOA4,ATF3,C5AR1,CASP1,CD82,CETP,CFTR,CXCL12,CXCL8,CYP11A1,CYP11B1,CYP1A1,CYP1B1,CYP51A1,DHCR24,DHCR7,EBP,EDN1,ELOVL6,ESR2,FADS2,FDFT1,FDPS,FGF7,FGFR3,FOS,FOSL1,FOXL2,G6PC,GAL3ST1,GBGT1,GGT1,GGT5,GRM5,HMGCR,HRH1,HSD17B7,IAPP,IDI1,INS,INSIG2,LPL,LSS,MALRD1,MLXIPL,MVD,MVK,NPR1,NR4A1,PGF,PLA2G1B,PTX3,PVALB,RDH8,RLBP1,SCD5,SGMS2,SREBF1 | 66 |
| E4 | Lipid metabolism | Lipid Metabolism, Small Molecule Biochemistry, Vitamin and Mineral Metabolism | metabolism of cholesterol | 1,09E-14 | ABCG8,APOA4,CETP,CFTR,CYP11A1,CYP51A1,DHCR24,DHCR7,EBP,FDFT1,FDPS,FECH,HMGCR,HSD17B7,IDI1,INS,INSIG2,LSS,MVK,NSDHL,SQLE,SREBF1 | 22 |
| E4 | Lipid metabolism | Lipid Metabolism, Small Molecule Biochemistry, Vitamin and Mineral Metabolism | synthesis of steroid | 1,24E-14 | ABCG8,ANGPT1,ANGPT2,ATF3,CETP,CFTR,CXCL8,CYP11A1,CYP11B1,CYP1A1,CYP1B1,CYP51A1,DHCR24,DHCR7,EBP,EDN1,ESR2,FDFT1,FDPS,FGF7,FOXL2,G6PC,HMGCR,HRH1,HSD17B7,IDI1,INS,INSIG2,LSS,MALRD1,MVK,NPR1,NR4A1,RDH8,SCD5,SREBF1 | 36 |
| E4 | Lipid metabolism | Lipid Metabolism, Small Molecule Biochemistry, Vitamin and Mineral Metabolism | synthesis of cholesterol | 1,37E-13 | ABCG8,CETP,CFTR,CYP51A1,DHCR24,DHCR7,EBP,FDFT1,FDPS,HMGCR,HSD17B7,IDI1,INS,INSIG2,LSS,MVK,SREBF1 | 17 |
| E4 | Lipid metabolism | Lipid Metabolism, Small Molecule Biochemistry, Vitamin and Mineral Metabolism | synthesis of sterol | 1,09E-12 | ABCG8,CETP,CFTR,CYP51A1,DHCR24,DHCR7,EBP,FDFT1,FDPS,HMGCR,HSD17B7,IDI1,INS,INSIG2,LSS,MVK,SCD5,SREBF1 | 18 |
| E4 | Lipid metabolism | Lipid Metabolism, Small Molecule Biochemistry | metabolism of membrane lipid derivative | 2,89E-12 | ABCG8,ABHD5,ACSL5,APOA4,CD82,CETP,CFTR,CXCL8,CYP11A1,CYP51A1,DHCR24,DHCR7,EBP,EDN1,ENPP7,FDFT1,FDPS,FECH,FGF7,FOS,FOSL1,GAL3ST1,HMGCR,HSD17B7,IAPP,IDI1,INS,INSIG2,LPL,LSS,MVK,NSDHL,PGF,PLA2G1B,PLB1,PTX3,SCD5,SGMS2,SQLE,SREBF1 | 40 |
| E4 | Molecular transport | Lipid Metabolism, Molecular Transport, Small Molecule Biochemistry | concentration of lipid | 1,73E-15 | ABCB11,ABCB4,ABCC2,ABCG2,ABCG8,ABHD5,ACSS1,ANGPT2,APOA4,APOD,AQP12A/AQP12B,AQP8,ATF3,CASP1,CD38,CD59,CETP,CFTR,CRY2,CXCL12,CXCL8,CYP11A1,CYP11B1,CYP1A1,CYP1B1,CYP3A7,DBH,DHCR24,DHCR7,EDN1,ESR2,FABP6,FADS2,FDFT1,FGF7,FOS,G6PC,GAL3ST1,GNB3,GPRC6A,HMGCR,HRH1,INS,ITGB3,LIPG,LPL,MFSD2A,MLXIPL,MMP8,MRC1,NME4,NPR1,NPY2R,NR4A1,P2RY2,PLA2G1B,PLVAP,PRF1,PVALB,RAB7A,RBP4,RDH12,RDH8,RPE65,SC5D,SGMS2,SLC34A2,SLC9A3,SREBF1,STRA6,TGM2,TSHR,VCAM1 | 73 |
| E4 | Molecular transport | Lipid Metabolism, Molecular Transport, Small Molecule Biochemistry | quantity of steroid | 1,46E-11 | ABCB11,ABCB4,ABCC2,ABCG8,ANGPT2,APOA4,AQP12A/AQP12B,AQP8,ATF3,CASP1,CETP,CRY2,CYP11A1,CYP11B1,CYP3A7,DBH,DHCR24,DHCR7,EDN1,ESR2,FABP6,FDFT1,G6PC,GPRC6A,HMGCR,INS,ITGB3,LIPG,LPL,MLXIPL,MRC1,NPR1,NPY2R,P2RY2,PLA2G1B,PLVAP,PVALB,RAB7A,SC5D,SLC34A2,SLC9A3,SREBF1,TGM2,TSHR,VCAM1 | 45 |
| E4 | Molecular transport | Molecular Transport | transport of molecule | 3,33E-10 | ABCA12,ABCB11,ABCB4,ABCC2,ABCG2,ABCG8,ABHD5,ACHE,ACSL5,AHCYL1,ANGPT1,ANGPT2,APOA4,AQP4,AQP8,ARF5,ATF3,B2M,BEST1,BMPER,CA14,CASP1,CCR5,CETP,CFTR,CNGA3,CNGB3,CPEB1,CRAT,CRY2,CXCL12,CXCL8,DBH,DENND3,DRD4,EDN1,ESR2,FABP6,FECH,FGF7,FHL1,FTMT,G6PC,GABRA4,GLUL,HCN4,INS,IRF3,ITGB3,KCNH1,KCNQ5,KCNT1,KCNV1,LIPG,LPL,LSS,MFSD2A,NFATC1,NKX2-5,NKX3-1,NMB,NPPC,NPY2R,NR4A1,NRTN,P2RX3,P2RY2,PER2,PLA2G1B,PLIN3,RAB11B,RAB7A,RBP4,RHAG,RHCG,RICTOR,SGMS2,SLC12A3,SLC14A2,SLC16A3,SLC16A6,SLC22A4,SLC22A7,SLC23A1,SLC25A22,SLC26A6,SLC28A1,SLC34A2,SLC45A4,SLC5A3,SLC6A19,SLC9A3,SREBF1,SSTR3,STRA6,TSHR,UNC93B1 | 97 |
| E4 | Molecular transport | Lipid Metabolism, Molecular Transport, Small Molecule Biochemistry | concentration of cholesterol | 0,000000589 | ABCB11,ABCB4,ABCG8,APOA4,AQP12A/AQP12B,AQP8,CASP1,CETP,DHCR24,DHCR7,ESR2,FABP6,FDFT1,G6PC,HMGCR,ITGB3,LIPG,LPL,MLXIPL,PLA2G1B,PLVAP,RAB7A,SC5D,SREBF1,TGM2,VCAM1 | 26 |
| E4 | Molecular transport | Lipid Metabolism, Molecular Transport, Small Molecule Biochemistry | secretion of lipid | 0,0000015 | ABCA12,ABCB11,ABCB4,ABCC2,ABCG8,ABHD5,ANGPT2,APOA4,ATF3,CXCL8,DRD4,EDN1,ESR2,FGF7,INS,NMB,P2RY2,PLA2G1B,RICTOR,SREBF1 | 20 |
| E4 | Molecular transport | Lipid Metabolism, Molecular Transport, Small Molecule Biochemistry | uptake of lipid | 0,00000218 | ABCB4,ABCG8,ACSL5,APOA4,APOD,CASP1,CETP,CFTR,EDN1,ESR2,FABP6,IAPP,INS,LPL,PLA2G1B,SLC22A7,SREBF1,STRA6 | 18 |
| E4 | Molecular transport | Lipid Metabolism, Molecular Transport, Small Molecule Biochemistry | concentration of acylglycerol | 0,00000434 | ABCG8,ABHD5,APOA4,APOD,AQP8,CASP1,CETP,EDN1,ESR2,FABP6,FADS2,FGF7,G6PC,GPRC6A,HRH1,INS,LIPG,LPL,MFSD2A,MLXIPL,MMP8,NR4A1,PLA2G1B,PLVAP,PRF1,SGMS2,SREBF1 | 27 |
| E4 | Molecular transport | Lipid Metabolism, Molecular Transport, Small Molecule Biochemistry | transport of lipid | 0,00000579 | ABCA12,ABCB11,ABCB4,ABCC2,ABCG2,ABCG8,ACSL5,APOA4,AQP8,CETP,CFTR,CRAT,FABP6,ITGB3,LIPG,LPL,LSS,MFSD2A,RBP4,SREBF1,STRA6 | 21 |
| E4 | Molecular transport | Lipid Metabolism, Molecular Transport, Small Molecule Biochemistry | concentration of triacylglycerol | 0,00000676 | ABCG8,ABHD5,APOA4,APOD,AQP8,CASP1,CETP,ESR2,FABP6,FADS2,FGF7,G6PC,GPRC6A,HRH1,INS,LIPG,LPL,MFSD2A,MLXIPL,MMP8,NR4A1,PLA2G1B,PLVAP,PRF1,SREBF1 | 25 |
| E4 | Molecular transport | Molecular Transport | quantity of metal | 0,0000296 | ADRA1A,ANGPT2,AQP4,B2M,BMPER,C5AR1,CCR5,CD38,CD59,CXCL12,CXCL8,CXCR4,CYP11A1,CYP11B1,EDN1,FGF7,FOS,GPRC6A,GRM5,Gucy2d,IAPP,IGKC,INS,MCOLN3,NMB,NPPC,P2RY2,RHAG,RHCG,SELE,SHC1,SLC12A3,SWAP70,TGM2,VCAM1 | 35 |
| E5 | Development | Organismal Development | morphology of body cavity | 1,39E-11 | ADAMTS1,AQP12A/AQP12B,ARG2,ATF3,B2M,BCL6,CCBE1,CCR7,CD38,CEL,CKMT2,CSF1,CSRP3,CTC1,CTGF,CXCR4,CYBRD1,CYP1A1,CYP24A1,CYP26A1,CYP51A1,DHCR7,DRD1,EIF2AK3,ETS2,F9,FADS2,FDFT1,FGF18,FGL1,FLT1,FMOD,FOS,GADD45G,Gcg,GDNF,GPRC6A,HSD17B7,HSP90B1,IFT122,IGFBP1,INS,IRF1,IRF4,JAG2,JUNB,KCNE2,LAMC2,LIPC,LMO2,LOX,LPL,MOV10L1,MSH5,Nkx6-3,NLRP3,PABPC1L,PDX1,PLA2G1B,PLVAP,PPP1R3C,PRF1,PTGER4,PTGIS,PTGS2,RDH10,RELA,RRAD,S100A1,SERPINE1,SLC22A4,SLC2A9,SLC6A4,SOCS3,SPDEF,SYCP1,TBX3,TGM2,TNFSF12,TRADD,TRIB1,USP21,VIPR1,ZC3H12A | 84 |
| E5 | Development | Cell Morphology, Embryonic Development, Organ Development, Organ Morphology, Organismal Development, Tissue Development, Tissue Morphology, Visual System Development and Function | morphology of photoreceptors | 2,05E-11 | AIPL1,ARL6,CACNA1F,CNGA3,Cngb1,CNGB3,CPLX3,GRK1,MPP4,PDE6G,PRPH2,RDH8,RHO,RP1L1,RPE65,RPGR | 16 |
| E5 | Development | Cell Morphology, Embryonic Development, Ophthalmic Disease, Organ Development, Organ Morphology, Organismal Development, Organismal Injury and Abnormalities, Tissue Development, Tissue Morphology, Visual System Development and Function | abnormal morphology of photoreceptors | 4,2E-11 | AIPL1,ARL6,CACNA1F,CNGA3,Cngb1,CNGB3,CPLX3,GRK1,MPP4,PDE6G,RDH8,RHO,RP1L1,RPE65,RPGR | 15 |
| E5 | Development | Embryonic Development, Organ Development, Organismal Development, Tissue Development, Visual System Development and Function | formation of eye | 6,41E-11 | AIPL1,ALDH1A3,ARL6,CACNA1F,CASP6,CNGA3,Cngb1,CNGB3,CPLX3,CRYAA/LOC102724652,CRYBB2,CRYGA,CXCL8,CYP1B1,FMOD,FOS,GJA3,GJA8,GNAT2,GNGT1,GRK1,IFT122,INHBB,MFN2,MPP4,PDE6C,PDE6G,PER2,PRPH2,RAX,RDH10,RDH8,RHO,RP1L1,RPE65,RPGR,SERPINE1,TRPM1 | 38 |
| E5 | Development | Embryonic Development, Organismal Development | development of head | 5,4E-10 | AIPL1,ALDH1A3,ARL6,ASPN,CACNA1F,CASP6,CLDN5,CNGA3,Cngb1,CNGB3,CPLX3,CRTAC1,CRYAA/LOC102724652,CRYBB2,CRYGA,CSF1,CXCL8,CXCR4,CYP1B1,CYP26A1,DHCR7,DNAH5,DRD1,FMOD,FOS,FOXJ1,FRZB,GDNF,GJA3,GJA8,GNAT2,GNGT1,GRK1,HOXA13,HOXB1,HSD17B7,IFT122,IGFBP1,INHBB,INSIG2,JAG2,MFN2,MPP4,PDE6C,PDE6G,PER2,PRPH2,RAX,RDH10,RDH8,RHO,RP1L1,RPE65,RPGR,SERPINE1,SKOR2,SLC17A7,SLC6A4,SOSTDC1,TBX3,TRPM1,WNT3A | 62 |
| E5 | Development | Embryonic Development, Organ Development, Organismal Development, Tissue Development | development of sensory organ | 7,26E-10 | AIPL1,ALDH1A3,ARL6,CACNA1F,CASP6,CNGA3,Cngb1,CNGB3,CPLX3,CRYAA/LOC102724652,CRYBB2,CRYGA,CXCL8,CYP1B1,FMOD,FOS,FRZB,GJA3,GJA8,GNAT2,GNGT1,GRK1,HOXA13,IFT122,INHBB,INSIG2,MFN2,MPP4,PDE6C,PDE6G,PER2,PRPH2,RAX,RDH10,RDH8,RHO,RP1L1,RPE65,RPGR,SERPINE1,TRPM1,WNT3A | 42 |
| E5 | Development | Cell Morphology, Embryonic Development, Organ Development, Organ Morphology, Organismal Development, Tissue Development, Visual System Development and Function | morphology of eye cells | 8,75E-10 | AIPL1,ARL6,CACNA1F,CNGA3,Cngb1,CNGB3,CPLX3,GJA8,GRK1,MPP4,PDE6G,PRPH2,RDH8,RHO,RP1L1,RPE65,RPGR | 17 |
| E5 | Development | Cellular Development, Cellular Growth and Proliferation, Embryonic Development, Organ Development, Organismal Development, Tissue Development, Visual System Development and Function | formation of eye cells | 1,45E-09 | AIPL1,CNGA3,GJA3,GJA8,GNAT2,GNGT1,GRK1,PDE6C,PRPH2,RHO,RP1L1,RPGR,TRPM1 | 13 |
| E5 | Development | Embryonic Development, Ophthalmic Disease, Organ Development, Organ Morphology, Organismal Development, Organismal Injury and Abnormalities, Tissue Development, Visual System Development and Function | abnormal morphology of eye | 3,37E-09 | AIPL1,ALDH1A3,ARL6,CACNA1F,CNGA3,Cngb1,CNGB3,CPLX3,CRYAA/LOC102724652,CRYBB2,CYP1B1,FMOD,FOS,GJA8,GRK1,INHBB,MPP4,PDE6G,PER2,RDH10,RDH8,RHO,RP1L1,RPE65,RPGR | 25 |
| E5 | Development | Embryonic Development, Organismal Development | development of body axis | 3,41E-09 | AIPL1,ALDH1A3,ARL6,ASPN,CACNA1F,CASP6,CLDN5,CNGA3,Cngb1,CNGB3,CPLX3,CRTAC1,CRYAA/LOC102724652,CRYBB2,CRYGA,CSF1,CXCL8,CXCR4,CYP1B1,CYP26A1,DHCR7,DNAH5,DRD1,FMOD,FOS,FOXJ1,FRZB,GDNF,GJA3,GJA8,GNAT2,GNGT1,GRK1,HOXA13,HOXB1,HSD17B7,HSP90B1,IFT122,IGFBP1,INHBB,INSIG2,JAG2,MFN2,MPP4,PDE6C,PDE6G,PER2,PRPH2,RAX,RDH10,RDH8,RHO,RP1L1,RPE65,RPGR,SERPINE1,SKOR2,SLC17A7,SLC6A4,SOSTDC1,TBX3,TRPM1,WNT3A | 63 |
| E5 | Lipid metabolism | Lipid Metabolism, Small Molecule Biochemistry, Vitamin and Mineral Metabolism | metabolism of terpenoid | 2,83E-16 | AACS,ALDH1A3,APOA4,ATF3,CEL,CXCL8,CYP1A1,CYP1B1,CYP24A1,CYP26A1,CYP3A7,CYP51A1,DHCR24,DHCR7,EBP,FDFT1,FDPS,FGL1,HMGCR,HSD17B7,IDI1,INS,INSIG2,LIPC,LSS,MSMO1,MVK,NSDHL,PMVK,PTGIS,RDH10,RDH8,RPE65,SLC27A5,SQLE,SREBF1,TNFSF12,TTR | 38 |
| E5 | Lipid metabolism | Lipid Metabolism, Small Molecule Biochemistry, Vitamin and Mineral Metabolism | metabolism of sterol | 4,81E-15 | APOA4,CEL,CYP24A1,CYP51A1,DHCR24,DHCR7,EBP,FDFT1,FDPS,FGL1,HMGCR,HSD17B7,IDI1,INS,INSIG2,LIPC,LSS,MVK,NSDHL,PMVK,SQLE,SREBF1 | 22 |
| E5 | Lipid metabolism | Lipid Metabolism, Small Molecule Biochemistry, Vitamin and Mineral Metabolism | metabolism of cholesterol | 2E-14 | APOA4,CEL,CYP51A1,DHCR24,DHCR7,EBP,FDFT1,FDPS,FGL1,HMGCR,HSD17B7,IDI1,INS,INSIG2,LIPC,LSS,MVK,NSDHL,PMVK,SQLE,SREBF1 | 21 |
| E5 | Lipid metabolism | Lipid Metabolism, Small Molecule Biochemistry, Vitamin and Mineral Metabolism | steroid metabolism | 6,94E-14 | AACS,APOA4,ATF3,CEL,CXCL8,CYP1A1,CYP1B1,CYP24A1,CYP3A7,CYP51A1,DHCR24,DHCR7,EBP,FDFT1,FDPS,FGL1,HMGCR,HSD17B7,IDI1,INS,INSIG2,LIPC,LSS,MSMO1,MVK,NSDHL,PMVK,PTGIS,SLC27A5,SQLE,SREBF1,TNFSF12 | 32 |
| E5 | Lipid metabolism | Lipid Metabolism, Small Molecule Biochemistry, Vitamin and Mineral Metabolism | synthesis of cholesterol | 5,03E-13 | CEL,CYP51A1,DHCR24,DHCR7,EBP,FDFT1,FDPS,HMGCR,HSD17B7,IDI1,INS,INSIG2,LSS,MVK,PMVK,SREBF1 | 16 |
| E5 | Lipid metabolism | Lipid Metabolism, Molecular Transport, Small Molecule Biochemistry | concentration of lipid | 2,1E-12 | ABHD6,APOA4,APOD,AQP12A/AQP12B,AQP9,ARF1,ATF3,CD38,CEL,CHRNA7,CRY2,CSF1,CTSS,CXCL8,CYP1A1,CYP1B1,CYP24A1,CYP26A1,CYP3A7,DHCR24,DHCR7,EEF1A2,FADS2,FASN,FDFT1,FGL1,FOS,GAL,GNB3,GPRC6A,HMGCR,HNF4A,INPP5K,INS,KCNA6,LIPC,LIPG,LPL,MAG,MMP8,MRC1,NPY,PLA2G1B,PLVAP,PPP1R3C,PRF1,PTGDR2,PTGER4,PTGIS,PTGS2,PVALB,RDH8,RPE65,RRAD,SERPINE1,SLC34A2,SLC6A4,SREBF1,TACR1,TGM2,TNFSF12,TRIB1,TTR | 63 |
| E5 | Lipid metabolism | Lipid Metabolism, Small Molecule Biochemistry, Vitamin and Mineral Metabolism | synthesis of sterol | 2,95E-12 | CEL,CYP24A1,CYP51A1,DHCR24,DHCR7,EBP,FDFT1,FDPS,HMGCR,HSD17B7,IDI1,INS,INSIG2,LSS,MVK,PMVK,SREBF1 | 17 |
| E5 | Lipid metabolism | Lipid Metabolism, Small Molecule Biochemistry | metabolism of membrane lipid derivative | 3,22E-12 | ABHD6,APOA4,ARF1,CD82,CEL,CXCL8,CYP24A1,CYP51A1,DHCR24,DHCR7,DRD1,EBP,EIF2AK3,FASN,FDFT1,FDPS,FGL1,FLT1,FOS,FOSL1,HMGCR,HSD17B7,IDI1,INS,INSIG2,LIPC,LPL,LSS,MVK,NSDHL,PLA2G1B,PLA2G4C,PLCH2,PMVK,PTGS2,SOCS3,SQLE,SREBF1 | 38 |
| E5 | Lipid metabolism | Lipid Metabolism, Small Molecule Biochemistry, Vitamin and Mineral Metabolism | synthesis of terpenoid | 5,48E-11 | ALDH1A3,ATF3,CEL,CSF1,CXCL8,CYP1A1,CYP1B1,CYP24A1,CYP26A1,CYP51A1,DHCR24,DHCR7,EBP,FDFT1,FDPS,HMGCR,HNF4A,HSD17B7,IDI1,INS,INSIG2,LSS,MALRD1,MVD,MVK,PMVK,RDH10,RDH8,SLC27A5,SREBF1,TNFSF12 | 31 |
| E5 | Lipid metabolism | Lipid Metabolism, Small Molecule Biochemistry | synthesis of lipid | 1,78E-09 | ABHD6,ACSS2,ALDH1A3,APOA4,ARF1,ATF3,C5AR1,CD82,CEL,CSF1,CXCL8,CYP1A1,CYP1B1,CYP24A1,CYP26A1,CYP51A1,DHCR24,DHCR7,EBP,EIF2AK3,FADS2,FASN,FDFT1,FDPS,FLT1,FOS,FOSL1,HMGCR,HNF4A,HSD17B7,IDI1,INS,INSIG2,LIPC,LPL,LSS,MALRD1,MVD,MVK,PLA2G1B,PMVK,PTGIS,PTGS2,PVALB,RDH10,RDH8,RELA,SLC27A5,SOCS3,SREBF1,TACR1,TNFSF12 | 52 |
| E5 | Molecular transport | Lipid Metabolism, Molecular Transport, Small Molecule Biochemistry | concentration of lipid | 2,1E-12 | ABHD6,APOA4,APOD,AQP12A/AQP12B,AQP9,ARF1,ATF3,CD38,CEL,CHRNA7,CRY2,CSF1,CTSS,CXCL8,CYP1A1,CYP1B1,CYP24A1,CYP26A1,CYP3A7,DHCR24,DHCR7,EEF1A2,FADS2,FASN,FDFT1,FGL1,FOS,GAL,GNB3,GPRC6A,HMGCR,HNF4A,INPP5K,INS,KCNA6,LIPC,LIPG,LPL,MAG,MMP8,MRC1,NPY,PLA2G1B,PLVAP,PPP1R3C,PRF1,PTGDR2,PTGER4,PTGIS,PTGS2,PVALB,RDH8,RPE65,RRAD,SERPINE1,SLC34A2,SLC6A4,SREBF1,TACR1,TGM2,TNFSF12,TRIB1,TTR | 63 |
| E5 | Molecular transport | Lipid Metabolism, Molecular Transport, Small Molecule Biochemistry | quantity of steroid | 1,71E-08 | APOA4,AQP12A/AQP12B,ATF3,CEL,CRY2,CSF1,CTSS,CYP24A1,CYP3A7,DHCR24,DHCR7,EEF1A2,FDFT1,FGL1,GAL,GPRC6A,HMGCR,HNF4A,INPP5K,INS,LIPC,LIPG,LPL,MRC1,NPY,PLA2G1B,PLVAP,PTGS2,PVALB,SERPINE1,SLC34A2,SLC6A4,SREBF1,TACR1,TGM2,TNFSF12,TRIB1 | 37 |
| E5 | Molecular transport | Molecular Transport | transport of molecule | 0,000000134 | ANO9,APOA4,AQP4,AQP9,ARF1,ARF5,ATF3,B2M,BCL6,CACNB1,CAMK2A,CCR7,CHRM4,CHRNA7,CNGA3,CNGB3,CPLX3,CRY2,CTSS,CXCL8,CYBRD1,DPP6,DRD1,FOSB,GABRA2,GABRB2,GAL,GALR1,GDNF,GJA8,HCN4,INPP5K,INS,KCNA6,KCNB1,KCNE2,KCNH1,KCNH4,KCNH6,KCNIP3,KCNJ1,KCNV1,LIPC,LIPG,LPL,LSS,MIP,NFKBIE,NMB,NPY,PEA15,PER2,PLA2G1B,PLIN3,PML,PPP1R3B,PPP1R3C,PTGER4,PTGS2,RHBG,RPGR,SLC16A3,SLC17A7,SLC1A7,SLC22A4,SLC27A5,SLC2A5,SLC2A9,SLC32A1,SLC34A2,SLC6A1,SLC6A18,SLC6A4,SOCS3,SQSTM1,SREBF1,TACR1,TNFAIP8L3,TRIB1,TRPM1,TTR,UNC13D,ZC3H12A | 83 |
| E5 | Molecular transport | Lipid Metabolism, Molecular Transport, Small Molecule Biochemistry | concentration of triacylglycerol | 0,00000138 | APOA4,APOD,AQP9,FADS2,FASN,FGL1,GPRC6A,HNF4A,INPP5K,INS,KCNA6,LIPC,LIPG,LPL,MMP8,NPY,PLA2G1B,PLVAP,PPP1R3C,PRF1,PTGS2,RRAD,SERPINE1,SREBF1,TRIB1 | 25 |
| E5 | Molecular transport | Lipid Metabolism, Molecular Transport, Small Molecule Biochemistry | concentration of acylglycerol | 0,00000255 | ABHD6,APOA4,APOD,AQP9,FADS2,FASN,FGL1,GPRC6A,HNF4A,INPP5K,INS,KCNA6,LIPC,LIPG,LPL,MMP8,NPY,PLA2G1B,PLVAP,PPP1R3C,PRF1,PTGS2,RRAD,SERPINE1,SREBF1,TRIB1 | 26 |
| E5 | Molecular transport | Lipid Metabolism, Molecular Transport, Small Molecule Biochemistry | concentration of sterol | 0,00000419 | APOA4,AQP12A/AQP12B,CEL,CSF1,CTSS,CYP24A1,DHCR24,DHCR7,EEF1A2,FDFT1,FGL1,HMGCR,HNF4A,INPP5K,LIPC,LIPG,LPL,PLA2G1B,PLVAP,PTGS2,SERPINE1,SREBF1,TGM2,TRIB1 | 24 |
| E5 | Molecular transport | Lipid Metabolism, Molecular Transport, Small Molecule Biochemistry | concentration of cholesterol | 0,00000456 | APOA4,AQP12A/AQP12B,CEL,CSF1,CTSS,DHCR24,DHCR7,EEF1A2,FDFT1,FGL1,HMGCR,HNF4A,INPP5K,LIPC,LIPG,LPL,PLA2G1B,PLVAP,PTGS2,SERPINE1,SREBF1,TGM2,TRIB1 | 23 |
| E5 | Molecular transport | Molecular Transport | quantity of metal | 0,000011 | ADAMTS1,AQP4,ARF1,B2M,C5AR1,CAMK2A,CD38,CHRNA7,CSF1,CXCL8,CXCR4,CYBRD1,DRD1,EIF2AK3,FOS,GAL,GPRC6A,Gucy2d,HNF4A,INS,KCNJ1,MAG,NMB,NPY,PDX1,PTGDR2,PTGS2,PYY,S100A1,SLC2A9,TACR1,TGM2,TRIB1,TTR | 34 |
| E5 | Molecular transport | Molecular Transport | quantity of metal ion | 0,0000417 | ADAMTS1,AQP4,C5AR1,CAMK2A,CD38,CHRNA7,CSF1,CXCL8,CXCR4,DRD1,EIF2AK3,FOS,GAL,GPRC6A,Gucy2d,HNF4A,INS,KCNJ1,MAG,NMB,NPY,PDX1,PTGDR2,PTGS2,PYY,S100A1,SLC2A9,TACR1,TGM2,TTR | 30 |
| E5 | Molecular transport | Molecular Transport | transport of K+ | 0,000078 | DPP6,KCNA6,KCNB1,KCNE2,KCNH1,KCNH4,KCNH6,KCNIP3,KCNJ1,KCNV1 | 10 |
| E6 | Development | Embryonic Development, Organ Development, Organ Morphology, Organismal Development, Tissue Development, Tissue Morphology, Visual System Development and Function | morphology of retinal tissue | 1,43E-09 | ARL6,CNGA3,GRK1,GUCA1A,HES5,MPP4,PAX6,PDE6G,PRPH2,RDH8,RHO,RP1L1,RS1,SIX3 | 14 |
| E6 | Development | Cell Morphology, Embryonic Development, Organ Development, Organ Morphology, Organismal Development, Tissue Development, Tissue Morphology, Visual System Development and Function | morphology of photoreceptors | 9,07E-09 | ARL6,CNGA3,GRK1,GUCA1A,MPP4,PDE6G,PRPH2,RDH8,RHO,RP1L1,RS1,SIX3 | 12 |
| E6 | Development | Cell Morphology, Embryonic Development, Organ Development, Organ Morphology, Organismal Development, Tissue Development, Visual System Development and Function | morphology of eye cells | 1,03E-08 | ARL6,CNGA3,GRK1,GUCA1A,HES5,MPP4,PAX6,PDE6G,PRPH2,RDH8,RHO,RP1L1,RS1,SIX3 | 14 |
| E6 | Development | Cell Morphology, Embryonic Development, Organ Development, Organ Morphology, Organismal Development, Tissue Development, Tissue Morphology, Visual System Development and Function | morphology of retinal cells | 1,24E-08 | ARL6,CNGA3,GRK1,GUCA1A,HES5,MPP4,PDE6G,PRPH2,RDH8,RHO,RP1L1,RS1,SIX3 | 13 |
| E6 | Development | Cellular Development, Cellular Growth and Proliferation, Embryonic Development, Organ Development, Organismal Development, Tissue Development, Visual System Development and Function | formation of retinal cells | 5,13E-08 | CNGA3,GNAT2,GRK1,PAX6,PDE6C,PRPH2,RHO,RP1L1,RS1,SIX3 | 10 |
| E6 | Development | Embryonic Development, Organ Development, Organismal Development, Tissue Development | development of sensory organ | 6,84E-08 | ALDH1A3,ARL6,ATOH7,CNGA3,CRYAA/LOC102724652,CXCL8,DLL1,FOS,FOSL2,FRZB,GNAT2,GRK1,GRXCR1,GUCA1A,HES5,INHBB,KLF2,MFN2,MPP4,PAX6,PDE6C,PDE6G,PLPPR4,PRPH2,RDH8,RHO,RP1L1,RS1,SERPINE1,SIX3,SIX6,TIMP2 | 32 |
| E6 | Development | Embryonic Development, Organ Development, Organismal Development, Tissue Development, Visual System Development and Function | formation of eye | 0,000000145 | ALDH1A3,ARL6,ATOH7,CNGA3,CRYAA/LOC102724652,CXCL8,FOS,FOSL2,GNAT2,GRK1,GUCA1A,HES5,INHBB,MFN2,MPP4,PAX6,PDE6C,PDE6G,PRPH2,RDH8,RHO,RP1L1,RS1,SERPINE1,SIX3,SIX6,TIMP2 | 27 |
| E6 | Development | Cellular Development, Cellular Growth and Proliferation, Embryonic Development, Organ Development, Organismal Development, Tissue Development, Visual System Development and Function | development of photoreceptors | 0,000000153 | CNGA3,GNAT2,GRK1,PAX6,PDE6C,PRPH2,RHO,RP1L1,RS1 | 9 |
| E6 | Development | Embryonic Development, Organismal Development | development of head | 0,000000169 | ACVR2B,ALDH1A3,ARL6,ATOH7,CASR,CCND2,CLDN5,CNGA3,CRTAC1,CRYAA/LOC102724652,CXCL8,CXCR4,DIO3,DKK1,DLL1,EFNA3,FOS,FOSL2,FRZB,GNAT2,GRK1,GRXCR1,GUCA1A,HES5,INHBB,KLF2,LOXL3,MFN2,MPP4,NODAL,PAX6,PDE6C,PDE6G,PLPPR4,PRPH2,RDH8,RHO,RP1L1,RS1,SERPINE1,SIX3,SIX6,SLC1A2,TIMP2,TLR2,TRPM6 | 46 |
| E6 | Development | Embryonic Development, Organismal Development | development of body axis | 0,000000518 | ACVR2B,ALDH1A3,ARL6,ATOH7,CASR,CCND2,CLDN5,CNGA3,CRTAC1,CRYAA/LOC102724652,CXCL8,CXCR4,DIO3,DKK1,DLL1,EFNA3,FOS,FOSL2,FRZB,GNAT2,GRK1,GRXCR1,GUCA1A,HES5,HSP90B1,INHBB,KLF2,LOXL3,MFN2,MPP4,NODAL,PAX6,PDE6C,PDE6G,PLPPR4,PRPH2,RDH8,RHO,RP1L1,RS1,SERPINE1,SIX3,SIX6,SLC1A2,TIMP2,TLR2,TRPM6 | 47 |

Supplementary file 1C

|  |  | **Low dose** |  | **Pulse dose** |  | **High dose** |  |
| --- | --- | --- | --- | --- | --- | --- | --- |
| **Stage** | **Category** | **Pathway** | **#** | **Pathway** | **#** | **Pathway** | **#** |
| E1 | Canonical pathways | Bupropion Degradation |  | Phospholipases |  | Glucocorticoid Biosynthesis |  |
|  |  | Acetone Degradation I (to Methylglyoxal) |  | Sperm Motility |  | Mineralocorticoid Biosynthesis |  |
|  |  | Glycolysis I |  | Cellular Effects of Sildenafil (Viagra) |  | Androgen Biosynthesis |  |
|  |  | Gluconeogenesis I |  | Aryl Hydrocarbon Receptor Signaling |  | Bupropion Degradation |  |
|  |  | Estrogen Biosynthesis |  | Dopamine-DARPP32 Feedback in cAMP Signaling |  | Acetone Degradation I (to Methylglyoxal) |  |
| E1 | Molecular and cellular function | Lipid Metabolism | 2 | Lipid Metabolism | 4 | Cell Cycle | 1 |
|  |  | Small Molecule Biochemistry | 3 | Small Molecule Biochemistry | 5 | Lipid Metabolism | 3 |
|  |  | Vitamin and Mineral Metabolism | 2 | Vitamin and Mineral Metabolism | 4 | Small Molecule Biochemistry | 3 |
|  |  | Drug Metabolism | 2 | Drug Metabolism | 3 | Vitamin and Mineral Metabolism | 2 |
|  |  | Cell-To-Cell Signaling and Interactio | 2 | Cell Death and Surviva | 2 | Drug Metabolism | 2 |
| E1 | Physiological system development and function | Connective Tissue Development and Function | 3 | Embryonic Development | 5 | Organ Morphology | 2 |
|  |  | Reproductive System Development and Function | 1 | Nervous System Development and Function | 5 | Organismal Development | 2 |
|  |  | Endocrine System Development and Function | 2 | Organ Development | 5 | Skeletal and Muscular System Development and Function | 2 |
|  |  | Cardiovascular System Development and Function | 1 | Organismal Developmen | 7 | Connective Tissue Development and Function | 3 |
|  |  | Tissue Morphology | 2 | Reproductive System Development and Function | 3 | Reproductive System Development and Function | 1 |
| E1 | Cardiotoxicity | Cardiac Hypertrophy | 1 | Cardiac Arrythmia | 1 |  |  |
|  |  | Cardiac Fibrosis | 1 | Cardiac Dysfunction | 1 |  |  |
|  |  |  |  | Heart Failure | 1 |  |  |
|  |  |  |  | Cardiac Arteriopathy | 1 |  |  |
|  |  |  |  | Tachycardia | 1 |  |  |
|  | Sum |  | 2 |  | 5 |  | 0 |
| E2 | Canonical pathways | Bupropion Degradation |  | Gs Signaling |  | Dopamine-DARPP32 Feedback in cAMP Signaling |  |
|  |  | Acetone Degradation I (to Methylglyoxal) |  | TREM1 Signaling |  | Sperm Motility |  |
|  |  | Estrogen Biosynthesis |  | Nitric Oxide Signaling in the Cardiovascular System |  | Neuropathic Pain Signaling In Dorsal Horn Neurons |  |
|  |  | Retinol Biosynthesis |  | cAMP-mediated signaling |  | Sertoli Cell-Sertoli Cell Junction Signaling |  |
|  |  | Nicotine Degradation III |  | Amyotrophic Lateral Sclerosis Signaling |  | Axonal Guidance Signaling |  |
| E2 | Molecular and cellular function | Cell-To-Cell Signaling and Interaction | 25 | Molecular Transport | 80 | Molecular Transport | 117 |
|  |  | Lipid Metabolism | 22 | Cell Morphology | 81 | Cellular Movement | 84 |
|  |  | Small Molecule Biochemistry | 26 | Cell-To-Cell Signaling and Interaction | 73 | Lipid Metabolism | 73 |
|  |  | Vitamin and Mineral Metabolism | 10 | Cellular Movement | 62 | Small Molecule Biochemistry | 86 |
|  |  | Drug Metabolis | 8 | Cellular Function and Maintenance | 83 | Cell-To-Cell Signaling and Interaction | 60 |
| E2 | Physiological system development and function | Nervous System Development and Function | 44 | Behavior |  | Organismal Development | 123 |
|  |  | Behavior | 23 | Nervous System Development and Function | 74 | Organismal Survival | 102 |
|  |  | Endocrine System Development and Function | 8 | Organismal Development | 80 | Behavior | 59 |
|  |  | Skeletal and Muscular System Development and Function | 15 | Tissue Morphology | 74 | Cardiovascular System Development and Function | 81 |
|  |  | Connective Tissue Development and Function | 12 | Embryonic Development | 41 | Embryonic Development | 80 |
| E2 | Cardiotoxicity | Congenital Heart Anomaly | 4 | Cardiac Arrythmia | 10 | Cardiac Fibrosis | 12 |
|  |  | Cardiac Arrythmia | 4 | Tachycardia | 7 | Cardiac Arrythmia | 13 |
|  |  | Tachycardia | 3 | Cardiac Arteriopathy | 11 | Heart Failure | 14 |
|  |  | Heart Failure | 5 | Bradycardia | 3 | Cardiac Congestive Cardiac Failure | 8 |
|  |  | Cardiac Enlargement | 1 | Cardiac Damage | 2 | Cardiac Hypertrophy | 16 |
|  | Sum |  | 17 |  | 33 |  | 63 |
| E3 | Canonical pathways | Aryl Hydrocarbon Receptor Signaling |  | Amyotrophic Lateral Sclerosis Signaling |  | Superpathway of Cholesterol Biosynthesis |  |
|  |  | Bupropion Degradation |  | eNOS Signaling |  | Cholesterol Biosynthesis I |  |
|  |  | Acetone Degradation I (to Methylglyoxal) |  | GPCR-Mediated Integration of Enteroendocrine Signaling Exemplified by an L Cell |  | Cholesterol Biosynthesis II (via 24,25-dihydrolanosterol) |  |
|  |  | Estrogen Biosynthesis |  | Xenobiotic Metabolism Signaling |  | Cholesterol Biosynthesis III (via Desmosterol) |  |
|  |  | Xenobiotic Metabolism Signaling |  | Estrogen Biosynthesis |  | Hepatic Fibrosis / Hepatic Stellate Cell Activation |  |
| E3 | Molecular and cellular function | Lipid Metabolism | 5 | Cell Morphology | 54 | Lipid Metabolism | 115 |
|  |  | Small Molecule Biochemistry | 6 | Lipid Metabolism | 28 | Small Molecule Biochemistry | 123 |
|  |  | Cell-To-Cell Signaling and Interaction | 4 | Small Molecule Biochemistry | 38 | Vitamin and Mineral Metabolism | 80 |
|  |  | Drug Metabolism | 4 | Vitamin and Mineral Metabolism | 24 | Molecular Transport | 139 |
|  |  | Vitamin and Mineral Metabolism | 5 | Drug Metabolism | 16 | Cell Morphology | 116 |
| E3 | Physiological system development and function | Endocrine System Development and Function | 3 | Endocrine System Development and Function | 15 | Organismal Development | 203 |
|  |  | Connective Tissue Development and Function | 3 | Tissue Development | 66 | Digestive System Development and Function | 74 |
|  |  | Skeletal and Muscular System Development and Function | 3 | Humoral Immune Response | 5 | Embryonic Development | 146 |
|  |  | Nervous System Development and Function | 6 | Lymphoid Tissue Structure and Development | 13 | Connective Tissue Development and Function | 98 |
|  |  | Organ Morphology | 5 | Digestive System Development and Function | 23 | Skeletal and Muscular System Development and Function | 101 |
| E3 | Cardiotoxicity | Cardiac Enlargement | 1 | Cardiac Arrythmia | 1 | Cardiac Congestive Cardiac Failure | 11 |
|  |  | Congenital Heart Anomaly | 1 | Congenital Heart Anomaly | 4 | Heart Failure | 18 |
|  |  | Cardiac Proliferation | 1 | Cardiac Enlargement | 1 | Cardiac Infarction | 18 |
|  |  | Pulmonary Hypertension | 1 | Heart Failure | 3 | Cardiac Dysfunction | 14 |
|  |  | Cardiac Hypertrophy | 1 | Cardiac Proliferation | 1 | Cardiac Arteriopathy | 18 |
|  | Sum |  | 5 |  | 10 |  | 79 |
| E4 | Canonical pathways | Aryl Hydrocarbon Receptor Signaling |  | G-Protein Coupled Receptor Signaling |  | Superpathway of Cholesterol Biosynthesis |  |
|  |  | Bupropion Degradation |  | cAMP-mediated signaling |  | Cholesterol Biosynthesis I |  |
|  |  | Acetone Degradation I (to Methylglyoxal) |  | Estrogen Biosynthesis |  | Cholesterol Biosynthesis II (via 24,25-dihydrolanosterol) |  |
|  |  | Estrogen Biosynthesis |  | Serotonin Receptor Signaling |  | Cholesterol Biosynthesis III (via Desmosterol) |  |
|  |  | Nicotine Degradation III |  | Bupropion Degradation |  | Phototransduction Pathway |  |
| E4 | Molecular and cellular function | Lipid Metabolism | 12 | Drug Metabolism | 16 | Lipid Metabolism | 124 |
|  |  | Small Molecule Biochemistry | 17 | Lipid Metabolism | 29 | Small Molecule Biochemistry | 157 |
|  |  | Molecular Transport | 19 | Small Molecule Biochemistry | 49 | Vitamin and Mineral Metabolism | 84 |
|  |  | Drug Metabolism | 6 | Vitamin and Mineral Metabolism | 13 | Molecular Transport | 154 |
|  |  | Vitamin and Mineral Metabolism | 6 | Cell-To-Cell Signaling and Interaction | 32 | Cell Morphology | 128 |
| E4 | Physiological system development and function | Organismal Development | 26 | Cardiovascular System Development and Function | 31 | Nervous System Development and Function | 68 |
|  |  | Connective Tissue Development and Function | 15 | Hematological System Development and Function | 40 | Embryonic Development | 145 |
|  |  | Skeletal and Muscular System Development and Function | 12 | Behavior | 24 | Organ Development | 120 |
|  |  | Tissue Development | 20 | Endocrine System Development and Function | 11 | Organismal Development | 197 |
|  |  | Tissue Morphology | 14 | Immune Cell Trafficking | 23 | Tissue Development | 177 |
| E4 | Cardiotoxicity | Cardiac Output | 1 | Heart Failure | 13 | Cardiac Infarction | 20 |
|  |  | Cardiac Necrosis/Cell Death | 3 | Cardiac Congestive Cardiac Failure | 8 | Cardiac Fibrosis | 11 |
|  |  | Cardiac Dysfunction | 1 | Cardiac Arrythmia | 4 | Cardiac Arteriopathy | 21 |
|  |  | Cardiac Hypertrophy | 3 | Cardiac Arteriopathy | 8 | Cardiac Hypertrophy | 22 |
|  |  | Cardiac Hypoplasia | 1 | Cardiac Infarction | 7 | Heart Failure | 18 |
|  | Sum |  | 9 |  | 40 |  | 92 |
| E5 | Canonical pathways | Airway Pathology in Chronic Obstructive Pulmonary Disease |  | Superpathway of Cholesterol Biosynthesis |  | Superpathway of Cholesterol Biosynthesis |  |
|  |  | Agranulocyte Adhesion and Diapedesis |  | Cholesterol Biosynthesis I |  | Phototransduction Pathway |  |
|  |  | Tight Junction Signaling |  | Cholesterol Biosynthesis II (via 24,25-dihydrolanosterol) |  | Cholesterol Biosynthesis I |  |
|  |  | Granulocyte Adhesion and Diapedesis |  | Cholesterol Biosynthesis III (via Desmosterol) |  | Cholesterol Biosynthesis II (via 24,25-dihydrolanosterol) |  |
|  |  | Bladder Cancer Signaling |  | Superpathway of Geranylgeranyldiphosphate Biosynthesis I (via Mevalonate) |  | Cholesterol Biosynthesis III (via Desmosterol) |  |
| E5 | Molecular and cellular function | Cell Death and Survival | 66 | Lipid Metabolism | 70 | Lipid Metabolism | 103 |
|  |  | Lipid Metabolism | 37 | Small Molecule Biochemistry | 81 | Small Molecule Biochemistry | 133 |
|  |  | Molecular Transport | 40 | Vitamin and Mineral Metabolism | 28 | Vitamin and Mineral Metabolism | 67 |
|  |  | Small Molecule Biochemistry | 52 | Molecular Transport | 73 | Molecular Transport | 129 |
|  |  | Cellular Movement | 45 | Drug Metabolism | 18 | Cell Morphology | 113 |
| E5 | Physiological system development and function | Nervous System Development and Function | 34 | Cardiovascular System Development and Function | 53 | Nervous System Development and Function | 95 |
|  |  | Tissue Morphology | 67 | Reproductive System Development and Function | 37 | Visual System Development and Function | 45 |
|  |  | Organ Morphology | 43 | Organismal Development | 80 | Tissue Morphology | 149 |
|  |  | Visual System Development and Function | 6 | Organismal Survival | 68 | Organismal Development | 183 |
|  |  | Connective Tissue Development and Function | 39 | Tissue Morphology | 72 | Embryonic Development | 117 |
| E5 | Cardiotoxicity | Cardiac Necrosis/Cell Death | 8 | Cardiac Arteriopathy | 13 | Cardiac Infarction | 17 |
|  |  | Cardiac Hypertrophy | 8 | Cardiac Arrythmia | 8 | Cardiac Arteriopathy | 18 |
|  |  | Cardiac Enlargement | 1 | Heart Failure | 11 | Cardiac Arrythmia | 14 |
|  |  | Cardiac Infarction | 5 | Cardiac Infarction | 10 | Heart Failure | 15 |
|  |  | Cardiac Fibrosis | 4 | Cardiac Stenosis | 4 | Cardiac Inflammation | 5 |
|  | Sum |  | 26 |  | 46 |  | 69 |
| E6 | Canonical pathways | CD40 Signaling |  | Phototransduction Pathway |  | Phototransduction Pathway |  |
|  |  | Role of Osteoblasts, Osteoclasts and Chondrocytes in Rheumatoid Arthritis |  | Tumoricidal Function of Hepatic Natural Killer Cells |  | Airway Pathology in Chronic Obstructive Pulmonary Disease8 |  |
|  |  | p53 Signaling |  | Hereditary Breast Cancer Signaling |  | Agranulocyte Adhesion and Diapedesis |  |
|  |  | Role of NANOG in Mammalian Embryonic Stem Cell Pluripotency |  | Cytotoxic T Lymphocyte-mediated Apoptosis of Target Cells |  | Atherosclerosis Signaling |  |
|  |  | UVB-Induced MAPK Signaling |  | Role of Macrophages, Fibroblasts and Endothelial Cells in Rheumatoid Arthritis 1.99E-02 1.9 % 6/315 |  | IL-8 Signaling |  |
| E6 | Molecular and cellular function | Cell Morphology | 32 | Protein Synthesis | 20 | Cell Morphology | 76 |
|  |  | Protein Synthesis | 11 | Cellular Development | 35 | Cellular Growth and Proliferation | 145 |
|  |  | Cellular Function and Maintenance | 25 | Amino Acid Metabolism | 5 | Cellular Development | 95 |
|  |  | Cellular Movement | 29 | Drug Metabolism | 6 | Protein Synthesis | 53 |
|  |  | Gene Expression | 26 | Molecular Transport | 23 | Cellular Movement | 74 |
| E6 | Physiological system development and function | Connective Tissue Development and Function | 22 | Embryonic Development | 44 | Nervous System Development and Function | 73 |
|  |  | Skeletal and Muscular System Development and Function | 15 | Hematological System Development and Function | 29 | Tissue Morphology | 103 |
|  |  | Tissue Development | 31 | Hematopoiesis | 20 | Visual System Development and Function | 41 |
|  |  | Tissue Morphology | 38 | Organismal Development | 54 | Embryonic Development | 78 |
|  |  | Digestive System Development and Function | 18 | Tissue Morphology | 44 | Organ Development | 72 |
| E6 | Cardiotoxicity | Cardiac Arrythmia | 3 | Cardiac Arrythmia | 5 | Cardiac Inflammation | 6 |
|  |  | Cardiac Dysfunction | 3 | Congenital Heart Anomaly | 5 | Cardiac Infarction | 11 |
|  |  | Cardiac Hypertrophy | 3 | Cardiac Arteriopathy | 4 | Cardiac Stenosis | 4 |
|  |  | Cardiac Enlargement | 1 | Heart Failure | 1 | Cardiac Damage | 2 |
|  |  | Congenital Heart Anomaly | 2 | Cardiac Fibrosis | 4 | Heart Failure | 11 |
|  | Sum |  | 12 |  | 19 |  | 34 |

Supplementary file 1D

| **10 most regulated** | **Cod ID** | **Swissprot annotation** | **Sampling point** | **SP ID** | **Reference** |
| --- | --- | --- | --- | --- | --- |
| Embrynoic exposure down | ENSGMOG00000000263 | sp|Q12951|FOXI1_HUMAN Forkhead box protein I1 OS=Homo sapiens GN=FOXI1 PE=2 SV=3" | E2 | *foxi1* | PMID: 24650709, PMID: 25564621 |
| Embrynoic exposure down | ENSGMOG00000005691 | sp|Q68DU8|KCD16_HUMAN BTB/POZ domain-containing protein KCTD16 OS=Homo sapiens GN=KCTD16 PE=2 SV=1" | E2 | *kcd16* | PMID: 21452234 |
| Embrynoic exposure down | ENSGMOG00000001774 | sp|O70528|5HT4R_CAVPO 5-hydroxytryptamine receptor 4 OS=Cavia porcellus GN=HTR4 PE=2 SV=1" | E2 | *5ht4r* | PMID: 23307014 |
| Embrynoic exposure down | ENSGMOG00000008774 | sp|Q80SX5|OTOP2_MOUSE Otopetrin-2 OS=Mus musculus GN=Otop2 PE=2 SV=1" | E2 | *otop2* | PMID: 18254951 |
| Embrynoic exposure down | ENSGMOG00000015313 | sp|Q3U962|CO5A2_MOUSE Collagen alpha-2(V) chain OS=Mus musculus GN=Col5a2 PE=1 SV=1" | E2 | *co5a2* | RefSeq |
| Embrynoic exposure down | ENSGMOG00000001159 | sp|P18910|ANPRA_RAT Atrial natriuretic peptide receptor 1 OS=Rattus norvegicus GN=Npr1 PE=1 SV=1" | E2 | *anpra* | PMID: 26215453 |
| Embrynoic exposure down | ENSGMOG00000002406 | sp|Q9EPW4|CLC3A_MOUSE C-type lectin domain family 3 member A OS=Mus musculus GN=Clec3a PE=2 SV=1" | E2, E4 | *clc3a* | PMID: 10524194 |
| Embrynoic exposure down | ENSGMOG00000015105 | sp|P55088|AQP4_MOUSE Aquaporin-4 OS=Mus musculus GN=Aqp4 PE=2 SV=2" | E2. E3 | *aqp4* | PMID: 25257965 |
| Embrynoic exposure down | ENSGMOG00000019249 | sp|P16112|PGCA_HUMAN Aggrecan core protein OS=Homo sapiens GN=ACAN PE=1 SV=2" | E2. E3 | *pgca* | PMID: 22297263 |
| Embrynoic exposure down | ENSGMOG00000007081 | sp|D4A9R4|PRRT4_RAT Proline-rich transmembrane protein 4 OS=Rattus norvegicus GN=Prrt4 PE=4 SV=1" | E3 | *prrt4* | PMID: 25314575 |
| Embrynoic exposure down | ENSGMOG00000001981 | sp|Q14149|MORC3_HUMAN MORC family CW-type zinc finger protein 3 OS=Homo sapiens GN=MORC3 PE=1 SV=3" | E3 | *morc3* | PMID: 20501696, PMID: 22258483, PMID: 11927593 |
| Embrynoic exposure down | ENSGMOG00000020201 | sp|P02264|H2A_ONCMY Histone H2A OS=Oncorhynchus mykiss PE=1 SV=2" | E3 | *h2a* | RefSeq |
| Embrynoic exposure down | ENSGMOG00000010995 | sp|P19540|FA9_CANFA Coagulation factor IX OS=Canis familiaris GN=F9 PE=1 SV=1" | E3 | *fa9* | RefSeq |
| Embrynoic exposure down | ENSGMOG00000017977 | sp|Q9N126|RDH8_BOVIN Retinol dehydrogenase 8 OS=Bos taurus GN=RDH8 PE=1 SV=1" | E3 | *rdh8* | PMID: 22621924 |
| Embrynoic exposure down | ENSGMOG00000017767 | sp|Q8WTQ7|GRK7_HUMAN G protein-coupled receptor kinase 7 OS=Homo sapiens GN=GRK7 PE=2 SV=1" | E3 | *grk7* | PMID:22183412 |
| Embrynoic exposure down | ENSGMOG00000018030 | sp|P08699|LEG3_RAT Galectin-3 OS=Rattus norvegicus GN=Lgals3 PE=1 SV=4" | E3 | *leg3* | PMID: 26313861 |
| Embrynoic exposure down | ENSGMOG00000020539 |  | E3 |  | none available |
| Embrynoic exposure down | ENSGMOG00000001173 | sp|P32847|B3AT_ONCMY Band 3 anion exchange protein OS=Oncorhynchus mykiss GN=slc4a1 PE=2 SV=2" | E3, E4 | *b3at* | PMID: 23846695 |
| Embrynoic exposure down | ENSGMOG00000010305 | sp|P79147|GBB3_CANFA Guanine nucleotide-binding protein G(I)/G(S)/G(T) subunit beta-3 OS=Canis familiaris GN=GNB3 PE=2 SV=1" | E4 | *gbb3* | PMID: 25806532 |
| Embrynoic exposure down | ENSGMOG00000001694 | sp|Q54HH2|SRR_DICDI Probable serine racemase OS=Dictyostelium discoideum GN=srr PE=3 SV=1" | E4 | *srr* | PMID: 17697119 |
| Embrynoic exposure down | ENSGMOG00000016535 | sp|P08938|PURP_CHICK Purpurin OS=Gallus gallus PE=1 SV=1" | E4 | *purp* | PMID: 24928393 |
| Embrynoic exposure down | ENSGMOG00000009354 | sp|P10039|TENA_CHICK Tenascin OS=Gallus gallus GN=TNC PE=1 SV=2" | E4 | *tena* | PMID: 25557620 |
| Embrynoic exposure down | ENSGMOG00000010355 | sp|Q90850|HIC1_CHICK Hypermethylated in cancer 1 protein (Fragment) OS=Gallus gallus GN=HIC1 PE=2 SV=2" | E4 | *hic1* | PMID: 15231840 |
| Embrynoic exposure down | ENSGMOG00000020478 | sp|P30872|SSR1_HUMAN Somatostatin receptor type 1 OS=Homo sapiens GN=SSTR1 PE=1 SV=1" | E4 | *ssr1* | PMID: 21912697 |
| Embrynoic exposure down | ENSGMOG00000010304 | sp|O35632|HYAL2_MOUSE Hyaluronidase-2 OS=Mus musculus GN=Hyal2 PE=1 SV=2" | E4 | *hyal2* | RefSeq |
| Embrynoic exposure down | ENSGMOG00000006689 | sp|Q8BHI9|NIM1_MOUSE Serine/threonine-protein kinase NIM1 OS=Mus musculus GN=Nim1 PE=2 SV=1" | E4, E6 | *nim1* | not much information |
| Embrynoic exposure down | ENSGMOG00000005897 | sp|Q15835|RK_HUMAN Rhodopsin kinase OS=Homo sapiens GN=GRK1 PE=1 SV=1" | E5 | *rk* | PMID: 22183412 |
| Embrynoic exposure down | ENSGMOG00000019360 | sp|Q9PT84|KCNH2_CHICK Potassium voltage-gated channel subfamily H member 2 (Fragment) OS=Gallus gallus GN=KCNH2 PE=2 SV=1" | E5 | *kcnh2* | PMID: 17592134 |
| Embrynoic exposure down | ENSGMOG00000015897 | sp|Q9UP65|PA24C_HUMAN Cytosolic phospholipase A2 gamma OS=Homo sapiens GN=PLA2G4C PE=1 SV=2" | E5 | *pa24c* | PMID: 22108055 |
| Embrynoic exposure down | ENSGMOG00000010281 | sp|O94856|NFASC_HUMAN Neurofascin OS=Homo sapiens GN=NFASC PE=1 SV=4" | E5 | *nfasc* | PMID: 25653379 |
| Embrynoic exposure down | ENSGMOG00000003765 | sp|Q9R0X5|RPGR_MOUSE X-linked retinitis pigmentosa GTPase regulator OS=Mus musculus GN=Rpgr PE=1 SV=1" | E5 | *rpgr* | PMID: 26124960 |
| Embrynoic exposure down | ENSGMOG00000004352 | sp|Q5EBI0|S2610_MOUSE Solute carrier family 26 member 10 OS=Mus musculus GN=Slc26a10 PE=2 SV=1" | E5 | *s2610* | PMID: 17693766 |
| Embrynoic exposure down | ENSGMOG00000008734 | sp|P0CG50|UBC_MOUSE Polyubiquitin-C OS=Mus musculus GN=Ubc PE=1 SV=1" | E5 | *ubc* | PMID: 26317694 |
| Embrynoic exposure down | ENSGMOG00000019011 | sp|Q15818|NPTX1_HUMAN Neuronal pentraxin-1 OS=Homo sapiens GN=NPTX1 PE=2 SV=2" | E5 | *nptx1* | PMID: 25646694, PMID: 24529709 |
| Embrynoic exposure down | ENSGMOG00000006409 | sp|P27463|AL1A1_CHICK Retinal dehydrogenase 1 OS=Gallus gallus GN=ALDH1A1 PE=2 SV=1" | E5 | *al1a1* | PMID: 26005920 |
| Embrynoic exposure down | ENSGMOG00000007048 |  | E5, E6 |  | none available |
| Embrynoic exposure down | ENSGMOG00000008229 |  | E6 |  | none available |
| Embrynoic exposure down | ENSGMOG00000019689 | sp|P21857|UTS2_PLAFE Urotensin-2 (Fragments) OS=Platichthys flesus PE=1 SV=1" | E6 | *uts2* | RefSeq |
| Embrynoic exposure down | ENSGMOG00000012223 | sp|Q28GB8|NODAL_XENTR Nodal homolog OS=Xenopus tropicalis GN=nodal PE=2 SV=1" | E6 | *nodal* | PMID: 26123688 |
| Embrynoic exposure down | ENSGMOG00000013576 | sp|Q567I9|CB5D1_DANRE Cytochrome b5 domain-containing protein 1 OS=Danio rerio GN=cyb5d1 PE=2 SV=1" | E6 | *cb5d1* | A growing family of cytochrome b5-domain fusion proteins Johnathan A. Napiera et. al 1999 |
| Embrynoic exposure down | ENSGMOG00000017329 | sp|Q9Y3Q4|HCN4_HUMAN Potassium/sodium hyperpolarization-activated cyclic nucleotide-gated channel 4 OS=Homo sapiens GN=HCN4 PE=1 SV=1" | E6 | *hcn4* | PMID: 25145517 |
| Embrynoic exposure down | ENSGMOG00000016465 | sp|A8MXD5|GRCR1_HUMAN Glutaredoxin domain-containing cysteine-rich protein 1 OS=Homo sapiens GN=GRXCR1 PE=1 SV=1" | E6 | *grcr1* | PMID: 20137774 |
| Embrynoic exposure down | ENSGMOG00000008531 | sp|P00787|CATB_RAT Cathepsin B OS=Rattus norvegicus GN=Ctsb PE=1 SV=2" | E6 | *catb* | PMID: 11439329 |
| Embrynoic exposure down | ENSGMOG00000016303 | sp|Q8N5S9|KKCC1_HUMAN Calcium/calmodulin-dependent protein kinase kinase 1 OS=Homo sapiens GN=CAMKK1 PE=1 SV=2" | E6 | *kkcc1* | PMID: 19633294, PMID: 15150258, PMID: 15591024 |
| Embryonic exposure up | ENSGMOG00000009403 | sp|P56701|PSMD2_BOVIN 26S proteasome non-ATPase regulatory subunit 2 OS=Bos taurus GN=PSMD2 PE=1 SV=2" | E1 | *psmd2* | PMID: 15122206 |
| Embryonic exposure up | ENSGMOG00000009114 | sp|A9YTQ3|AHRR_HUMAN Aryl hydrocarbon receptor repressor OS=Homo sapiens GN=AHRR PE=2 SV=3" | E2 | *ahrr* | PMID: 18848529 |
| Embryonic exposure up | ENSGMOG00000011298 | sp|Q9ESN4|C1QL3_MOUSE Complement C1q-like protein 3 OS=Mus musculus GN=C1ql3 PE=2 SV=1" | E2 | *c1ql3* | PMID: 25752542; PMID: 21262840 |
| Embryonic exposure up | ENSGMOG00000013503 | sp|Q8N8Z6|DCBD1_HUMAN Discoidin, CUB and LCCL domain-containing protein 1 OS=Homo sapiens GN=DCBLD1 PE=2 SV=2" | E2 | *dcbd1* | GeneCards |
| Embryonic exposure up | ENSGMOG00000017323 | sp|Q2T9U8|NMB_BOVIN Neuromedin-B OS=Bos taurus GN=NMB PE=2 SV=1" | E2 | *nmb* | PMID: 25796599 |
| Embryonic exposure up | ENSGMOG00000007937 | sp|Q7T2D1|RD10B_DANRE Retinol dehydrogenase 10-B OS=Danio rerio GN=rdh10b PE=2 SV=2" | E2 | *rd10b* | PMID: 19141675 |
| Embryonic exposure up | ENSGMOG00000005841 | sp|Q9PWA1|RGS20_CHICK Regulator of G-protein signaling 20 OS=Gallus gallus GN=RGS20 PE=2 SV=1" | E2 | *rgs20* | PMID: 18407463 |
| Embryonic exposure up | ENSGMOG00000000322 | sp|Q6UXH8|CCBE1_HUMAN Collagen and calcium-binding EGF domain-containing protein 1 OS=Homo sapiens GN=CCBE1 PE=1 SV=1" | E2, E4 | *ccbe1* | PMID: 25545279, PMID: 25814692 |
| Embryonic exposure up | ENSGMOG00000000318 | sp|O42430|CP1A1_LIMLI Cytochrome P450 1A1 OS=Limanda limanda GN=cyp1a1 PE=2 SV=1" | E1, E2, E3, E4 | *cp1a1* | PMID: 1594670 |
| Embryonic exposure up | ENSGMOG00000020141 | sp|Q16678|CP1B1_HUMAN Cytochrome P450 1B1 OS=Homo sapiens GN=CYP1B1 PE=1 SV=2" | E2, E3, E4 | *cp1b1* | PMID: 25994265 |
| Embryonic exposure up | ENSGMOG00000019790 | sp|Q16678|CP1B1_HUMAN Cytochrome P450 1B1 OS=Homo sapiens GN=CYP1B1 PE=1 SV=2" | E2, E3, E4 | *cp1b1* | PMID: 25994265 |
| Embryonic exposure up | ENSGMOG00000006436 | sp|P11678|PERE_HUMAN Eosinophil peroxidase OS=Homo sapiens GN=EPX PE=1 SV=2" | E3 | *pere* | PMID: 24937179 |
| Embryonic exposure up | ENSGMOG00000007034 | sp|P08317|IL8_CHICK Interleukin-8 OS=Gallus gallus GN=IL8 PE=2 SV=1" | E3 | *il8* | PMID: 19617600, PMID: 25593029 |
| Embryonic exposure up | ENSGMOG00000015391 | sp|Q5BLE8|RETST_DANRE Putative all-trans-retinol 13,14-reductase OS=Danio rerio GN=retsat PE=2 SV=1" | E3 | *retst* | PMID: 19139408 |
| Embryonic exposure up | ENSGMOG00000020289 | sp|Q91233|HSP70_ONCTS Heat shock 70 kDa protein OS=Oncorhynchus tschawytscha GN=hsp70 PE=2 SV=1" | E3, E4 | *hsp70* | PMID: 25715055, PMID: 26118968 |
| Embryonic exposure up | ENSGMOG00000005408 | sp|P70412|CUZD1_MOUSE CUB and zona pellucida-like domain-containing protein 1 OS=Mus musculus GN=Cuzd1 PE=2 SV=2" | E3, E4 | *cuzd1* | PMID: 23710207 |
| Embryonic exposure up | ENSGMOG00000020504 | sp|Q91233|HSP70_ONCTS Heat shock 70 kDa protein OS=Oncorhynchus tschawytscha GN=hsp70 PE=2 SV=1" | E3, E4, E5 | *hsp70* | PMID: 25715055, PMID: 26118968 |
| Embryonic exposure up | ENSGMOG00000020073 | sp|Q9I8F9|HSP71_ORYLA Heat shock 70 kDa protein 1 OS=Oryzias latipes PE=3 SV=1" | E3, E4, E5 | *hsp71* | PMID: 23555724 |
| Embryonic exposure up | ENSGMOG00000008237 | sp|Q5EGZ1|ACE2_RAT Angiotensin-converting enzyme 2 OS=Rattus norvegicus GN=Ace2 PE=1 SV=1" | E4 | *ace2* | PMID: 25519733 |
| Embryonic exposure up | ENSGMOG00000016625 | sp|P08317|IL8_CHICK Interleukin-8 OS=Gallus gallus GN=IL8 PE=2 SV=1" | E4 | *il8* | PMID: 19617600, PMID: 25593029 |
| Embryonic exposure up | ENSGMOG00000001398 | sp|P00593|PA21B_BOVIN Phospholipase A2 OS=Bos taurus GN=PLA2G1B PE=1 SV=2" | E4, E6 | *pa21b* | RefSeq |
| Embryonic exposure up | ENSGMOG00000017237 | sp|P06596|PA21B_CANFA Phospholipase A2 OS=Canis familiaris GN=PLA2G1B PE=2 SV=1" | E5 | *pa21b* | RefSeq |
| Embryonic exposure up | ENSGMOG00000009696 | sp|Q8N4G2|ARL14_HUMAN ADP-ribosylation factor-like protein 14 OS=Homo sapiens GN=ARL14 PE=1 SV=2" | E5 | *arl14* | PMID: 21458045 |
| Embryonic exposure up | ENSGMOG00000007133 | sp|Q95KN1|CLUL1_CANFA Clusterin-like protein 1 OS=Canis familiaris GN=CLUL1 PE=1 SV=1" | E5 | *clul1* | PMID: 14507903 |
| Embryonic exposure up | ENSGMOG00000013161 | sp|Q7SIG3|ELA1_SALSA Elastase-1 OS=Salmo salar PE=1 SV=1" | E5 | *ela1* | RefSeq |
| Embryonic exposure up | ENSGMOG00000016599 | sp|O00178|GTPB1_HUMAN GTP-binding protein 1 OS=Homo sapiens GN=GTPBP1 PE=1 SV=3" | E5 | *gtpb1* | PMID: 10938096 |
| Embryonic exposure up | ENSGMOG00000020457 | sp|P14222|PERF_HUMAN Perforin-1 OS=Homo sapiens GN=PRF1 PE=1 SV=1" | E5 | *perf* | PMID: 19446473, PMID: 20536555 |
| Embryonic exposure up | ENSGMOG00000010207 |  | E5 |  | none available |
| Embryonic exposure up | ENSGMOG00000020456 | sp|P14222|PERF_HUMAN Perforin-1 OS=Homo sapiens GN=PRF1 PE=1 SV=1" | E5, E6 | *perf* | PMID: 19446473, PMID: 20536555 |
| Embryonic exposure up | ENSGMOG00000020481 | sp|P31580|HCE1_ORYLA High choriolytic enzyme 1 OS=Oryzias latipes GN=hcea PE=1 SV=1" | E6 | *hce1* | PMID: 2656664 |
| Embryonic exposure up | ENSGMOG00000015821 | sp|P31580|HCE1_ORYLA High choriolytic enzyme 1 OS=Oryzias latipes GN=hcea PE=1 SV=1" | E6 | *hce1* | PMID: 2656664 |
| Embryonic exposure up | ENSGMOG00000015824 | sp|P31580|HCE1_ORYLA High choriolytic enzyme 1 OS=Oryzias latipes GN=hcea PE=1 SV=1" | E6 | *hce1* | PMID: 2656664 |
| Embryonic exposure up | ENSGMOG00000014439 | sp|Q10835|MMP13_XENLA Collagenase 3 (Fragment) OS=Xenopus laevis GN=mmp13 PE=2 SV=1" | E6 | *mmp13* | PMID: 21094261 |
| Embryonic exposure up | ENSGMOG00000005179 | sp|P43883|PLIN2_MOUSE Perilipin-2 OS=Mus musculus GN=Plin2 PE=2 SV=1" | E6 | *plin2* | PMID: 23977392 |
| Embryonic exposure up | ENSGMOG00000004390 | sp|Q8CH34|ZP4_RAT Zona pellucida sperm-binding protein 4 OS=Rattus norvegicus GN=Zp4 PE=2 SV=1" | E6 | *zp4* | PMID: 12968942 |
| Embryonic exposure up | ENSGMOG00000005387 |  | E6 |  | none available |
| Larval exposure down | ENSGMOG00000007041 | sp|Q561R8|FA26F_RAT Protein FAM26F OS=Rattus norvegicus GN=Fam26f PE=2 SV=1" | L1 | *fa26f* | PMID: 25320282 |
| Larval exposure down | ENSGMOG00000008942 | sp|P36371|TAP2_MOUSE Antigen peptide transporter 2 OS=Mus musculus GN=Tap2 PE=2 SV=1" | L1 | *tap2* | RefSeq |
| Larval exposure down | ENSGMOG00000017132 | sp|Q03064|MOT1_CRILO Monocarboxylate transporter 1 OS=Cricetulus longicaudatus GN=SLC16A1 PE=1 SV=1" | L1 | *mot1* | PMID: 23384686 |
| Larval exposure down | ENSGMOG00000019355 | sp|Q95LS7|CCD96_MACFA Coiled-coil domain-containing protein 96 OS=Macaca fascicularis GN=CCDC96 PE=2 SV=1" | L1 | *ccd96* | PMID: 25074808 |
| Larval exposure down | ENSGMOG00000011116 | sp|Q03692|COAA1_HUMAN Collagen alpha-1(X) chain OS=Homo sapiens GN=COL10A1 PE=1 SV=2" | L3 | *coaa1* | PMID: 25321476 |
| Larval exposure down | ENSGMOG00000001850 | sp|Q8CFS6|KCNV2_MOUSE Potassium voltage-gated channel subfamily V member 2 OS=Mus musculus GN=Kcnv2 PE=2 SV=1" | L3 | *kcnv2* | RefSeq |
| Larval exposure down | ENSGMOG00000003446 | sp|O95428|PPN_HUMAN Papilin OS=Homo sapiens GN=PAPLN PE=2 SV=4" | L3 | *ppn* | PMID: 11076767 |
| Larval exposure down | ENSGMOG00000004794 | sp|Q90257|ES1_DANRE ES1 protein, mitochondrial OS=Danio rerio GN=es1 PE=2 SV=1" | L3 | *es1* | PMID: 9150728, RefSeq |
| Larval exposure down | ENSGMOG00000005302 | sp|Q61830|MRC1_MOUSE Macrophage mannose receptor 1 OS=Mus musculus GN=Mrc1 PE=1 SV=1" | L3 | *mrc1* | PMID: 23345393 |
| Larval exposure down | ENSGMOG00000005551 | sp|Q9H2D6|TARA_HUMAN TRIO and F-actin-binding protein OS=Homo sapiens GN=TRIOBP PE=1 SV=3" | L3 | *tara* | RefSeq |
| Larval exposure down | ENSGMOG00000005738 | sp|Q90257|ES1_DANRE ES1 protein, mitochondrial OS=Danio rerio GN=es1 PE=2 SV=1" | L3 | *es1* | PMID: 9150728, RefSeq |
| Larval exposure down | ENSGMOG00000006202 | sp|Q6ZUJ8|BCAP_HUMAN Phosphoinositide 3-kinase adapter protein 1 OS=Homo sapiens GN=PIK3AP1 PE=1 SV=2" | L3 | *bcap* | PMID: 23913047, PMID: 22187460 |
| Larval exposure down | ENSGMOG00000007538 | sp|Q8N895|ZN366_HUMAN Zinc finger protein 366 OS=Homo sapiens GN=ZNF366 PE=2 SV=1" | L3 | *zn366* | PMID: 22615205 |
| Larval exposure down | ENSGMOG00000020539 |  | L3 |  | none available |
| Larval exposure down | ENSGMOG00000006012 | sp|P45831|Y1136_MYCLE Uncharacterized protein ML1136 OS=Mycobacterium leprae GN=ML1136 PE=3 SV=1" | L4 | *y1136* | none available |
| Larval exposure down | ENSGMOG00000011784 | sp|Q9BXJ0|C1QT5_HUMAN Complement C1q tumor necrosis factor-related protein 5 OS=Homo sapiens GN=C1QTNF5 PE=1 SV=1" | L4 | *c1qt5* | RefSeq |
| Larval exposure down | ENSGMOG00000016555 | sp|Q6UWY5|OLFL1_HUMAN Olfactomedin-like protein 1 OS=Homo sapiens GN=OLFML1 PE=1 SV=2" | L4 | *olfl1* | PMID: 18667622 |
| Larval exposure down | ENSGMOG00000017911 | sp|P97329|KI20A_MOUSE Kinesin-like protein KIF20A OS=Mus musculus GN=Kif20a PE=1 SV=1" | L4 | *ki20a* | PMID: 25953216 |
| Larval exposure down | ENSGMOG00000018575 | sp|P32418|NAC1_HUMAN Sodium/calcium exchanger 1 OS=Homo sapiens GN=SLC8A1 PE=1 SV=3" | L4 | *nac1* | PMID: 25481039 |
| Larval exposure down | ENSGMOG00000014657 | sp|P11678|PERE_HUMAN Eosinophil peroxidase OS=Homo sapiens GN=EPX PE=1 SV=2" | L4, L5 | *pere* | RefSeq |
| Larval exposure down | ENSGMOG00000016666 | sp|Q66S03|NATTE_THANI Nattectin OS=Thalassophryne nattereri PE=2 SV=1" | L4, L5 | *natte* | PMID: 22940186, PMID: 24397626 |
| Larval exposure down | ENSGMOG00000016698 | sp|P83514|SCAL1_STRCA Struthiocalcin-1 OS=Struthio camelus PE=1 SV=1" | L4, L5 | *scal1* | PMID: 25849392 |
| Larval exposure down | ENSGMOG00000000080 | sp|P40313|CTRL_HUMAN Chymotrypsin-like protease CTRL-1 OS=Homo sapiens GN=CTRL PE=2 SV=1" | L5 | *ctrl* | PMID: 19494363 |
| Larval exposure down | ENSGMOG00000001440 | sp|P08217|CEL2A_HUMAN Chymotrypsin-like elastase family member 2A OS=Homo sapiens GN=CELA2A PE=1 SV=1" | L5 | *cel2a* | PMID: 21117707 |
| Larval exposure down | ENSGMOG00000005387 |  | L5 |  | none available |
| Larval exposure down | ENSGMOG00000005408 | sp|P70412|CUZD1_MOUSE CUB and zona pellucida-like domain-containing protein 1 OS=Mus musculus GN=Cuzd1 PE=2 SV=2" | L5 | *cuzd1* | PMID: 23710207 |
| Larval exposure down | ENSGMOG00000010841 | sp|Q6R5N8|TLR13_MOUSE Toll-like receptor 13 OS=Mus musculus GN=Tlr13 PE=1 SV=1" | L5 | *tlr13* | PMID: 21131352 |
| Larval exposure down | ENSGMOG00000012948 | sp|P35031|TRY1_SALSA Trypsin-1 OS=Salmo salar PE=1 SV=1" | L5 | *try1* | PMID: 7556223 |
| Larval exposure down | ENSGMOG00000019803 | sp|B0FPE9|NALP3_MACMU NACHT, LRR and PYD domains-containing protein 3 OS=Macaca mulatta GN=NLRP3 PE=2 SV=1" | L5 | *nalp3* | PMID: 26306997 |
| Larval exposure up | ENSGMOG00000000322 | sp|Q6UXH8|CCBE1_HUMAN Collagen and calcium-binding EGF domain-containing protein 1 OS=Homo sapiens GN=CCBE1 PE=1 SV=1" | L1 | *ccbe1* | PMID: 25545279, PMID: 25814692 |
| Larval exposure up | ENSGMOG00000011420 |  | L1 |  | none available |
| Larval exposure up | ENSGMOG00000014566 | sp|P80646|CTRB_GADMO Chymotrypsin B OS=Gadus morhua PE=1 SV=1" | L1 | *ctrb* | PMID: 18211899 |
| Larval exposure up | ENSGMOG00000017272 | sp|P97814|PPIP1_MOUSE Proline-serine-threonine phosphatase-interacting protein 1 OS=Mus musculus GN=Pstpip1 PE=1 SV=1" | L1 | *ppip1* | RefSeq |
| Larval exposure up | ENSGMOG00000009114 | sp|A9YTQ3|AHRR_HUMAN Aryl hydrocarbon receptor repressor OS=Homo sapiens GN=AHRR PE=2 SV=3" | L1, L2 | *ahrr* | PMID: 18848529 |
| Larval exposure up | ENSGMOG00000019790 | sp|Q16678|CP1B1_HUMAN Cytochrome P450 1B1 OS=Homo sapiens GN=CYP1B1 PE=1 SV=2" | L1, L2, L3 | *cp1b1* | PMID: 25994265 |
| Larval exposure up | ENSGMOG00000006842 | sp|Q64429|CP1B1_MOUSE Cytochrome P450 1B1 OS=Mus musculus GN=Cyp1b1 PE=2 SV=2" | L1, L2, L3, L4 L5 | *cp1b1* | PMID: 25994265 |
| Larval exposure up | ENSGMOG00000014967 | sp|P79150|FGF7_CANFA Keratinocyte growth factor OS=Canis familiaris GN=FGF7 PE=2 SV=1" | L1, L2, L3, L4, L5 | *fgf7* | PMID: 16932896, PMID: 12865412, PMID: 15327889 |
| Larval exposure up | ENSGMOG00000020141 | sp|Q16678|CP1B1_HUMAN Cytochrome P450 1B1 OS=Homo sapiens GN=CYP1B1 PE=1 SV=2" | L1, L2, L3, L4, L5 | *cp1b1* | PMID: 25994265 |
| Larval exposure up | ENSGMOG00000000318 | sp|O42430|CP1A1_LIMLI Cytochrome P450 1A1 OS=Limanda limanda GN=cyp1a1 PE=2 SV=1" | L1, L2, L3, L4, L5, | *cp1a1* | PMID: 1594670 |
| Larval exposure up | ENSGMOG00000000218 | sp|Q9QUT0|RHAG_MOUSE Ammonium transporter Rh type A OS=Mus musculus GN=Rhag PE=2 SV=1" | L2 | *rhag* | PMID: 25730269 |
| Larval exposure up | ENSGMOG00000011415 | sp|P22388|EDN1_RAT Endothelin-1 OS=Rattus norvegicus GN=Edn1 PE=1 SV=2" | L2 | *edn1* | PMID: 25058015 |
| Larval exposure up | ENSGMOG00000017148 | sp|Q566V9|AUGNA_DANRE Augurin-A OS=Danio rerio GN=zgc:112443 PE=2 SV=1" | L2 | *augna* | PMID: 22396620 |
| Larval exposure up | ENSGMOG00000020500 | sp|Q9C009|FOXQ1_HUMAN Forkhead box protein Q1 OS=Homo sapiens GN=FOXQ1 PE=2 SV=2" | L2 | *foxq1* | PMID: 20055451, PMID: 25955104 |
| Larval exposure up | ENSGMOG00000006260 | sp|P22728|VISI_CHICK Visinin OS=Gallus gallus PE=2 SV=5" | L3 | *visi* | PMID: 22183412 |
| Larval exposure up | ENSGMOG00000006442 | sp|P09848|LPH_HUMAN Lactase-phlorizin hydrolase OS=Homo sapiens GN=LCT PE=1 SV=3" | L3 | *lph* | PMID: 24448642 |
| Larval exposure up | ENSGMOG00000007787 | sp|Q9PVE8|C330_FUNHE Cytochrome P450 3A30 OS=Fundulus heteroclitus GN=cyp3a30 PE=2 SV=2" | L3 | *c330* | PMID: 12842592 |
| Larval exposure up | ENSGMOG00000007792 | sp|O42563|CP3AR_ONCMY Cytochrome P450 3A27 OS=Oncorhynchus mykiss GN=cyp3a27 PE=2 SV=1" | L3 | *cp3ar* | PMID: 26306559 |
| Larval exposure up | ENSGMOG00000011319 | sp|Q552Z6|GTPA_DICDI GTP-binding protein A OS=Dictyostelium discoideum GN=gtpA PE=3 SV=1" | L3 | *gtpa* | very little information available |
| Larval exposure up | ENSGMOG00000000922 | sp|Q95125|TA2R_BOVIN Thromboxane A2 receptor OS=Bos taurus GN=TBXA2R PE=2 SV=2" | L4 | *ta2r* | PMID: 24996187 |
| Larval exposure up | ENSGMOG00000005816 | sp|Q8R116|NOTUM_MOUSE Protein notum homolog OS=Mus musculus GN=Notum PE=2 SV=2" | L4 | *notum* | PMID: 24675402 |
| Larval exposure up | ENSGMOG00000006924 | sp|Q5HZI2|C2C4C_MOUSE C2 calcium-dependent domain-containing protein 4C OS=Mus musculus GN=C2cd4cC2CD4 family PE=2 SV=1" | L4 | *c2c4c* | GeneCards |
| Larval exposure up | ENSGMOG00000018060 | sp|P91924|ARF_DUGJA ADP-ribosylation factor OS=Dugesia japonica PE=2 SV=3" | L4 | *arf* | PMID: 1618801 |
| Larval exposure up | ENSGMOG00000020134 |  | L4 |  | none available |
| Larval exposure up | ENSGMOG00000002291 | sp|A2BGD5|CACO1_DANRE Calcium-binding and coiled-coil domain-containing protein 1 OS=Danio rerio GN=calcoco1 PE=3 SV=1" | L4, L5 | *caco1* | GeneCards |
| Larval exposure up | ENSGMOG00000002660 | sp|Q1LYM6|KLH38_DANRE Kelch-like protein 38 OS=Danio rerio GN=klhl38 PE=4 SV=1" | L5 | *klh38* | PMID: 23676014 |
| Larval exposure up | ENSGMOG00000003852 | sp|Q9NY28|GALT8_HUMAN Probable polypeptide N-acetylgalactosaminyltransferase 8 OS=Homo sapiens GN=GALNT8 PE=2 SV=1" | L5 | *galt8* | PMID: 24038392 |
| Larval exposure up | ENSGMOG00000005404 | sp|Q9D513|CP073_MOUSE Uncharacterized protein C16orf73 homolog OS=Mus musculus PE=2 SV=2" | L5 | *cp073* | none available |
| Larval exposure up | ENSGMOG00000010009 | sp|Q8TAE7|KCNG3_HUMAN Potassium voltage-gated channel subfamily G member 3 OS=Homo sapiens GN=KCNG3 PE=2 SV=1" | L5 | *kcng3* | PMID: 23424202 |
| Larval exposure up | ENSGMOG00000012862 | sp|Q7TN88|PK1L2_MOUSE Polycystic kidney disease protein 1-like 2 OS=Mus musculus GN=Pkd1l2 PE=2 SV=1" | L5 | *pk1l2* | PMID: 24756726 |

Supplementary file 1E:

| **Cod ID** | **Swissprot annotation** | **SP ID** | **GB ID** | **FC E1 H/C** | **FC E2 H/C** | **FC E3 H/C** | **FC E4 H/C** | **FC E5 H/C** | **FC E6 H/C** |
| --- | --- | --- | --- | --- | --- | --- | --- | --- | --- |
| ENSGMOG00000012645 | sp|Q7TST9|TBX3_RAT T-box transcription factor TBX3 OS=Rattus norvegicus GN=Tbx3 PE=2 SV=1" | *tbx3* | *tbx3* | 0 | 0 | 0 | 0 | 8.0701 | 0 |
| ENSGMOG00000001718 | sp|Q805D8|ANF_TAKRU Natriuretic peptides A OS=Takifugu rubripes GN=nppa PE=2 SV=1" | *anf* | *nppa* | 0 | 0 | 0 | 0 | 0 | -2.298 |
| ENSGMOG00000015370 | sp|P42583|NKX25_XENLA Homeobox protein Nkx-2.5 OS=Xenopus laevis GN=nkx-2.5 PE=2 SV=1" | *nkx25* | *nkx-2.5* | 0 | 0 | 2.944 | 4.890 | 0 | 0 |
| ENSGMOG00000006621 | sp|O95393|BMP10_HUMAN Bone morphogenetic protein 10 OS=Homo sapiens GN=BMP10 PE=2 SV=1" | *bmp10* | *bmp10* | 0 | 4.086 | 0 | 0 | 0 | 0 |

Supplementary file 1F

| **Cod gene ID** | **Swissprot annotation** | **SP ID** | **GB ID** | **Gene category** | Ref |
| --- | --- | --- | --- | --- | --- |
| ENSGMOG00000019454 | sp|Q61086|FZD3_MOUSE Frizzled-3 OS=Mus musculus GN=Fzd3 PE=1 SV=1" | *fzd3* | *fzd3* | craniofacial development | PMID: 19941846 |
| ENSGMOG00000005808 | sp|Q8WMU5|FZD6_CANFA Frizzled-6 OS=Canis familiaris GN=FZD6 PE=2 SV=1" | *fzd6* | *fzd6* | craniofacial development | PMID: 19941846 |
| ENSGMOG00000013652 | sp|Q9PUK8|FZD7A_XENLA Frizzled-7-A OS=Xenopus laevis GN=fzd7-a PE=1 SV=2" | *fzd7a* | *fzd7-a* | craniofacial development | PMID: 19941846 |
| ENSGMOG00000006715 | sp|Q9PUK8|FZD7A_XENLA Frizzled-7-A OS=Xenopus laevis GN=fzd7-a PE=1 SV=2" | *fzd7a* | *fzd7-a* | craniofacial development | PMID: 19941846 |
| ENSGMOG00000007975 | sp|Q91690|GLI1_XENLA Zinc finger protein GLI1 (Fragment) OS=Xenopus laevis GN=gli1 PE=2 SV=2" | *gli1* | *gli1* | craniofacial development | PMID: 19941846 |
| ENSGMOG00000010335 | sp|Q0VGT2|GLI2_MOUSE Zinc finger protein GLI2 OS=Mus musculus GN=Gli2 PE=1 SV=1" | *gli2* | *gli2* | craniofacial development | PMID: 19941846, PMID: 23725801 |
| ENSGMOG00000006713 | sp|Q9IA31|GLI3_CHICK Transcriptional activator GLI3 (Fragment) OS=Gallus gallus GN=GLI3 PE=1 SV=1" | *gli3* | *gli3* | craniofacial development | PMID: 19941846, PMID: 23725801 |
| ENSGMOG00000012756 | sp|Q96NR3|PTHD1_HUMAN Patched domain-containing protein 1 OS=Homo sapiens GN=PTCHD1 PE=2 SV=2" | *pthd1* | *ptchd1* | craniofacial development | PMID: 19941846, PMID: 23725801 |
| ENSGMOG00000009077 | sp|Q5RIV7|PTHD1_DANRE Patched domain-containing protein 1 OS=Danio rerio GN=ptchd1 PE=3 SV=1" | *pthd1* | *ptchd1* | craniofacial development | PMID: 19941846, PMID: 23725801 |
| ENSGMOG00000015883 | sp|O42224|SMO_CHICK Smoothened homolog (Fragment) OS=Gallus gallus GN=SMO PE=2 SV=1" | *smo* | *smo* | craniofacial development | PMID: 19941846, PMID: 23725801 |
| ENSGMOG00000012157 | sp|P49339|WNT11_CHICK Protein Wnt-11 OS=Gallus gallus GN=WNT11 PE=2 SV=1" | *wnt11* | *wnt11* | craniofacial development | PMID: 19941846 |
| ENSGMOG00000011204 | sp|Q27Q52|WNT5A_RABIT Protein Wnt-5a OS=Oryctolagus cuniculus GN=WNT5A PE=2 SV=1" | *wnt5a* | *wnt5a* | craniofacial development | PMID: 19941846 |
| ENSGMOG00000005285 | sp|O14905|WNT9B_HUMAN Protein Wnt-9b OS=Homo sapiens GN=WNT9B PE=1 SV=3" | *wnt9b* | *wnt9b* | craniofacial development | PMID: 19941846 |
| ENSGMOG00000005048 | sp|Q92008|SHH_DANRE Sonic hedgehog protein A OS=Danio rerio GN=shha PE=1 SV=1" | *shh* | *shha* | craniofacial development | PMID: 19941846, PMID: 23725801, PMID: 16049113 |
| ENSGMOG00000011088 | sp|Q804S2|BMP2_TETNG Bone morphogenetic protein 2 OS=Tetraodon nigroviridis GN=bmp2 PE=3 SV=2" | *bmp2* | *bmp2* | craniofacial development | PMID: 20663816, PMID: 19941846 |
| ENSGMOG00000012485 | sp|P12643|BMP2_HUMAN Bone morphogenetic protein 2 OS=Homo sapiens GN=BMP2 PE=1 SV=1" | *bmp2* | *bmp2* | craniofacial development | PMID: 20663816, PMID: 19941846 |
| ENSGMOG00000017744 | sp|Q804S2|BMP2_TETNG Bone morphogenetic protein 2 OS=Tetraodon nigroviridis GN=bmp2 PE=3 SV=2" | *bmp2* | *bmp2* | craniofacial development | PMID: 20663816, PMID: 19941846 |
| ENSGMOG00000011206 | sp|Q90752|BMP4_CHICK Bone morphogenetic protein 4 OS=Gallus gallus GN=BMP4 PE=2 SV=1" | *bmp4* | *bmp4* | craniofacial development | PMID: 20663816, PMID: 19941846 |
| ENSGMOG00000007362 | sp|P70492|FGF10_RAT Fibroblast growth factor 10 OS=Rattus norvegicus GN=Fgf10 PE=2 SV=1" | *fgf10* | *fgf10* | craniofacial - palate | PMID: 20663816, PMID: 19941846, PMID: 25564621 |
| ENSGMOG00000011825 | sp|P70492|FGF10_RAT Fibroblast growth factor 10 OS=Rattus norvegicus GN=Fgf10 PE=2 SV=1" | *fgf10* | *fgf10* | craniofacial - palate | PMID: 20663816, PMID: 19941846, PMID: 25564621 |
| ENSGMOG00000016405 | sp|O35565|FGF10_MOUSE Fibroblast growth factor 10 OS=Mus musculus GN=Fgf10 PE=2 SV=1" | *fgf10* | *fgf10* | craniofacial - palate | PMID: 20663816, PMID: 19941846, PMID: 25564621 |
| ENSGMOG00000013688 | sp|Q9UPM6|LHX6_HUMAN LIM/homeobox protein Lhx6 OS=Homo sapiens GN=LHX6 PE=2 SV=2" | *lhx6* | *lhx6* | craniofacial - palate | PMID: 20663816, PMID: 22016187 |
| ENSGMOG00000016580 | sp|Q9UPM6|LHX6_HUMAN LIM/homeobox protein Lhx6 OS=Homo sapiens GN=LHX6 PE=2 SV=2" | *lhx6* | *lhx6* | craniofacial - palate | PMID: 20663816, PMID: 22016187 |
| ENSGMOG00000016250 | sp|Q68G74|LHX8_HUMAN LIM/homeobox protein Lhx8 OS=Homo sapiens GN=LHX8 PE=2 SV=2" | *lhx8* | *lhx8* | craniofacial - palate | PMID: 20663816, PMID: 22016187 |
| ENSGMOG00000018358 | sp|Q68G74|LHX8_HUMAN LIM/homeobox protein Lhx8 OS=Homo sapiens GN=LHX8 PE=2 SV=2" | *lhx8* | *lhx8* | craniofacial - palate | PMID: 20663816, PMID: 22016187 |
| ENSGMOG00000009076 | sp|Q5XJQ7|OSR1_DANRE Protein odd-skipped-related 1 OS=Danio rerio GN=osr1 PE=2 SV=1" | *osr1* | *osr1* | craniofacial - palate | PMID: 22016187 |
| ENSGMOG00000016368 | sp|Q32NK7|OSR2A_XENLA Protein odd-skipped-related 2-A OS=Xenopus laevis GN=osr2-a PE=2 SV=1" | *osr2a* | *osr2-a* | craniofacial - palate | PMID: 22016187 |
| ENSGMOG00000009128 | sp|Q2VL56|PAX9_SAGOE Paired box protein Pax-9 OS=Saguinus oedipus GN=PAX9 PE=3 SV=1" | *pax9* | *pax9* | craniofacial - palate | PMID: 22016187 |
| ENSGMOG00000005788 | sp|Q9W7E7|SMAD5_DANRE Mothers against decapentaplegic homolog 5 OS=Danio rerio GN=smad5 PE=2 SV=1" | *smad5* | *smad5* | craniofacial - palate | PMID: 22016187 |
| ENSGMOG00000008837 | sp|P17247|TGFB2_XENLA Transforming growth factor beta-2 OS=Xenopus laevis GN=tgfb2 PE=1 SV=1" | *tgfb2* | *tgfb2* | craniofacial - palate | PMID: 22016187 |
| ENSGMOG00000017371 | sp|P27090|TGFB2_MOUSE Transforming growth factor beta-2 OS=Mus musculus GN=Tgfb2 PE=2 SV=1" | *tgfb2* | *tgfb2* | craniofacial - palate | PMID: 22016187 |
| ENSGMOG00000015251 | sp|P10600|TGFB3_HUMAN Transforming growth factor beta-3 OS=Homo sapiens GN=TGFB3 PE=1 SV=1" | *tgfb3* | *tgfb3* | craniofacial - palate | PMID: 22016187 |
| ENSGMOG00000011125 | sp|P10600|TGFB3_HUMAN Transforming growth factor beta-3 OS=Homo sapiens GN=TGFB3 PE=1 SV=1" | *tgfb3* | *tgfb3* | craniofacial - palate | PMID: 22016187 |
| ENSGMOG00000005079 | sp|Q5E968|CATK_BOVIN Cathepsin K OS=Bos taurus GN=CTSK PE=2 SV=2" | *catk* | *ctsk* | Bone/cartilage maintenance | PMID: 26453494, PMID: 11031235, PMID: 14993931 |
| ENSGMOG00000015255 | sp|O60911|CATL2_HUMAN Cathepsin L2 OS=Homo sapiens GN=CTSL2 PE=1 SV=2" | *catl2* | *ctsl2* | Bone/cartilage maintenance | PMID: 26453494, PMID: 11031235 |
| ENSGMOG00000015290 | sp|P02457|CO1A1_CHICK Collagen alpha-1(I) chain OS=Gallus gallus GN=COL1A1 PE=1 SV=3" | *co1a1* | *col1a1* | Bone/cartilage maintenance | PMID: 26453494, PMID: 11031235 |
| ENSGMOG00000008472 | sp|P02457|CO1A1_CHICK Collagen alpha-1(I) chain OS=Gallus gallus GN=COL1A1 PE=1 SV=3" | *co1a1* | *col1a1* | Bone/cartilage maintenance | PMID: 26453494, PMID: 11031235 |
| ENSGMOG00000013455 | sp|O93484|CO1A2_ONCMY Collagen alpha-2(I) chain OS=Oncorhynchus mykiss GN=col1a2 PE=2 SV=2" | *co1a2* | *col1a2* | Bone/cartilage maintenance | PMID: 26453494, PMID: 11031235 |
| ENSGMOG00000019181 | sp|O93484|CO1A2_ONCMY Collagen alpha-2(I) chain OS=Oncorhynchus mykiss GN=col1a2 PE=2 SV=2" | *co1a2* | *col1a2* | Bone/cartilage maintenance | PMID: 26453494, PMID: 11031235 |
| ENSGMOG00000001436 | sp|P02459|CO2A1_BOVIN Collagen alpha-1(II) chain OS=Bos taurus GN=COL2A1 PE=1 SV=4" | *co2a1* | *col2a1* | Bone/cartilage maintenance | PMID: 26453494, PMID: 11031235 |
| ENSGMOG00000009692 | sp|P05539|CO2A1_RAT Collagen alpha-1(II) chain OS=Rattus norvegicus GN=Col2a1 PE=2 SV=2" | *co2a1* | *col2a1* | Bone/cartilage maintenance | PMID: 26453494, PMID: 11031235 |
| ENSGMOG00000000027 | sp|Q03692|COAA1_HUMAN Collagen alpha-1(X) chain OS=Homo sapiens GN=COL10A1 PE=1 SV=2" | *coaa1* | *col10a1* | Bone/cartilage maintenance | PMID: 26453494, PMID: 11031235 |
| ENSGMOG00000011116 | sp|Q03692|COAA1_HUMAN Collagen alpha-1(X) chain OS=Homo sapiens GN=COL10A1 PE=1 SV=2" | *coaa1* | *col10a1* | Bone/cartilage maintenance | PMID: 26453494, PMID: 11031235 |
| ENSGMOG00000001431 | sp|P41246|MMP9_RABIT Matrix metalloproteinase-9 OS=Oryctolagus cuniculus GN=MMP9 PE=2 SV=1" | *mmp9* | *mmp9* | Bone/cartilage maintenance | PMID: 26453494, PMID: 11031235, PMID: 7898050 |
| ENSGMOG00000012329 | sp|Q15063|POSTN_HUMAN Periostin OS=Homo sapiens GN=POSTN PE=1 SV=2" | *postn* | *postn* | Bone/cartilage maintenance | PMID: 24323465, PMID: 26453494, PMID: 11031235 |
| ENSGMOG00000001525 | sp|Q15063|POSTN_HUMAN Periostin OS=Homo sapiens GN=POSTN PE=1 SV=2" | *postn* | *postn* | Bone/cartilage maintenance | PMID: 24323465, PMID: 26453494, PMID: 11031235 |
| ENSGMOG00000002250 | sp|Q98875|DLX1A_DANRE Homeobox protein Dlx1a OS=Danio rerio GN=dlx1a PE=2 SV=1" | *dlx1a* | *dlx1a* | NCC identity | PMID: 20663816, PMID: 19941846 |
| ENSGMOG00000002233 | sp|P50574|DLX2A_DANRE Homeobox protein Dlx2a OS=Danio rerio GN=dlx2a PE=2 SV=1" | *dlx2a* | *dlx2a* | NCC identity | PMID: 20663816, PMID: 19941846 |
| ENSGMOG00000013639 | sp|P50576|DLX5A_DANRE Homeobox protein Dlx5a OS=Danio rerio GN=dlx5a PE=2 SV=1" | *dlx5a* | *dlx5a* | NCC identity | PMID: 20663816, PMID: 19941846 |
| ENSGMOG00000013631 | sp|Q98877|DLX6A_DANRE Homeobox protein Dlx6a OS=Danio rerio GN=dlx6a PE=2 SV=1" | *dlx6a* | *dlx6a* | NCC identity | PMID: 20663816, PMID: 19941846 |
| ENSGMOG00000011415 | sp|P22388|EDN1_RAT Endothelin-1 OS=Rattus norvegicus GN=Edn1 PE=1 SV=2" | *edn1* | *edn1* | NCC identity | PMID: 20663816, PMID: 19941846, PMID: 19017795, PMID: 11336493, PMID: 17358015 |
| ENSGMOG00000002010 | sp|Q95L55|EDNRA_SHEEP Endothelin-1 receptor OS=Ovis aries GN=EDNRA PE=2 SV=1" | *ednra* | *ednra* | NCC identity | PMID: 20663816, PMID: 19941846, PMID: 19017795 |
| ENSGMOG00000002220 | sp|P48802|FGF3_DANRE Fibroblast growth factor 3 OS=Danio rerio GN=fgf3 PE=1 SV=1" | *fgf3* | *fgf3* | NCC identity | PMID: 20663816, PMID: 19941846 |
| ENSGMOG00000015070 | sp|Q90722|FGF8_CHICK Fibroblast growth factor 8 OS=Gallus gallus GN=FGF8 PE=2 SV=1" | *fgf8* | *fgf8* | NCC identity | PMID: 20663816, PMID: 19941846 |
| ENSGMOG00000018978 | sp|P57102|HAND2_DANRE Heart- and neural crest derivatives-expressed protein 2 OS=Danio rerio GN=hand2 PE=2 SV=1" | *hand2* | *hand2* | NCC identity | PMID: 20663816, PMID: 19017795 |
| ENSGMOG00000009780 | sp|Q5R444|MEF2C_PONAB Myocyte-specific enhancer factor 2C OS=Pongo abelii GN=MEF2C PE=2 SV=1" | *mef2c* | *mef2c* | NCC identity | PMID: 20663816, PMID: 19941846 |
| ENSGMOG00000000565 | sp|Q5R444|MEF2C_PONAB Myocyte-specific enhancer factor 2C OS=Pongo abelii GN=MEF2C PE=2 SV=1" | *mef2c* | *mef2c* | NCC identity | PMID: 20663816, PMID: 19941846 |
| ENSGMOG00000018725 | sp|P78367|NKX32_HUMAN Homeobox protein Nkx-3,2 OS=Homo sapiens GN=NKX3-2 PE=2 SV=2" | *nkx32* | *nkx3-2* | NCC identity | PMID: 20663816, PMID: 19941846 |
| ENSGMOG00000015623 | sp|Q6DFF5|SOX9B_XENLA Transcription factor Sox-9-B OS=Xenopus laevis GN=sox9-b PE=2 SV=1" | *sox9b* | *sox9-b* | NCC identity | PMID: 20663816 |
| ENSGMOG00000009261 | sp|Q6DFF5|SOX9B_XENLA Transcription factor Sox-9-B OS=Xenopus laevis GN=sox9-b PE=2 SV=1" | *sox9b* | *sox9-b* | NCC identity | PMID: 20663816 |
| ENSGMOG00000009533 | sp|Q90Z00|FGR1A_DANRE Basic fibroblast growth factor receptor 1-A OS=Danio rerio GN=fgfr1a PE=1 SV=2" | *fgfr1a* | *fgfr1a* | NCC migration | PMID: 20663816 |
| ENSGMOG00000013834 | sp|Q1KL11|HXA2A_TAKRU Homeobox protein Hox-A2a OS=Takifugu rubripes GN=hoxa2a PE=3 SV=1" | *hxa2a* | *hoxa2a* | NCC migration | PMID: 20663816 |
| ENSGMOG00000012609 | sp|O35276|NRP2_RAT Neuropilin-2 OS=Rattus norvegicus GN=Nrp2 PE=2 SV=1" | *nrp2* | *nrp2* | NCC migration | PMID: 20663816 |
| ENSGMOG00000016020 | sp|O35276|NRP2_RAT Neuropilin-2 OS=Rattus norvegicus GN=Nrp2 PE=2 SV=1" | *nrp2* | *nrp2* | NCC migration | PMID: 20663816 |
| ENSGMOG00000013657 | sp|Q9UPW6|SATB2_HUMAN DNA-binding protein SATB2 OS=Homo sapiens GN=SATB2 PE=1 SV=2" | *satb2* | *satb2* | NCC migration | PMID: 20663816 |
| ENSGMOG00000006865 | sp|Q8VI24|SATB2_MOUSE DNA-binding protein SATB2 OS=Mus musculus GN=Satb2 PE=1 SV=1" | *satb2* | *satb2* | NCC migration | PMID: 20663816 |
| ENSGMOG00000019678 | sp|Q13275|SEM3F_HUMAN Semaphorin-3F OS=Homo sapiens GN=SEMA3F PE=2 SV=2" | *sem3f* | *sema3f* | NCC migration | PMID: 20663816 |
| ENSGMOG00000016462 | sp|Q13275|SEM3F_HUMAN Semaphorin-3F OS=Homo sapiens GN=SEMA3F PE=2 SV=2" | *sem3f* | *sema3f* | NCC migration | PMID: 20663816 |
| ENSGMOG00000001255 | sp|Q13275|SEM3F_HUMAN Semaphorin-3F OS=Homo sapiens GN=SEMA3F PE=2 SV=2" | *sem3f* | *sema3f* | NCC migration | PMID: 20663816 |
| ENSGMOG00000002430 | sp|Q13275|SEM3F_HUMAN Semaphorin-3F OS=Homo sapiens GN=SEMA3F PE=2 SV=2" | *sem3f* | *sema3f* | NCC migration | PMID: 20663816 |
| ENSGMOG00000016820 | sp|Q4LFA9|SEM3G_MOUSE Semaphorin-3G OS=Mus musculus GN=Sema3g PE=2 SV=1" | *sem3g* | *sema3g* | NCC migration | PMID: 20663816 |
| ENSGMOG00000018613 | sp|Q8AXX2|TBX1_DANRE T-box transcription factor TBX1 OS=Danio rerio GN=tbx1 PE=2 SV=1" | *tbx1* | *tbx1* | NCC migration | PMID: 20663816 |
| ENSGMOG00000002790 | sp|P13903|TWIST_XENLA Twist-related protein OS=Xenopus laevis GN=twist1 PE=2 SV=1" | *twist* | *twist1* | NCC migration | PMID: 20663816 |
| ENSGMOG00000003155 | sp|P13903|TWIST_XENLA Twist-related protein OS=Xenopus laevis GN=twist1 PE=2 SV=1" | *twist* | *twist1* | NCC migration | PMID: 20663816 |
| ENSGMOG00000013924 | sp|O95393|BMP10_HUMAN Bone morphogenetic protein 10 OS=Homo sapiens GN=BMP10 PE=2 SV=1" | *bmp10* | *bmp10* | cardiogenesis | PMID: 15073151, PMID: 22996691 |
| ENSGMOG00000005535 | sp|Q9R229|BMP10_MOUSE Bone morphogenetic protein 10 OS=Mus musculus GN=Bmp10 PE=2 SV=2" | *bmp10* | *bmp10* | cardiogenesis | PMID: 15073151, PMID: 22996691 |
| ENSGMOG00000006621 | sp|O95393|BMP10_HUMAN Bone morphogenetic protein 10 OS=Homo sapiens GN=BMP10 PE=2 SV=1" | *bmp10* | *bmp10* | cardiogenesis | PMID: 15073151, PMID: 22996691 |
| ENSGMOG00000011075 | sp|Q90953|CSPG2_CHICK Versican core protein OS=Gallus gallus GN=VCAN PE=2 SV=1" | *cspg2* | *vcan* | cardiogenesis | PMID: 22992950 |
| ENSGMOG00000016422 | sp|P13611|CSPG2_HUMAN Versican core protein OS=Homo sapiens GN=VCAN PE=1 SV=3" | *cspg2* | *vcan* | cardiogenesis | PMID: 22992950 |
| ENSGMOG00000003382 | sp|P10158|FOSL1_RAT Fos-related antigen 1 OS=Rattus norvegicus GN=Fosl1 PE=2 SV=1" | *fosl1* | *fosl1* | cardiogenesis | PMID: 18158353 |
| ENSGMOG00000003817 | sp|P43695|GAT5A_XENLA GATA-binding factor 5-A OS=Xenopus laevis GN=gata5-a PE=2 SV=1" | *gat5a* | *gata5-a* | cardiogenesis | PMID: 11909814, PMID: 24036209 |
| ENSGMOG00000011374 | sp|Q91678|GAT6A_XENLA GATA-binding factor 6-A OS=Xenopus laevis GN=gata6-a PE=2 SV=1" | *gat6a* | *gata6-a* | cardiogenesis | PMID: 11909814, PMID: 24036209 |
| ENSGMOG00000010832 | sp|Q91677|GATA4_XENLA Transcription factor GATA-4 OS=Xenopus laevis GN=gata4 PE=2 SV=1" | *gata4* | *gata4* | cardiogenesis | PMID: 11909814, PMID: 24036209 |
| ENSGMOG00000009538 | sp|Q90691|HAND1_CHICK Heart- and neural crest derivatives-expressed protein 1 OS=Gallus gallus GN=HAND1 PE=2 SV=1" | *hand1* | *hand1* | cardiogenesis | PMID: 11909814, PMID: 24036209 |
| ENSGMOG00000018978 | sp|P57102|HAND2_DANRE Heart- and neural crest derivatives-expressed protein 2 OS=Danio rerio GN=hand2 PE=2 SV=1" | *hand2* | *hand2* | cardiogenesis | PMID: 11909814, PMID: 24036209 |
| ENSGMOG00000002384 | sp|P70312|HAS2_MOUSE Hyaluronan synthase 2 OS=Mus musculus GN=Has2 PE=2 SV=3" | *has2* | *has2* | cardiogenesis | PMID: 18267096 |
| ENSGMOG00000015810 | sp|Q9UBP5|HEY2_HUMAN Hairy/enhancer-of-split related with YRPW motif protein 2 OS=Homo sapiens GN=HEY2 PE=1 SV=1" | *hey2* | *hey2* | cardiogenesis | PMID: 11909814 |
| ENSGMOG00000001799 | sp|O00292|LFTY2_HUMAN Left-right determination factor 2 OS=Homo sapiens GN=LEFTY2 PE=1 SV=2" | *lfty2* | *lefty2* | cardiogenesis | PMID: 18267096 |
| ENSGMOG00000015431 | sp|Q03414|MEF2A_XENLA Myocyte-specific enhancer factor 2A homolog OS=Xenopus laevis GN=mef2a PE=1 SV=2" | *mef2a* | *mef2a* | cardiogenesis | PMID: 24036209 |
| ENSGMOG00000015370 | sp|P42583|NKX25_XENLA Homeobox protein Nkx-2.5 OS=Xenopus laevis GN=nkx-2.5 PE=2 SV=1" | *nkx25* | *nkx-2.5* | cardiogenesis | PMID: 24036209 |
| ENSGMOG00000009138 | sp|O70584|NKX28_MOUSE Homeobox protein Nkx-2.8 OS=Mus musculus GN=Nkx2-8 PE=2 SV=1" | *nkx28* | *nkx2-8* | cardiogenesis | PMID: 19158954 |
| ENSGMOG00000010518 | sp|Q05199|NRG1_CHICK Pro-neuregulin-1. membrane-bound isoform OS=Gallus gallus GN=NRG1 PE=1 SV=1" | *nrg1* | *nrg1* | cardiogenesis | PMID: 15073151 |
| ENSGMOG00000002649 | sp|P51532|SMCA4_HUMAN Transcription activator BRG1 OS=Homo sapiens GN=SMARCA4 PE=1 SV=2" | *smca4* | *smarca4* | cardiogenesis | PMID: 18158353 |
| ENSGMOG00000005756 | sp|P51532|SMCA4_HUMAN Transcription activator BRG1 OS=Homo sapiens GN=SMARCA4 PE=1 SV=2" | *smca4* | *smarca4* | cardiogenesis | PMID: 18158353 |
| ENSGMOG00000012734 | sp|Q9I9K7|TBX20_DANRE T-box transcription factor TBX20 OS=Danio rerio GN=tbx20 PE=2 SV=1" | *tbx20* | *tbx20* | cardiogenesis | PMID: 22992950 |
| ENSGMOG00000009107 | sp|Q9PWE8|TBX5_CHICK T-box transcription factor TBX5 OS=Gallus gallus GN=TBX5 PE=2 SV=1" | *tbx5* | *tbx5* | cardiogenesis | PMID: 22974299 |
| ENSGMOG00000011740 | sp|Q9IAK8|TBX5A_DANRE T-box transcription factor TBX5-A OS=Danio rerio GN=tbx5a PE=2 SV=2" | *tbx5a* | *tbx5a* | cardiogenesis | PMID: 22974299 |
| ENSGMOG00000008837 | sp|P17247|TGFB2_XENLA Transforming growth factor beta-2 OS=Xenopus laevis GN=tgfb2 PE=1 SV=1" | *tgfb2* | *tgfb2* | cardiogenesis | PMID: 24036209 |
| ENSGMOG00000017371 | sp|P27090|TGFB2_MOUSE Transforming growth factor beta-2 OS=Mus musculus GN=Tgfb2 PE=2 SV=1" | *tgfb2* | *tgfb2* | cardiogenesis | PMID: 24036209 |
| ENSGMOG00000015251 | sp|P10600|TGFB3_HUMAN Transforming growth factor beta-3 OS=Homo sapiens GN=TGFB3 PE=1 SV=1" | *tgfb3* | *tgfb3* | cardiogenesis | PMID: 24036209 |
| ENSGMOG00000011125 | sp|P10600|TGFB3_HUMAN Transforming growth factor beta-3 OS=Homo sapiens GN=TGFB3 PE=1 SV=1" | *tgfb3* | *tgfb3* | cardiogenesis | PMID: 24036209 |
| ENSGMOG00000012157 | sp|P49339|WNT11_CHICK Protein Wnt-11 OS=Gallus gallus GN=WNT11 PE=2 SV=1" | *wnt11* | *wnt11* | cardiogenesis | PMID: 22974299 |
| ENSGMOG00000014801 | sp|Q92048|WNT2_DANRE Protein Wnt-2 OS=Danio rerio GN=wnt2 PE=2 SV=1" | *wnt2* | *wnt2* | cardiogenesis | PMID: 22449840 |
| ENSGMOG00000000396 | sp|Q2LMP1|WNT3A_CHICK Protein Wnt-3a OS=Gallus gallus GN=WNT3A PE=2 SV=1" | *wnt3a* | *wnt3a* | cardiogenesis | PMID: 22251563 |
| ENSGMOG00000001947 | sp|P28026|WNT8_XENLA Protein Wnt-8 OS=Xenopus laevis GN=wnt8 PE=1 SV=2" | *wnt8* | *wnt8* | cardiogenesis | PMID: 22251563 |
| ENSGMOG00000003521 | sp|P70083|AT2A1_MAKNI Sarcoplasmic/endoplasmic reticulum calcium ATPase 1 OS=Makaira nigricans GN=atp2a1 PE=2 SV=2" | *at2a1* | *atp2a1* | E-C coupling | PMID: 21074636 |
| ENSGMOG00000005903 | sp|P70083|AT2A1_MAKNI Sarcoplasmic/endoplasmic reticulum calcium ATPase 1 OS=Makaira nigricans GN=atp2a1 PE=2 SV=2" | *at2a1* | *atp2a1* | E-C coupling | PMID: 21074636 |
| ENSGMOG00000008137 | sp|Q03669|AT2A2_CHICK Sarcoplasmic/endoplasmic reticulum calcium ATPase 2 OS=Gallus gallus GN=ATP2A2 PE=2 SV=2" | *at2a2* | *atp2a2* | E-C coupling | PMID: 21074636 |
| ENSGMOG00000003047 | sp|Q03669|AT2A2_CHICK Sarcoplasmic/endoplasmic reticulum calcium ATPase 2 OS=Gallus gallus GN=ATP2A2 PE=2 SV=2" | *at2a2* | *atp2a2* | E-C coupling | PMID: 21074636 |
| ENSGMOG00000014735 | sp|P11607|AT2A2_PIG Sarcoplasmic/endoplasmic reticulum calcium ATPase 2 OS=Sus scrofa GN=ATP2A2 PE=2 SV=1" | *at2a2* | *atp2a2* | E-C coupling | PMID: 21074636 |
| ENSGMOG00000007507 | sp|P15999|ATPA_RAT ATP synthase subunit alpha. mitochondrial OS=Rattus norvegicus GN=Atp5a1 PE=1 SV=2" | *atpa* | *atp5a1* | E-C coupling | PMID: 21074636 |
| ENSGMOG00000016407 | sp|Q13936|CAC1C_HUMAN Voltage-dependent L-type calcium channel subunit alpha-1C OS=Homo sapiens GN=CACNA1C PE=1 SV=4" | *cac1c* | *cacna1c* | E-C coupling | PMID: 20657579 |
| ENSGMOG00000015913 | sp|P07221|CASQ1_RABIT Calsequestrin-1 OS=Oryctolagus cuniculus GN=CASQ1 PE=1 SV=1" | *casq1* | *casq1* | E-C coupling | PMID: 26585961 |
| ENSGMOG00000011071 | sp|P19204|CASQ1_CHICK Calsequestrin-1 OS=Gallus gallus GN=CASQ1 PE=1 SV=1" | *casq1* | *casq1* | E-C coupling | PMID: 26585961 |
| ENSGMOG00000015204 | sp|P07221|CASQ1_RABIT Calsequestrin-1 OS=Oryctolagus cuniculus GN=CASQ1 PE=1 SV=1" | *casq1* | *casq1* | E-C coupling | PMID: 26585961 |
| ENSGMOG00000010635 | sp|O09161|CASQ2_MOUSE Calsequestrin-2 OS=Mus musculus GN=Casq2 PE=2 SV=2" | *casq2* | *casq2* | E-C coupling | PMID: 26585961 |
| ENSGMOG00000001723 | sp|O35219|KCNH2_MOUSE Potassium voltage-gated channel subfamily H member 2 OS=Mus musculus GN=Kcnh2 PE=2 SV=2" | *kcnh2* | *kcnh2* | E-C coupling | PMID: 15087427 |
| ENSGMOG00000004695 | sp|Q9PT84|KCNH2_CHICK Potassium voltage-gated channel subfamily H member 2 (Fragment) OS=Gallus gallus GN=KCNH2 PE=2 SV=1" | *kcnh2* | *kcnh2* | E-C coupling | PMID: 15087427 |
| ENSGMOG00000019360 | sp|Q9PT84|KCNH2_CHICK Potassium voltage-gated channel subfamily H member 2 (Fragment) OS=Gallus gallus GN=KCNH2 PE=2 SV=1" | *kcnh2* | *kcnh2* | E-C coupling | PMID: 15087427 |
| ENSGMOG00000004602 | sp|P97414|KCNQ1_MOUSE Potassium voltage-gated channel subfamily KQT member 1 OS=Mus musculus GN=Kcnq1 PE=2 SV=2" | *kcnq1* | *kcnq1* | E-C coupling | PMID: 15087427 |
| ENSGMOG00000007001 | sp|P97414|KCNQ1_MOUSE Potassium voltage-gated channel subfamily KQT member 1 OS=Mus musculus GN=Kcnq1 PE=2 SV=2" | *kcnq1* | *kcnq1* | E-C coupling | PMID: 15087427 |
| ENSGMOG00000018575 | sp|P32418|NAC1_HUMAN Sodium/calcium exchanger 1 OS=Homo sapiens GN=SLC8A1 PE=1 SV=3" | *nac1* | *slc8a1* | E-C coupling | PMID: 24036209 |
| ENSGMOG00000006312 | sp|P23685|NAC1_CANFA Sodium/calcium exchanger 1 OS=Canis familiaris GN=SLC8A1 PE=1 SV=1" | *nac1* | *slc8a1* | E-C coupling | PMID: 24036209 |
| ENSGMOG00000013670 | sp|P48766|NAC1_CAVPO Sodium/calcium exchanger 1 OS=Cavia porcellus GN=SLC8A1 PE=2 SV=1" | *nac1* | *slc8a1* | E-C coupling | PMID: 24036209 |
| ENSGMOG00000000139 | sp|Q01728|NAC1_RAT Sodium/calcium exchanger 1 OS=Rattus norvegicus GN=Slc8a1 PE=2 SV=3" | *nac1* | *slc8a1* | E-C coupling | PMID: 24036209 |
| ENSGMOG00000012679 | sp|Q92736|RYR2_HUMAN Ryanodine receptor 2 OS=Homo sapiens GN=RYR2 PE=1 SV=3" | *ryr2* | *ryr2* | E-C coupling | PMID: 26562359 |
| ENSGMOG00000019367 | sp|P30957|RYR2_RABIT Ryanodine receptor 2 OS=Oryctolagus cuniculus GN=RYR2 PE=1 SV=3" | *ryr2* | *ryr2* | E-C coupling | PMID: 26562359 |
| ENSGMOG00000018839 | sp|Q92736|RYR2_HUMAN Ryanodine receptor 2 OS=Homo sapiens GN=RYR2 PE=1 SV=3" | *ryr2* | *ryr2* | E-C coupling | PMID: 26562359 |
| ENSGMOG00000018612 | sp|Q5R4U9|SORCN_PONAB Sorcin OS=Pongo abelii GN=SRI PE=2 SV=1" | *sorcn* | *sri* | E-C coupling | PMID: 12824171 |
| ENSGMOG00000004332 | sp|Q99250|SCN2A_HUMAN Sodium channel protein type 2 subunit alpha OS=Homo sapiens GN=SCN2A PE=1 SV=3" | *scn2a* | *scn2a* | E-C coupling | PMID: 15087427 |
| ENSGMOG00000001923 | sp|Q99250|SCN2A_HUMAN Sodium channel protein type 2 subunit alpha OS=Homo sapiens GN=SCN2A PE=1 SV=3" | *scn2a* | *scn2a* | E-C coupling | PMID: 15087427 |
| ENSGMOG00000011339 | sp|P53480|ACTC_TAKRU Actin. alpha cardiac OS=Takifugu rubripes PE=2 SV=1" | *actc* | *2* | myofibrillar | PMID: 22974299 |
| ENSGMOG00000001718 | sp|Q805D8|ANF_TAKRU Natriuretic peptides A OS=Takifugu rubripes GN=nppa PE=2 SV=1" | *anf* | *nppa* | myofibrillar | PMID: 24036209 |
| ENSGMOG00000001742 | sp|Q805E8|ANFB_OREMO Brain natriuretic peptide OS=Oreochromis mossambicus GN=nppb PE=2 SV=1" | *anfb* | *nppb* | myofibrillar | PMID: 24036209 |
| ENSGMOG00000001871 | sp|P17661|DESM_HUMAN Desmin OS=Homo sapiens GN=DES PE=1 SV=3" | *desm* | *des* | myofibrillar | PMID: 24036209 |
| ENSGMOG00000007193 | sp|Q3SWY2|ILK_BOVIN Integrin-linked protein kinase OS=Bos taurus GN=ILK PE=2 SV=1" | *ilk* | *ilk* | myofibrillar | PMID: 22974299 |
| ENSGMOG00000001215 | sp|Q3SWY2|ILK_BOVIN Integrin-linked protein kinase OS=Bos taurus GN=ILK PE=2 SV=1" | *ilk* | *ilk* | myofibrillar | PMID: 22974299 |
| ENSGMOG00000007319 | sp|Q9QVP4|MLRA_MOUSE Myosin regulatory light chain 2. atrial isoform OS=Mus musculus GN=Myl7 PE=2 SV=1" | *mlra* | *myl7* | myofibrillar | PMID: 22974299 |
| ENSGMOG00000001561 | sp|P02611|MLRB_CHICK Myosin regulatory light chain 2B. cardiac muscle isoform OS=Gallus gallus PE=1 SV=2" | *mlrb* | *1* | myofibrillar | PMID: 24036209 |
| ENSGMOG00000017657 | sp|P10916|MLRV_HUMAN Myosin regulatory light chain 2. ventricular/cardiac muscle isoform OS=Homo sapiens GN=MYL2 PE=1 SV=3" | *mlrv* | *myl2* | myofibrillar | PMID: 24036209 |
| ENSGMOG00000017490 | sp|A1L260|MURC_DANRE Muscle-related coiled-coil protein OS=Danio rerio GN=murc PE=1 SV=1" | *murc* | *murc* | myofibrillar | PMID: 24036209 |
| ENSGMOG00000010417 | sp|A1L260|MURC_DANRE Muscle-related coiled-coil protein OS=Danio rerio GN=murc PE=1 SV=1" | *murc* | *murc* | myofibrillar | PMID: 24036209 |
| ENSGMOG00000002171 | sp|Q02566|MYH6_MOUSE Myosin-6 OS=Mus musculus GN=Myh6 PE=1 SV=2" | *myh6* | *myh6* | myofibrillar | PMID: 24036209 |
| ENSGMOG00000002258 | sp|Q02566|MYH6_MOUSE Myosin-6 OS=Mus musculus GN=Myh6 PE=1 SV=2" | *myh6* | *myh6* | myofibrillar | PMID: 24036209 |
| ENSGMOG00000003030 | sp|Q02566|MYH6_MOUSE Myosin-6 OS=Mus musculus GN=Myh6 PE=1 SV=2" | *myh6* | *myh6* | myofibrillar | PMID: 24036209 |
| ENSGMOG00000020224 | sp|Q02566|MYH6_MOUSE Myosin-6 OS=Mus musculus GN=Myh6 PE=1 SV=2" | *myh6* | *myh6* | myofibrillar | PMID: 24036209 |
| ENSGMOG00000012704 | sp|A2AQP0|MYH7B_MOUSE Myosin-7B OS=Mus musculus GN=Myh7b PE=3 SV=1" | *myh7b* | *myh7b* | myofibrillar | PMID: 24036209 |
| ENSGMOG00000019765 | sp|P17209|MYL4_RAT Myosin light chain 4 OS=Rattus norvegicus GN=Myl4 PE=2 SV=2" | *myl4* | *myl4* | myofibrillar | PMID: 22974299 |
| ENSGMOG00000014610 | sp|Q32MK0|MYLK3_HUMAN Putative myosin light chain kinase 3 OS=Homo sapiens GN=MYLK3 PE=2 SV=3" | *mylk3* | *mylk3* | myofibrillar | PMID: 24036209 |
| ENSGMOG00000013642 | sp|Q86YV6|MYLK4_HUMAN Myosin light chain kinase family member 4 OS=Homo sapiens GN=MYLK4 PE=1 SV=2" | *mylk4* | *mylk4* | myofibrillar | PMID: 24036209 |
| ENSGMOG00000017147 | sp|Q86YV6|MYLK4_HUMAN Myosin light chain kinase family member 4 OS=Homo sapiens GN=MYLK4 PE=1 SV=2" | *mylk4* | *mylk4* | myofibrillar | PMID: 24036209 |
| ENSGMOG00000018744 | sp|Q90688|MYPC3_CHICK Myosin-binding protein C. cardiac-type OS=Gallus gallus GN=MYBPC3 PE=1 SV=3" | *mypc3* | *mybpc3* | myofibrillar | PMID:26358504 |
| ENSGMOG00000014996 | sp|Q90688|MYPC3_CHICK Myosin-binding protein C. cardiac-type OS=Gallus gallus GN=MYBPC3 PE=1 SV=3" | *mypc3* | *mybpc3* | myofibrillar | PMID:26358504 |
| ENSGMOG00000001449 | sp|P02591|TNNC1_RABIT Troponin C. slow skeletal and cardiac muscles OS=Oryctolagus cuniculus GN=TNNC1 PE=1 SV=1" | *tnnc1* | *tnnc1* | myofibrillar | PMID: 24036209 |
| ENSGMOG00000002857 | sp|P09860|TNNC1_CHICK Troponin C. slow skeletal and cardiac muscles OS=Gallus gallus GN=TNNC1 PE=1 SV=1" | *tnnc1* | *tnnc1* | myofibrillar | PMID: 24036209 |
| ENSGMOG00000001076 | sp|P02642|TNNT2_CHICK Troponin T. cardiac muscle isoforms OS=Gallus gallus GN=TNNT2 PE=2 SV=2" | *tnnt2* | *tnnt2* | myofibrillar | PMID: 22974299, PMID: 24036209 |
| ENSGMOG00000015197 | sp|P50752|TNNT2_MOUSE Troponin T. cardiac muscle OS=Mus musculus GN=Tnnt2 PE=2 SV=2" | *tnnt2* | *tnnt2* | myofibrillar | PMID: 22974299, PMID: 24036209 |
| ENSGMOG00000010706 | sp|P50751|TNNT2_SHEEP Troponin T. cardiac muscle OS=Ovis aries GN=TNNT2 PE=2 SV=2" | *tnnt2* | *tnnt2* | myofibrillar | PMID: 22974299, PMID: 24036209 |
| ENSGMOG00000001057 | sp|P02642|TNNT2_CHICK Troponin T. cardiac muscle isoforms OS=Gallus gallus GN=TNNT2 PE=2 SV=2" | *tnnt2* | *tnnt2* | myofibrillar | PMID: 22974299, PMID: 24036209 |
| ENSGMOG00000018403 | sp|Q9TSZ0|ANGT_CALJA Angiotensinogen OS=Callithrix jacchus GN=AGT PE=2 SV=1" | *angt* | *agt* | vasculogenesis | PMID: 25641373 |
| ENSGMOG00000011415 | sp|P22388|EDN1_RAT Endothelin-1 OS=Rattus norvegicus GN=Edn1 PE=1 SV=2" | *edn1* | *edn1* | vasculogenesis | PMID: 15955745 |
| ENSGMOG00000011016 | sp|P13109|FGF2_RAT Heparin-binding growth factor 2 OS=Rattus norvegicus GN=Fgf2 PE=2 SV=1" | *fgf2* | *fgf2* | vasculogenesis | PMID: 25641373 |
| ENSGMOG00000011858 | sp|Q99PS1|VEGFA_MESAU Vascular endothelial growth factor A OS=Mesocricetus auratus GN=VEGFA PE=2 SV=1" | *vegfa* | *vegfa* | vasculogenesis | PMID: 25641373 |
| ENSGMOG00000016705 | sp|P49767|VEGFC_HUMAN Vascular endothelial growth factor C OS=Homo sapiens GN=VEGFC PE=1 SV=1" | *vegfc* | *vegfc* | vasculogenesis | PMID: 25641373 |
| ENSGMOG00000003602 | sp|P49767|VEGFC_HUMAN Vascular endothelial growth factor C OS=Homo sapiens GN=VEGFC PE=1 SV=1" | *vegfc* | *vegfc* | vasculogenesis | PMID: 25641373 |
| ENSGMOG00000008252 | sp|O43915|VEGFD_HUMAN Vascular endothelial growth factor D OS=Homo sapiens GN=FIGF PE=1 SV=1" | *vegfd* | *figf* | vasculogenesis | PMID: 20371606 |
| ENSGMOG00000010692 | sp|O73682|VGFAA_DANRE Vascular endothelial growth factor A-A OS=Danio rerio GN=vegfaa PE=1 SV=1" | *vgfaa* | *vegfaa* | vasculogenesis | PMID: 25641373 |
| ENSGMOG00000017413 | sp|O73682|VGFAA_DANRE Vascular endothelial growth factor A-A OS=Danio rerio GN=vegfaa PE=1 SV=1" | *vgfaa* | *vegfaa* | vasculogenesis | PMID: 25641373 |
| ENSGMOG00000018994 | sp|Q5GIT4|VGFR2_DANRE Vascular endothelial growth factor receptor 2 OS=Danio rerio GN=kdr PE=1 SV=2" | *vgfr2* | *kdr* | vasculogenesis | PMID: 25641373 |

Supplementary file 1G

| **Swiss prot annoation** | **SP ID** | **GB ID** | **Category** |
| --- | --- | --- | --- |
| sp|P70083|AT2A1_MAKNI Sarcoplasmic/endoplasmic reticulum calcium ATPase 1 OS=Makaira nigricans GN=atp2a1 PE=2 SV=2" | *at2a1* | *atp2a1* | E-C |
| sp|P70083|AT2A1_MAKNI Sarcoplasmic/endoplasmic reticulum calcium ATPase 1 OS=Makaira nigricans GN=atp2a1 PE=2 SV=2" | *at2a1* | *atp2a1* | E-C |
| sp|Q03669|AT2A2_CHICK Sarcoplasmic/endoplasmic reticulum calcium ATPase 2 OS=Gallus gallus GN=ATP2A2 PE=2 SV=2" | *at2a2* | *atp2a2* | E-C |
| sp|Q03669|AT2A2_CHICK Sarcoplasmic/endoplasmic reticulum calcium ATPase 2 OS=Gallus gallus GN=ATP2A2 PE=2 SV=2" | *at2a2* | *atp2a2* | E-C |
| sp|P11607|AT2A2_PIG Sarcoplasmic/endoplasmic reticulum calcium ATPase 2 OS=Sus scrofa GN=ATP2A2 PE=2 SV=1" | *at2a2* | *atp2a2* | E-C |
| sp|P15999|ATPA_RAT ATP synthase subunit alpha. mitochondrial OS=Rattus norvegicus GN=Atp5a1 PE=1 SV=2" | *atpa* | *atp5a1* | E-C |
| sp|Q13936|CAC1C_HUMAN Voltage-dependent L-type calcium channel subunit alpha-1C OS=Homo sapiens GN=CACNA1C PE=1 SV=4" | *cac1c* | *cacna1c* | E-C |
| sp|P19204|CASQ1_CHICK Calsequestrin-1 OS=Gallus gallus GN=CASQ1 PE=1 SV=1" | *casq1* | *casq1* | E-C |
| sp|P07221|CASQ1_RABIT Calsequestrin-1 OS=Oryctolagus cuniculus GN=CASQ1 PE=1 SV=1" | *casq1* | *casq1* | E-C |
| sp|P07221|CASQ1_RABIT Calsequestrin-1 OS=Oryctolagus cuniculus GN=CASQ1 PE=1 SV=1" | *casq1* | *casq1* | E-C |
| sp|O09161|CASQ2_MOUSE Calsequestrin-2 OS=Mus musculus GN=Casq2 PE=2 SV=2" | *casq2* | *casq2* | E-C |
| sp|Q9PT84|KCNH2_CHICK Potassium voltage-gated channel subfamily H member 2 (Fragment) OS=Gallus gallus GN=KCNH2 PE=2 SV=1" | *kcnh2* | *kcnh2* | E-C |
| sp|O35219|KCNH2_MOUSE Potassium voltage-gated channel subfamily H member 2 OS=Mus musculus GN=Kcnh2 PE=2 SV=2" | *kcnh2* | *kcnh2* | E-C |
| sp|Q9PT84|KCNH2_CHICK Potassium voltage-gated channel subfamily H member 2 (Fragment) OS=Gallus gallus GN=KCNH2 PE=2 SV=1" | *kcnh2* | *kcnh2* | E-C |
| sp|P97414|KCNQ1_MOUSE Potassium voltage-gated channel subfamily KQT member 1 OS=Mus musculus GN=Kcnq1 PE=2 SV=2" | *kcnq1* | *kcnq1* | E-C |
| sp|P97414|KCNQ1_MOUSE Potassium voltage-gated channel subfamily KQT member 1 OS=Mus musculus GN=Kcnq1 PE=2 SV=2" | *kcnq1* | *kcnq1* | E-C |
| sp|O73925|KCNQ1_SQUAC Potassium voltage-gated channel subfamily KQT member 1 OS=Squalus acanthias GN=KCNQ1 PE=2 SV=1" | *kcnq1* | *kcnq1* | E-C |
| sp|P30957|RYR2_RABIT Ryanodine receptor 2 OS=Oryctolagus cuniculus GN=RYR2 PE=1 SV=3" | *ryr2* | *ryr2* | E-C |
| sp|Q92736|RYR2_HUMAN Ryanodine receptor 2 OS=Homo sapiens GN=RYR2 PE=1 SV=3" | *ryr2* | *ryr2* | E-C |
| sp|Q92736|RYR2_HUMAN Ryanodine receptor 2 OS=Homo sapiens GN=RYR2 PE=1 SV=3" | *ryr2* | *ryr2* | E-C |
| sp|Q99250|SCN2A_HUMAN Sodium channel protein type 2 subunit alpha OS=Homo sapiens GN=SCN2A PE=1 SV=3" | *scn2a* | *scn2a* | E-C |
| sp|Q99250|SCN2A_HUMAN Sodium channel protein type 2 subunit alpha OS=Homo sapiens GN=SCN2A PE=1 SV=3" | *scn2a* | *scn2a* | E-C |
| sp|P32418|NAC1_HUMAN Sodium/calcium exchanger 1 OS=Homo sapiens GN=SLC8A1 PE=1 SV=3" | *nac1* | *slc8a1* | E-C |
| sp|Q01728|NAC1_RAT Sodium/calcium exchanger 1 OS=Rattus norvegicus GN=Slc8a1 PE=2 SV=3" | *nac1* | *slc8a1* | E-C |
| sp|P23685|NAC1_CANFA Sodium/calcium exchanger 1 OS=Canis familiaris GN=SLC8A1 PE=1 SV=1" | *nac1* | *slc8a1* | E-C |
| sp|P48766|NAC1_CAVPO Sodium/calcium exchanger 1 OS=Cavia porcellus GN=SLC8A1 PE=2 SV=1" | *nac1* | *slc8a1* | E-C |
| sp|Q5R4U9|SORCN_PONAB Sorcin OS=Pongo abelii GN=SRI PE=2 SV=1" | *sorcn* | *sri* | E-C |
| sp|P52186|IRK2_CHICK Inward rectifier potassium channel 2 OS=Gallus gallus GN=KCNJ2 PE=2 SV=1" | *irk2* | *kcnj2* | E-C |
| sp|Q64273|IRK2_RAT Inward rectifier potassium channel 2 OS=Rattus norvegicus GN=Kcnj2 PE=1 SV=1" | *irk* | *kcnj2* | E-C |
| sp|Q4TZY1|IRK12_BOVIN ATP-sensitive inward rectifier potassium channel 12 OS=Bos taurus GN=KCNJ12 PE=2 SV=1" | *irk12* | *kcnj12* | E-C |

Supplementary file 1H:

| **Cod ID** | **Swissprot annotation** | **SP ID** | **GB ID** | **FC E1 H/C** | **FC E2 H/C** | **FC E3 H/C** | **FC E4 H/C** | **FC E5 H/C** | **FC E6 H/C** | **FC L1 H/C** | **FC L2 H/C** | **FC L3 H/C** | **FC L4 H/C** | **FC L5 H/C** |
| --- | --- | --- | --- | --- | --- | --- | --- | --- | --- | --- | --- | --- | --- | --- |
| ENSGMOG00000003521 | sp|P70083|AT2A1_MAKNI Sarcoplasmic/endoplasmic reticulum calcium ATPase 1 OS=Makaira nigricans GN=atp2a1 PE=2 SV=2" | *at2a1* | *atp2a1* | 0 | 0 | 0 | -1.500 | 0 | 0 | 0 | 0 | 0 | 1.312 | 1.327 |
| ENSGMOG00000007507 | sp|P15999|ATPA_RAT ATP synthase subunit alpha. mitochondrial OS=Rattus norvegicus GN=Atp5a1 PE=1 SV=2" | *atpa* | *atp5a1* | 0 | -1.108 | 0 | -1.105 | 0 | 0 | 0 | 0 | 0 | 0 | 1.057 |
| ENSGMOG00000015913 | sp|P07221|CASQ1_RABIT Calsequestrin-1 OS=Oryctolagus cuniculus GN=CASQ1 PE=1 SV=1" | *casq1* | *casq1* | 0 | 0 | 0 | 0 | 0 | 1.304 | 0 | 0 | 0 | 0 | -1.146 |
| ENSGMOG00000008137 | sp|Q03669|AT2A2_CHICK Sarcoplasmic/endoplasmic reticulum calcium ATPase 2 OS=Gallus gallus GN=ATP2A2 PE=2 SV=2" | *at2a2* | *atp2a2* | 0 | -1.160 | 0 | 0 | 0 | 0 | 0 | 0 | 0 | -1.482 | 0 |
| ENSGMOG00000005903 | sp|P70083|AT2A1_MAKNI Sarcoplasmic/endoplasmic reticulum calcium ATPase 1 OS=Makaira nigricans GN=atp2a1 PE=2 SV=2" | *at2a1* | *atp2a1* | 0 | 0 | 0 | 0 | 1.131 | 0 | 0 | 0 | 0 | 1.161 | 0 |
| ENSGMOG00000003047 | sp|Q03669|AT2A2_CHICK Sarcoplasmic/endoplasmic reticulum calcium ATPase 2 OS=Gallus gallus GN=ATP2A2 PE=2 SV=2" | *at2a2* | *atp2a2* | 0 | -1.323 | 0 | 0 | 0 | 0 | 0 | 0 | 0 | 0 | 0 |
| ENSGMOG00000019360 | sp|Q9PT84|KCNH2_CHICK Potassium voltage-gated channel subfamily H member 2 (Fragment) OS=Gallus gallus GN=KCNH2 PE=2 SV=1" | *kcnh2* | *kcnh2* | 0 | 0 | 0 | 0 | -9.033 | 0 | 0 | 0 | 0 | 0 | 0 |
| ENSGMOG00000018575 | sp|P32418|NAC1_HUMAN Sodium/calcium exchanger 1 OS=Homo sapiens GN=SLC8A1 PE=1 SV=3" | *nac1* | *slc8a1* | 0 | 0 | 0 | 0 | 0 | 0 | 0 | 0 | 0 | -4.102 | 0 |
| ENSGMOG00000001923 | sp|Q99250|SCN2A_HUMAN Sodium channel protein type 2 subunit alpha OS=Homo sapiens GN=SCN2A PE=1 SV=3" | *scn2a* | *scn2a* | 0 | 0 | 0 | 0 | 0 | 0 | 0 | 0 | 0 | 1.686 | 0 |
| ENSGMOG00000019367 | sp|P30957|RYR2_RABIT Ryanodine receptor 2 OS=Oryctolagus cuniculus GN=RYR2 PE=1 SV=3" | *ryr2* | *ryr2* | 0 | -1.331 | 0 | 0 | 0 | 0 | 0 | 0 | 0 | 0 | 0 |
| ENSGMOG00000014735 | sp|P11607|AT2A2_PIG Sarcoplasmic/endoplasmic reticulum calcium ATPase 2 OS=Sus scrofa GN=ATP2A2 PE=2 SV=1" | *at2a2* | *atp2a2* | 0 | 0 | 0 | 0 | -1.348 | 0 | 0 | 0 | 0 | 0 | 0 |
| ENSGMOG00000000139 | sp|Q01728|NAC1_RAT Sodium/calcium exchanger 1 OS=Rattus norvegicus GN=Slc8a1 PE=2 SV=3" | *nac1* | *slc8a1* | 0 | -1.825 | 0 | 0 | 0 | 0 | 0 | 0 | 0 | 0 | 0 |
| ENSGMOG00000012072 | sp|P52186|IRK2_CHICK Inward rectifier potassium channel 2 OS=Gallus gallus GN=KCNJ2 PE=2 SV=1" | *irk2* | *kcnj2* | 0 | 0 | 0 | 0 | 0 | 0 | 0 | 0 | 0 | 2.378 | 1.781 |
| ENSGMOG00000005645 | sp|Q64273|IRK2_RAT Inward rectifier potassium channel 2 OS=Rattus norvegicus GN=Kcnj2 PE=1 SV=1" | *irk* | *kcnj2* | 0 | 0 | 0 | 0 | 0 | 0 | 0 | 0 | 0 | 2.553 | 0 |
| ENSGMOG00000003040 | sp|Q4TZY1|IRK12_BOVIN ATP-sensitive inward rectifier potassium channel 12 OS=Bos taurus GN=KCNJ12 PE=2 SV=1" | *irk12* | *kcnj12* | 0 | 0 | 0 | 0 | 0 | 0 | 0 | 0 | 0 | 0 | -5.615 |

Supplementary file 1I

| **Cod ID** | **Swissprot annotation** | **SP ID** | **GB ID** | **FC E1 H/C** | **FC E2 H/C** | **FC E3 H/C** | **FC E4 H/C** | **FC E5 H/C** | **FC E6 H/C** |
| --- | --- | --- | --- | --- | --- | --- | --- | --- | --- |
| ENSGMOG00000000263 | sp|Q12951|FOXI1_HUMAN Forkhead box protein I1 OS=Homo sapiens GN=FOXI1 PE=2 SV=3" | *foxi1* | *foxi1* | 0 | -16.993 | 0 | 0 | 0 | 0 |
| ENSGMOG00000005285 | sp|O14905|WNT9B_HUMAN Protein Wnt-9b OS=Homo sapiens GN=WNT9B PE=1 SV=3" | *wnt9b* | *wnt9b* | 0 | -2.4641 | -1.957 | 0 | 0 | 0 |
| ENSGMOG00000003758 | sp|Q9I8X3|FGFR3_DANRE Fibroblast growth factor receptor 3 OS=Danio rerio GN=fgfr3 PE=2 SV=1" | *fgfr3* | *fgfr3* | 0 | -1.361 | 0 | 0 | 0 | 0 |
| ENSGMOG00000019440 | sp|Q8JG38|FGFR2_DANRE Fibroblast growth factor receptor 2 OS=Danio rerio GN=fgfr2 PE=2 SV=1" | *fgfr2* | *fgfr2* | 0 | -1.166 | -1.599 | -1.613 | 0 | 0 |
| ENSGMOG00000011125 | sp|P10600|TGFB3_HUMAN Transforming growth factor beta-3 OS=Homo sapiens GN=TGFB3 PE=1 SV=1" | *tgfb3* | *tgfb3* | 0 | 0 | -2.975 | -1.984 | 0 | 0 |
| ENSGMOG00000015623 | sp|Q6DFF5|SOX9B_XENLA Transcription factor Sox-9-B OS=Xenopus laevis GN=sox9-b PE=2 SV=1" | *sox9b* | *sox9-b* | 0 | 0 | -1.845 | 0 | 0 | 0 |
| ENSGMOG00000009261 | sp|Q6DFF5|SOX9B_XENLA Transcription factor Sox-9-B OS=Xenopus laevis GN=sox9-b PE=2 SV=1" | *sox9b* | *sox9-b* | 0 | 0 | -1.409 | 0 | 0 | 0 |
| ENSGMOG00000013652 | sp|Q9PUK8|FZD7A_XENLA Frizzled-7-A OS=Xenopus laevis GN=fzd7-a PE=1 SV=2" | *fzd7a* | *fzd7-a* | 0 | 0 | 0 | -1.289 | 0 | 0 |
| ENSGMOG00000013639 | sp|P50576|DLX5A_DANRE Homeobox protein Dlx5a OS=Danio rerio GN=dlx5a PE=2 SV=1" | *dlx5a* | *dlx5a* | 0 | 0 | 1.379 | 0 | 0 | 0 |
| ENSGMOG00000013282 | sp|Q01702|DLX3B_DANRE Homeobox protein Dlx3b OS=Danio rerio GN=dlx3b PE=2 SV=1" | *dlx3b* | *dlx3b* | 0 | 0 | 2.071 | 1.775 | 0 | 0 |
| ENSGMOG00000011415 | sp|P22388|EDN1_RAT Endothelin-1 OS=Rattus norvegicus GN=Edn1 PE=1 SV=2" | *edn1* | *edn1* | 0 | 0 | 8.423 | 3.652 | 0 | 0 |
| ENSGMOG00000013696 | sp|P26014|MYCN_SERCA N-myc proto-oncogene protein OS=Serinus canaria GN=MYCN PE=3 SV=2" | *mycn* | *mycn* | 0 | -1.212 | 0 | 0 | 0 | 0 |

Supplementary file 1J

| **Cod ID** | **Swissprot annotation** | **SP ID** | **SP ID** | **FC E1 H/C** | **FC E2 H/C** | **FC E3 H/C** | **FC E4 H/C** | **FC E5 H/C** | **FC E6 H/C** |
| --- | --- | --- | --- | --- | --- | --- | --- | --- | --- |
| ENSGMOG00000016313 | sp|Q8MJV0|MYH1_HORSE Myosin-1 OS=Equus caballus GN=MYH1 PE=2 SV=1" | *myh1* | *myh1* | 0 | 0 | 0 | 0 | -1.084 | 0 |
| ENSGMOG00000005568 | sp|P35580|MYH10_HUMAN Myosin-10 OS=Homo sapiens GN=MYH10 PE=1 SV=3" | *myh10* | *myh10* | 0 | -1.194 | 0 | 0 | 0 | 0 |
| ENSGMOG00000013798 | sp|P35580|MYH10_HUMAN Myosin-10 OS=Homo sapiens GN=MYH10 PE=1 SV=3" | *myh10* | *myh10* | 0 | 0 | 0 | 0 | 0 | 0 |
| ENSGMOG00000003775 | sp|Q61879|MYH10_MOUSE Myosin-10 OS=Mus musculus GN=Myh10 PE=1 SV=2" | *myh10* | *myh10* | 0 | 0 | -1.302 | 0 | 0 | 0 |
| ENSGMOG00000005651 | sp|P10587|MYH11_CHICK Myosin-11 OS=Gallus gallus GN=MYH11 PE=1 SV=4" | *myh11* | *myh11* | 0 | 0 | 0 | 0 | 0 | 0 |
| ENSGMOG00000009607 | sp|P10587|MYH11_CHICK Myosin-11 OS=Gallus gallus GN=MYH11 PE=1 SV=4" | *myh11* | *myh11* | 0 | 0 | 0 | 0 | 0 | 0 |
| ENSGMOG00000009348 | sp|P02565|MYH3_CHICK Myosin-3 OS=Gallus gallus GN=MYH3 PE=2 SV=3" | *myh3* | *myh3* | 0 | 0 | 0 | -5.346 | 0 | -2.556 |
| ENSGMOG00000001779 | sp|Q076A5|MYH4_CANFA Myosin-4 OS=Canis familiaris GN=MYH4 PE=3 SV=1" | *myh4* | *myh4* | 0 | -1.233 | 0 | 0 | 0 | 0 |
| ENSGMOG00000009387 | sp|Q5SX39|MYH4_MOUSE Myosin-4 OS=Mus musculus GN=Myh4 PE=1 SV=1" | *myh4* | *myh4* | 0 | 0 | -1.924 | -1.847 | -1.976 | 0 |
| ENSGMOG00000011235 | sp|Q8VDD5|MYH9_MOUSE Myosin-9 OS=Mus musculus GN=Myh9 PE=1 SV=4" | *myh9* | *myh9* | 0 | -1.385 | -1.323 | -1.251 | 0 | 0 |
| ENSGMOG00000013979 | sp|P14105|MYH9_CHICK Myosin-9 OS=Gallus gallus GN=MYH9 PE=2 SV=1" | *myh9* | *myh9* | 0 | 0 | 0 | 0 | 0 | 0 |

Supplementary file 1K

| **Cod ID** | **Swissprot annotation** | **SP ID** | **GB ID** | **FC E1 H/C** | **FC E2 H/C** | **FC E3 H/C** | **FC E4 H/C** | **FC E5 H/C** | **FC E6 H/C** | **FC L1 H/C** | **FC L2 H/C** | **FC L3 H/C** | **FC L4 H/C** | **FC L5 H/C** |
| --- | --- | --- | --- | --- | --- | --- | --- | --- | --- | --- | --- | --- | --- | --- |
| ENSGMOG00000005261 | sp|Q9YH26|AT1A1_OREMO Sodium/potassium-transporting ATPase subunit alpha-1 OS=Oreochromis mossambicus GN=atp1a1 PE=2 SV=2" | *at1a1* | *atp1a1* | 0 | -1.761 | -1.477 | -1.394 | 0 | 0 | 0 | 0 | 0 | 0 | 0 |
| ENSGMOG00000014788 | sp|P58312|AT1A3_OREMO Sodium/potassium-transporting ATPase subunit alpha-3 OS=Oreochromis mossambicus GN=atp1a3 PE=2 SV=1" | *at1a3* | *atp1a3* | 0 | -1.322 | 0 | -1.209 | -1.241 | -1.239 | 0 | 0 | 0 | 1.229 | 0 |
| ENSGMOG00000001931 | sp|P58312|AT1A3_OREMO Sodium/potassium-transporting ATPase subunit alpha-3 OS=Oreochromis mossambicus GN=atp1a3 PE=2 SV=1" | *at1a3* | *atp1a3* | 0 | -1.790 | -1.366 | -1.423 | 0 | 0 | 0 | 0 | 0 | 1.289 | 0 |
| ENSGMOG00000018680 | sp|P14415|AT1B2_HUMAN Sodium/potassium-transporting ATPase subunit beta-2 OS=Homo sapiens GN=ATP1B2 PE=1 SV=3" | *at1b2* | *atp1b2* | 0 | 0 | 0 | 0 | -1.979 | -1.667 | 0 | 0 | 0 | 0 | 0 |
| ENSGMOG00000007669 | sp|P14415|AT1B2_HUMAN Sodium/potassium-transporting ATPase subunit beta-2 OS=Homo sapiens GN=ATP1B2 PE=1 SV=3" | *at1b2* | *atp1b2* | 0 | 0 | 0 | 0 | -1.246 | 0 | 0 | 0 | 0 | 1.579 | 1.362 |
| ENSGMOG00000002861 | sp|P26362|CFTR_SQUAC Cystic fibrosis transmembrane conductance regulator OS=Squalus acanthias GN=CFTR PE=2 SV=1" | *cftr* | *cftr* | 0 | -3.372 | -2.741 | -2.259 | 0 | 0 | 0 | 0 | 0 | 0 | 0 |
| ENSGMOG00000004314 | sp|P55011|S12A2_HUMAN Solute carrier family 12 member 2 OS=Homo sapiens GN=SLC12A2 PE=1 SV=1" | *s12a2* | *slc12a2* | 0 | -3.090 | -1.869 | -1.553 | -1.564 | 0 | 0 | 0 | 0 | 0 | 0 |
| ENSGMOG00000013044 | sp|Q28362|SL9A3_DIDMA Sodium/hydrogen exchanger 3 OS=Didelphis marsupialis virginiana GN=SLC9A3 PE=2 SV=1" | *sl9a3* | *slc9a3* | 0 | -2.217 | -3.715 | -2.878 | -1.655 | 0 | 0 | 0 | 0 | 0 | 0 |
| ENSGMOG00000015575 | sp|O43315|AQP9_HUMAN Aquaporin-9 OS=Homo sapiens GN=AQP9 PE=1 SV=2" | *aqp9* | *aqp9* | 0 | 0 | 0 | 0 | 0 | 0 | 0 | 0 | 0 | 1.714 | 1.654 |
| ENSGMOG00000008759 | sp|O54794|AQP7_MOUSE Aquaporin-7 OS=Mus musculus GN=Aqp7 PE=2 SV=1" | *aqp7* | *aqp7* | 0 | 0 | 0 | 0 | 0 | 0 | 0 | 0 | 0 | 3.125 | 0 |
| ENSGMOG00000015825 | sp|P56405|AQP8_RAT Aquaporin-8 OS=Rattus norvegicus GN=Aqp8 PE=2 SV=1" | *aqp8* | *aqp8* | 0 | 0 | 0 | -2.548 | 0 | 0 | 0 | 0 | 0 | 0 | 0 |
| ENSGMOG00000011506 | sp|Q5R819|AQP1_PONAB Aquaporin-1 OS=Pongo abelii GN=AQP1 PE=2 SV=3" | *aqp1* | *aqp1* | 0 | 1.391 | 0 | 0 | 0 | 0 | 0 | 0 | 0 | 0 | 0 |
| ENSGMOG00000006200 | sp|Q8NBQ7|AQP11_HUMAN Aquaporin-11 OS=Homo sapiens GN=AQP11 PE=2 SV=1" | *aqp11* | *aqp11* | 0 | -1.427 | 0 | 0 | 0 | 0 | 0 | 0 | 0 | 0 | 0 |
| ENSGMOG00000008057 | sp|Q08DE6|AQP3_BOVIN Aquaporin-3 OS=Bos taurus GN=AQP3 PE=2 SV=1" | *aqp3* | *aqp3* | 0 | -1.489 | 0 | 0 | 0 | 0 | 0 | 0 | 0 | 0 | 0 |
| ENSGMOG00000015105 | sp|P55088|AQP4_MOUSE Aquaporin-4 OS=Mus musculus GN=Aqp4 PE=2 SV=2" | *aqp4* | *aqp4* | 0 | -7.593 | -13.215 | -6.364 | -2.462 | 0 | 0 | 0 | 0 | 0 | 0 |
| ENSGMOG00000000677 | sp|Q8R2N1|AQP3_MOUSE Aquaporin-3 OS=Mus musculus GN=Aqp3 PE=2 SV=1" | *aqp3* | *aqp3* | 0 | 0 | 0 | 0 | 0 | 0 | 0 | 0 | 0 | -2.637 | 0 |
| ENSGMOG00000019172 | sp|Q8CHJ2|AQP12_MOUSE Aquaporin-12 OS=Mus musculus GN=Aqp12 PE=2 SV=1" | *aqp12* | *aqp12* | 0 | 0 | 0 | 0 | 10.567 | 0 | 0 | 0 | 0 | 0 | -2.864 |
| ENSGMOG00000006554 | sp|P50993|AT1A2_HUMAN Sodium/potassium-transporting ATPase subunit alpha-2 OS=Homo sapiens GN=ATP1A2 PE=1 SV=1" | *at1a2* | *atp1a2* | 0 | 0 | -1.283 | 0 | 1.130 | 0 | 0 | 0 | 0 | 0 | 0 |
| ENSGMOG00000017791 | sp|P21188|AT1B3_XENLA Sodium/potassium-transporting ATPase subunit beta-3 OS=Xenopus laevis GN=atp1b3 PE=2 SV=1" | *at1b3* | *atp1b3* | 0 | 0 | 0 | -1.162 | -1.374 | -1.316 | 0 | 0 | 0 | 1.123 | 1.151 |
| ENSGMOG00000006085 | sp|D3INW7|UT1_CAPHI Urea transporter 1 OS=Capra hircus GN=SLC14A1 PE=2 SV=1" | *ut1* | *slc14a1* | 0 | 0 | 0 | -1.908 | 0 | 0 | 0 | 0 | 0 | 0 | 0 |

Supplementary file 1L

| **Cod ID** | **Swiss prot annotation** | **SP ID** | **GB ID** | **Embrynoic exposure** | **Larval exposure** |
| --- | --- | --- | --- | --- | --- |
| ENSGMOG00000007752 | BOVIN ATP-binding cassette sub-family G member 2 OS=Bos taurus GN=ABCG2 PE=3 SV=2" | *abcg2* | *abcg2* | IE | IE |
| ENSGMOG00000009820 | HUMAN ATP-binding cassette sub-family G member 2 OS=Homo sapiens GN=ABCG2 PE=1 SV=3" | *abcg2* | *abcg2* | IE | IE |
| ENSGMOG00000003981 | HUMAN ATP-binding cassette sub-family G member 2 OS=Homo sapiens GN=ABCG2 PE=1 SV=3" | *abcg2* | *abcg2* | IE | IE |
| ENSGMOG00000008335 | MOUSE ATP-binding cassette sub-family G member 8 OS=Mus musculus GN=Abcg8 PE=2 SV=1" | *abcg8* | *abcg8* | -- | IE |
| ENSGMOG00000011506 | sp|Q5R819|AQP1_PONAB Aquaporin-1 OS=Pongo abelii GN=AQP1 PE=2 SV=3" | *aqp1* | *aqp1* | IE | -- |
| ENSGMOG00000006200 | sp|Q8NBQ7|AQP11_HUMAN Aquaporin-11 OS=Homo sapiens GN=AQP11 PE=2 SV=1" | *aqp11* | *aqp11* | DE | -- |
| ENSGMOG00000019172 | sp|Q8CHJ2|AQP12_MOUSE Aquaporin-12 OS=Mus musculus GN=Aqp12 PE=2 SV=1" | *aqp12* | *Aqp12* | IE | DE |
| ENSGMOG00000008057 | sp|Q08DE6|AQP3_BOVIN Aquaporin-3 OS=Bos taurus GN=AQP3 PE=2 SV=1" | *aqp3* | *aqp3* | DE | -- |
| ENSGMOG00000000677 | sp|Q8R2N1|AQP3_MOUSE Aquaporin-3 OS=Mus musculus GN=Aqp3 PE=2 SV=1" | *aqp3* | *Aqp3* | -- | DE |
| ENSGMOG00000015105 | sp|P55088|AQP4_MOUSE Aquaporin-4 OS=Mus musculus GN=Aqp4 PE=2 SV=2" | *aqp4* | *Aqp4* | DE | -- |
| ENSGMOG00000008759 | sp|O54794|AQP7_MOUSE Aquaporin-7 OS=Mus musculus GN=Aqp7 PE=2 SV=1" | *aqp7* | *aqp7* | -- | IE |
| ENSGMOG00000015825 | sp|P56405|AQP8_RAT Aquaporin-8 OS=Rattus norvegicus GN=Aqp8 PE=2 SV=1" | *aqp8* | *aqp8* | DE | -- |
| ENSGMOG00000015575 | sp|O43315|AQP9_HUMAN Aquaporin-9 OS=Homo sapiens GN=AQP9 PE=1 SV=2" | *aqp9* | *aqp9* | -- | IE |
| ENSGMOG00000005261 | OREMO Sodium/potassium-transporting ATPase subunit alpha-1 OS=Oreochromis mossambicus GN=atp1a1 PE=2 SV=2" | *at1a1* | *atp1a1* | DE | -- |
| ENSGMOG00000006554 | HUMAN Sodium/potassium-transporting ATPase subunit alpha-2 OS=Homo sapiens GN=ATP1A2 PE=1 SV=1" | *at1a2* | *atp1a2* | DE/IE | -- |
| ENSGMOG00000014788 | OREMO Sodium/potassium-transporting ATPase subunit alpha-3 OS=Oreochromis mossambicus GN=atp1a3 PE=2 SV=1" | *at1a3* | *atp1a3* | DE | IE |
| ENSGMOG00000001931 | OREMO Sodium/potassium-transporting ATPase subunit alpha-3 OS=Oreochromis mossambicus GN=atp1a3 PE=2 SV=1" | *at1a3* | *atp1a3* | DE | IE |
| ENSGMOG00000007669 | HUMAN Sodium/potassium-transporting ATPase subunit beta-2 OS=Homo sapiens GN=ATP1B2 PE=1 SV=3" | *at1b2* | *atp1b2* | DE | IE |
| ENSGMOG00000018680 | HUMAN Sodium/potassium-transporting ATPase subunit beta-2 OS=Homo sapiens GN=ATP1B2 PE=1 SV=3" | *at1b2* | *atp1b2* | DE | -- |
| ENSGMOG00000017791 | XENLA Sodium/potassium-transporting ATPase subunit beta-3 OS=Xenopus laevis GN=atp1b3 PE=2 SV=1" | *at1b3* | *atp1b3* | DE | IE |
| ENSGMOG00000003966 | ANGAN Sodium/potassium-transporting ATPase subunit beta-233 OS=Anguilla anguilla GN=atnb233 PE=1 SV=1" | *at233* | *atnb233* | DE | -- |
| ENSGMOG00000007206 | ANGAN Sodium/potassium-transporting ATPase subunit beta-233 OS=Anguilla anguilla GN=atnb233 PE=1 SV=1" | *at233* | *atnb233* | DE | -- |
| ENSGMOG00000003521 | MAKNI Sarcoplasmic/endoplasmic reticulum calcium ATPase 1 OS=Makaira nigricans GN=atp2a1 PE=2 SV=2" | *at2a1* | *atp2a1* | DE | IE |
| ENSGMOG00000005903 | MAKNI Sarcoplasmic/endoplasmic reticulum calcium ATPase 1 OS=Makaira nigricans GN=atp2a1 PE=2 SV=2" | *at2a1* | *atp2a1* | IE | IE/DE |
| ENSGMOG00000006390 | HUMAN Plasma membrane calcium-transporting ATPase 1 OS=Homo sapiens GN=ATP2B1 PE=1 SV=3" | *at2b1* | *atp2b1* | DE | IE |
| ENSGMOG00000003730 | RAT Plasma membrane calcium-transporting ATPase 2 OS=Rattus norvegicus GN=Atp2b2 PE=2 SV=2" | *at2b2* | *atp2b2* | DE | -- |
| ENSGMOG00000006083 | RAT Plasma membrane calcium-transporting ATPase 2 OS=Rattus norvegicus GN=Atp2b2 PE=2 SV=2" | *at2b2* | *atp2b2* | DE | -- |
| ENSGMOG00000003698 | OREMO Plasma membrane calcium-transporting ATPase 2 (Fragment) OS=Oreochromis mossambicus GN=atp2b2 PE=2 SV=1" | *at2b2* | *atp2b2* | DE | -- |
| ENSGMOG00000012854 | HUMAN Plasma membrane calcium-transporting ATPase 4 OS=Homo sapiens GN=ATP2B4 PE=1 SV=2" | *at2b4* | *atp2b4* | DE | DE |
| ENSGMOG00000001173 | ONCMY Band 3 anion exchange protein OS=Oncorhynchus mykiss GN=slc4a1 PE=2 SV=2" | *b3at* | *slc4a1* | DE | -- |
| ENSGMOG00000002861 | SQUAC Cystic fibrosis transmembrane conductance regulator OS=Squalus acanthias GN=CFTR PE=2 SV=1" | *cftr* | *cftr* | DE | -- |
| ENSGMOG00000016856 | BOVIN Solute carrier family 2. facilitated glucose transporter member 1 OS=Bos taurus GN=SLC2A1 PE=2 SV=1" | *gtr1* | *slc2a1* | DE | -- |
| ENSGMOG00000001236 | RAT Solute carrier family 2. facilitated glucose transporter member 1 OS=Rattus norvegicus GN=Slc2a1 PE=1 SV=1" | *gtr1* | *slc2a1* | -- | IE |
| ENSGMOG00000020485 | HUMAN ATP-sensitive inward rectifier potassium channel 1 OS=Homo sapiens GN=KCNJ1 PE=1 SV=1" | *irk1* | *kcnj1* | DE | -- |
| ENSGMOG00000019748 | HUMAN ATP-sensitive inward rectifier potassium channel 15 OS=Homo sapiens GN=KCNJ15 PE=1 SV=2" | *irk15* | *kcnj15* | DE | -- |
| ENSGMOG00000020584 | HUMAN Inward rectifier potassium channel 16 OS=Homo sapiens GN=KCNJ16 PE=2 SV=1" | *irk16* | *kcnj16* | DE | -- |
| ENSGMOG00000012618 | HUMAN Calcium-activated potassium channel subunit alpha-1 OS=Homo sapiens GN=KCNMA1 PE=1 SV=2" | *kcma1* | *kcnma1* | DE/IE | -- |
| ENSGMOG00000000234 | HUMAN Calcium-activated potassium channel subunit alpha-1 OS=Homo sapiens GN=KCNMA1 PE=1 SV=2" | *kcma1* | *kcnma1* | IE | -- |
| ENSGMOG00000018629 | HUMAN Potassium channel subfamily K member 2 OS=Homo sapiens GN=KCNK2 PE=2 SV=2" | *kcnk2* | *kcnk2* | DE | -- |
| ENSGMOG00000017254 | RAT Potassium channel subfamily K member 9 OS=Rattus norvegicus GN=Kcnk9 PE=1 SV=2" | *kcnk9* | *kcnk9* | DE | -- |
| ENSGMOG00000004471 | CRIGR Ileal sodium/bile acid cotransporter OS=Cricetulus griseus GN=SLC10A2 PE=2 SV=1" | *ntcp2* | *slc10a2* | -- | DE |
| ENSGMOG00000008496 | RAT Solute carrier family 12 member 1 OS=Rattus norvegicus GN=Slc12a1 PE=2 SV=1" | *s12a1* | *slc12a1* | DE | -- |
| ENSGMOG00000004314 | HUMAN Solute carrier family 12 member 2 OS=Homo sapiens GN=SLC12A2 PE=1 SV=1" | *s12a2* | *slc12a2* | DE | -- |
| ENSGMOG00000018797 | DANRE Solute carrier family 12 member 9 OS=Danio rerio GN=slc12a9 PE=3 SV=1" | *s12a9* | *slc12a9* | DE/IE | -- |
| ENSGMOG00000000949 | RABIT Solute carrier family 22 member 2 OS=Oryctolagus cuniculus GN=SLC22A2 PE=1 SV=1" | *s22a2* | *slc22a2* | -- | DE |
| ENSGMOG00000019467 | RAT Solute carrier family 22 member 5 OS=Rattus norvegicus GN=Slc22a5 PE=1 SV=1" | *s22a5* | *slc22a5* | DE | -- |
| ENSGMOG00000012478 | BOVIN Solute carrier family 22 member 7 OS=Bos taurus GN=SLC22A7 PE=2 SV=1" | *s22a7* | *slc22a7* | DE | -- |
| ENSGMOG00000014380 | HUMAN Solute carrier family 22 member 13 OS=Homo sapiens GN=SLC22A13 PE=2 SV=2" | *s22ad* | *slc22a13* | IE | IE |
| ENSGMOG00000002642 | XENLA Solute carrier family 22 member 16 OS=Xenopus laevis GN=slc22a16 PE=2 SV=2" | *s22ag* | *slc22a16* | -- | IE |
| ENSGMOG00000003912 | BOVIN Sodium-driven chloride bicarbonate exchanger OS=Bos taurus GN=SLC4A10 PE=2 SV=1" | *s4a10* | *slc4a10* | -- | IE |
| ENSGMOG00000014112 | BOVIN Electrogenic sodium bicarbonate cotransporter 1 OS=Bos taurus GN=SLC4A4 PE=1 SV=1" | *s4a4* | *slc4a4* | DE/IE | -- |
| ENSGMOG00000008159 | RAT Electrogenic sodium bicarbonate cotransporter 4 OS=Rattus norvegicus GN=Slc4a5 PE=1 SV=1" | *s4a5* | *slc4a5* | DE | -- |
| ENSGMOG00000002663 | RAT Sodium bicarbonate cotransporter 3 OS=Rattus norvegicus GN=Slc4a7 PE=1 SV=1" | *s4a7* | *slc4a7* | DE | IE |
| ENSGMOG00000011067 | MOUSE Sodium/myo-inositol cotransporter OS=Mus musculus GN=Slc5a3 PE=2 SV=1" | *sc5a3* | *slc5a3* | IE | -- |
| ENSGMOG00000013044 | DIDMA Sodium/hydrogen exchanger 3 OS=Didelphis marsIEialis virginiana GN=SLC9A3 PE=2 SV=1" | *sl9a3* | *slc9a3* | DE | -- |
| ENSGMOG00000017116 | HUMAN Solute carrier organic anion transporter family member 1C1 OS=Homo sapiens GN=SLCO1C1 PE=2 SV=1" | *so1c1* | *slco1c1* | -- | IE |
| ENSGMOG00000018947 | MOUSE Solute carrier organic anion transporter family member 2A1 OS=Mus musculus GN=Slco2a1 PE=1 SV=2" | *so2a1* | *slco2a1* | -- | IE |
| ENSGMOG00000002784 | MOUSE Solute carrier organic anion transporter family member 2B1 OS=Mus musculus GN=Slco2b1 PE=1 SV=1" | *so2b1* | *slco2b1* | -- | IE |

Supplementary file 1M:

| **Cod ID** | **Swisprot annotation** | **SP ID** | **GB ID** |
| --- | --- | --- | --- |
| ENSGMOG00000019019 | sp|Q5IS79|ATOH1_PANTR Protein atonal homolog 1 OS=Pan troglodytes GN=ATOH1 PE=2 SV=1" | *atoh1* | *atoh1* |
| ENSGMOG00000006684 | sp|P48985|ATOH1_MOUSE Protein atonal homolog 1 OS=Mus musculus GN=Atoh1 PE=2 SV=1" | *atoh1* | *atoh1* |
| ENSGMOG00000019782 | sp|Q5IS79|ATOH1_PANTR Protein atonal homolog 1 OS=Pan troglodytes GN=ATOH1 PE=2 SV=1" | *atoh1* | *atoh1* |
| ENSGMOG00000019416 | sp|Q9I8L5|FA10A_DANRE Fatty acid-binding protein 10-A, liver basic OS=Danio rerio GN=fabp10a PE=1 SV=1" | *fa10a* | *fabp10a* |
| ENSGMOG00000012026 | sp|Q9I8L5|FA10A_DANRE Fatty acid-binding protein 10-A, liver basic OS=Danio rerio GN=fabp10a PE=1 SV=1" | *fa10a* | *fabp10a* |
| ENSGMOG00000000286 | sp|Q6R8J2|HEG_DANRE Protein HEG OS=Danio rerio GN=heg PE=2 SV=1" | *heg* | *heg* |
| ENSGMOG00000004754 | sp|Q5RDH6|TFR1_PONAB Transferrin receptor protein 1 OS=Pongo abelii GN=TFRC PE=2 SV=1" | *tfr1* | *tfrc* |
| ENSGMOG00000017342 | sp|Q9GLD3|TFR1_CANFA Transferrin receptor protein 1 OS=Canis familiaris GN=TFRC PE=1 SV=1" | *tfr1* | *tfrc* |
| ENSGMOG00000014801 | sp|Q92048|WNT2_DANRE Protein Wnt-2 OS=Danio rerio GN=wnt2 PE=2 SV=1" | *wnt2* | *wnt2* |
| ENSGMOG00000007840 | sp|Q98SN7|WNT2B_CHICK Protein Wnt-2b OS=Gallus gallus GN=WNT2B PE=2 SV=1" | *wnt2b* | *wnt2b* |
| ENSGMOG00000006778 | sp|Q98SN7|WNT2B_CHICK Protein Wnt-2b OS=Gallus gallus GN=WNT2B PE=2 SV=1" | *wnt2b* | *wnt2b* |
| ENSGMOG00000011697 | sp|Q8AWG6|HHEX_XENTR Hematopoietically-expressed homeobox protein hhex OS=Xenopus tropicalis GN=hhex PE=2 SV=1" | *hhex* | *hhex* |
| ENSGMOG00000008433 | sp|Q07DY1|MET_NOMLE Hepatocyte growth factor receptor OS=Nomascus leucogenys GN=MET PE=3 SV=1" | *met* | *met* |

Supplementary file 1N

| **SP ID** | **Forward primer 5´-3´** | **Reverse primer 5´-3´** | **Probe 5´-3´** | **Quencher** |
| --- | --- | --- | --- | --- |
| *wnt11* | AACCCCTACACCGAGAAAC | CTCGACTATCCTTTCGCAC | FAM-TGGTGCTGCTACGTCACCTGCAAGAA | Tamra |
| *kcnh2* | ACGTGAACCACAACGACGAG | AGCCAGAGCGGAAGATAAGCAG | FAM-TTACATGACCGGGTGGTTCGCCATCGACCTTT | Tamra |
| *nac1* | CCGCCCTCATCTACATGTTC | ACGTGATGACCTCGATGGAG | FAM-ATGTCCATCATCGCCGACCGCTTCAT | Tamra |
| *cacn1c* | ATGAGAACAGCTCCCTCAC | ATGATGACGGCGACAAAC | FAM-GCAGCAATTTTGCCATATTCTACTTTGTCAGCTTC | Tamra |
| *at2a2* | TGTTCCCAGTATGAAGCCC | CACCTTCTCAAACACACCC | FAM-TGCAACGACTCATCGCTGGACTTCAAC | Tamra |
| *cp1a* | CCTCCTTCCTGCCCTTCAC | TTGGGAATGAAGTAGCCATTGA | 6FAM-CCTCACTGCGCCACAAAAGACACATC | Tamra |
| *ef1a* | ATCGGCGGTATCGGAACAG | GCTTGAGGACACCGGTCTCA | 6FAM-ACCCGTGGGCCGTG | None |
